# Synthesis of Trifluoroacetyl‐Substituted Cyclopropanes Using Onium Ylides

**DOI:** 10.1002/ejoc.201701699

**Published:** 2018-01-22

**Authors:** Michael Winter, Christina Gaunersdorfer, Lukas Roiser, Katharina Zielke, Uwe Monkowius, Mario Waser

**Affiliations:** ^1^ Institute of Organic Chemistry Johannes Kepler University Linz Altenbergerstr. 69 4040 Linz Austria; ^2^ School of Education Johannes Kepler University Linz Altenbergerstr. 69 4040 Linz Austria

**Keywords:** Cyclopropanation, Fluorine, Ylides, Diastereomers, Synthetic methods

## Abstract

The use of carbonyl‐stabilized ammonium‐ and sulfonium ylides allows for the synthesis of highly‐functionalized trifluoroacetyl‐substituted cyclopropanes. It turned out that the diastereoselectivities strongly depend on the nature of the chosen ylide and the employed base. The products could be obtained in good yields under operationally simple conditions.

## Introduction

**Scheme 1:**
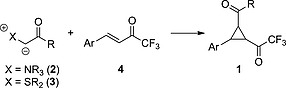
Targeted ylide‐mediated synthesis of trifluoroacetyl‐containing cyclopropanes **1**.

Fluorinated organic compounds are an important class of molecules, mainly because of their unique properties for a variety of different applications (i.e. medicinal chemistry, material chemistry, imaging technologies)^1^ Accordingly, the development of new synthesis strategies to access small (chiral) F‐containing molecules, that may serve as building blocks for further manipulations, has become a heavily investigated task.^2^ Among the different small‐ring organic skeletons, cyclopropanes play an important role due to their unique ring strain‐driven reactivity^3^ and also because of their presence in biologically active molecules.^4^ The (asymmetric) synthesis of cyclopropanes has thus been a longstanding important topic^5^ and fluorine‐containing cyclopropanes have become increasingly important over the last years.^6^ An efficient strategy to access (chiral) cyclopropanes is the addition of ammonium or sulfonium ylides to Michael acceptors.[5a], 7, 8, 9 More recently also some reports towards the synthesis of F‐containing cyclopropanes via onium ylides have been reported.^10^ Our group has a long‐standing research focus on (asymmetric) ammonium ylide‐mediated cyclization reactions.^11^, ^12^ Given the interest in F‐containing cyclopropanes, we thus became interested in testing the potential of ammonium and sulfonium ylides to access trifluoroacetyl‐containing cyclopropanes **1**. These highly functionalized cyclopropanes have so far only sparingly been investigated,^13^ and we therefore rationalized that an addition of carbonyl‐stabilized ammonium or sulfonium ylides **2** and **3** to the easily accessible Michael acceptors **4** would result in a powerful method to access a variety of these highly functionalized target molecules (Scheme 1).

## Results and Discussion

**Figure 1:**
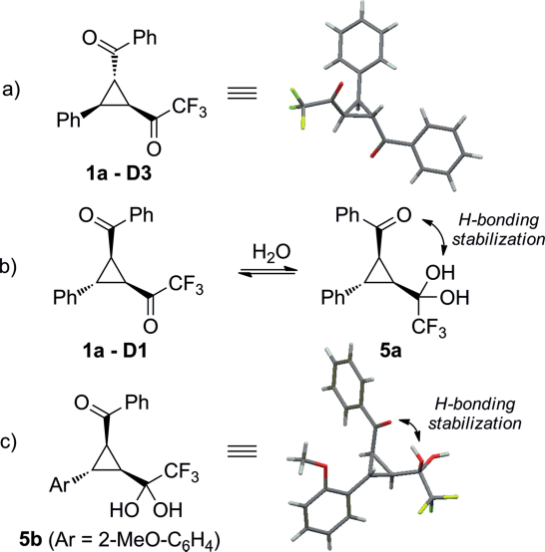
Single crystal analysis of diastereomer D3 of cyclopropane **1a** and the hydrate stabilization observed for the acetophenon‐trifluoroacetyl‐*cis*‐configured diastereomers D1.^15^

**Scheme 2:**
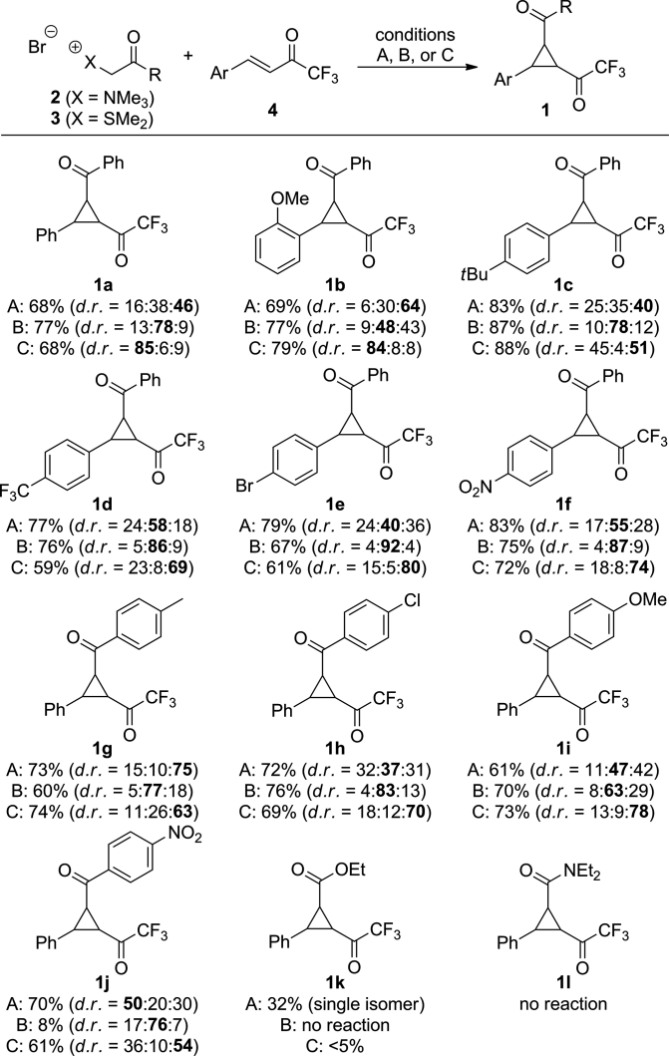
Application scope: **Cond. A**: 1:1 ratio of **2** and **4** (0.1 mmol scale) and 2 equiv. Cs_2_CO_3_ in CH_2_Cl_2_ at room temp. for 20 h; **Cond. B**: 1:1 ratio of **2** and **4** (0.1 mmol scale) and 10 equiv. DBU in CH_2_Cl_2_ at room temp. for 20 h; **Cond. C**: 1.2:1 ratio of **3** and **4** (0.1 mmol scale) and 2 equiv. Cs_2_CO_3_ in CH_2_Cl_2_ at room temp. for 20 h.

We started our investigations by using the achiral acetophenone‐based ammonium‐ and sulfonium salts **2** and **3** as ylide‐precursor for the cyclopropanation reaction with acceptor **4a**. Table 1 gives an overview of the most significant results obtained in a broad screening of different conditions. For the ammonium salts **2**, Me_3_N was chosen as the amine component because of its proven superior leaving group ability compared to other achiral tertiary amines.[11b], [11d] An initial screening of different solvents and temperatures revealed that the reactions are best carried out in CH_2_Cl_2_ at room temperature for 20 h. This was also carefully double‐checked with the optimized conditions and it turned out that longer reaction times and/or higher temperatures lead to a pronounced decomposition of the product **1a** under the basic conditions. While the use of sodium‐ and potassium carbonate did not result in any product formation (entries 1 and 2), the use of an excess of K_3_PO_4_ lead to the slow formation of a mixture of the three *trans*,*trans*,*cis*‐diastereomers (D1, D2, D3) of cyclopropane **1a** (entry 3). Switching to solid Cs_2_CO_3_ next, the conversion could be increased significantly (entries 4–8). It turned out that 2 equiv. of this base leads to a reasonable combined 68 % isolated yield of the three diastereomers (entry 4), while a higher amount resulted in a more pronounced by‐product formation (entry 6). The three diastereomers were obtained in slightly varying ratios depending on the amount of base and also depending on the stoichiometric ratio of ammonium salt **2** and acceptor **4a** (entries 7 and 8). In most of these Cs_2_CO_3_ experiments D3 turned out to be the slightly favored diastereomer and we were also able to analyze this isomer by single‐crystal X‐ray analysis, which revealed the relative *trans*,*trans*,*cis*‐configuration depicted in Figure 1a. At this point it should be noted that in neither case (also with the other derivatives shown in Scheme 2) the *all*‐*cis* diastereomer could be detected. Unfortunately, we have not been able to crystallize the other two diastereomers of **1a**. However, we realized that D1 always contains noticeable amounts of its corresponding hydrate **5a** (which is also visible by the upfield ^19^F‐shift of the CF_3_‐NMR signal) and the same was also observed for some of the other derivatives shown in Scheme 2. We rationalized that the presence of such a hydrate may be a consequence of H‐bonding stabilization because of a *cis*‐oriented carbonyl group, which would only be possible for one specific *trans*,*trans*,*cis*‐diastereomer as illustrated in Figure 1b. Luckily, we have later been able to crystallize the hydrate (**5b**) of the *o*‐methoxy‐containing cyclopropane **1b**, which proved this *cis*‐configuration of the trifluoroacetyl group and the acetophenone group (Figure 1c) and we therefore assigned the relative configuration of other diastereomers D1 that are in equilibrium with the hydrate in analogy.

**Table 1:** Identification of the optimum reaction conditions for the cyclopropanation of compound **4a** a

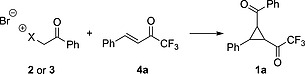					
Entry	X	Base	Conversion	*dr* (D1/D2/D3)^c^	Yield
		(equiv.)	[%]^b^		[%]^d^
1	NMe_3_	Na_2_CO_3_ (2 ×)	no conv.	n.d.	n.d.
2	NMe_3_	K_2_CO_3_ (2 ×)	no conv.	n.d.	n.d.
3	NMe_3_	K_3_PO_4_ (10 ×)	40	28:38:34	n.d.
**4**	NMe_3_	Cs_2_CO_3_ (2 ×)	**> 99**	**16:38:46**	**68**
5	NMe_3_	Cs_2_CO_3_ (1 ×)	60	43:30:27	n.d.
6	NMe_3_	Cs_2_CO_3_ (10 ×)	> 99	16:37:47	49
7^e^	NMe_3_	Cs_2_CO_3_ (2 ×)	> 99	10:38:52	70
8^f^	NMe_3_	Cs_2_CO_3_ (2 ×)	> 99	52:38:9	< 50
9	NMe_3_	NaOH (1 ×)	> 99	32:34:34	86
10	NMe_3_	DBU (2 ×)	> 99	12:53:35	86
**11**	NMe_3_	**DBU (10 ×)**	**> 99**	**13:78:9**	**77**
12	SMe_2_ g	DBU (10 ×)	> 99	9:80:11	59
13	SMe_2_ g	NaOH (1 ×)	> 99	86:6:8	< 50
**14**	SMe_2_ g	Cs_2_CO_3_ (2 ×)	**> 99**	**85:6:9**	**68**

a All reactions were carried out with a 1:1 ratio of ylide precursors **2** or **3** and acceptor **4a** (unless otherwise stated) on 0.1 mmol scale in CH_2_Cl_2_ at room temp. for 20 h.

b Determined by ^1^H NMR of the crude reaction product.

c Determined by ^1^H NMR; only the three *trans*,*trans*,*cis*‐diastereomers were detected, not the *all*‐*cis*.

d Combined isolated yield of all diastereomers.

e Using 2 equiv. of ylide precursor **2a**.

f Using 2 equiv. of acceptor **4a**.

g 1.2 equiv. of sulfonium salt **3a**.

When we used stronger inorganic bases like solid NaOH, **1a** could be obtained in high yield but with no pronounced selectivity for any of the diastereomers (entry 9). Here an excess of base or the use of an aqueous base lead to a significantly reduced yield. By changing for organic bases, it was only with DBU that we observed a reasonable formation of the cyclopropane **1a** (entries 10, 11). Very interestingly, while 2 equiv. of DBU resulted in a slight preference of D2, an excess of DBU lead to an increased selectivity towards this diastereomer (entry 11). In a next attempt, we used the sulfonium salt **3a** for this reaction (entries 12–14). Noteworthy, a similar result as for the ammonium salt **2a** was observed under the DBU‐conditions (compare entries 11 and 12). However, when changing for NaOH or Cs_2_CO_3_ as bases (entries 13 and 14), we observed a strong preference for the D1 diastereomer in this sulfonium ylide‐mediated cyclopropanation. This complementary outcome illustrates very nicely the well‐documented different reactivity trends of ammonium and sulfonium ylides, which are a consequence of the different nucleophilicities and leaving group abilities of these ylides.^14^

Summing this screening up, we have identified complementary conditions that give the diastereomers with different preferences by using ammonium or sulfonium salts in the presence of different bases. The most promising conditions (highlighted in bold in Table 1) were then also used to elucidate the application scope of this cyclopropanation reaction (Scheme 2).

Testing different acceptors **4** as well as differently substituted acetophenone‐based ammonium and sulfonium salts **2** and **3** first (giving cyclopropanes **1a**–**1j**), it turned out that the (electronic) nature of the reagents can have a pronounced influence on the observed diastereoselectivities under the three different sets of conditions. The use of solid Cs_2_CO_3_ with the ammonium ylides (Cond. A, compare with entry 4, Table 1) resulted in rather mixed diastereomeric ratios for all targets and in no case apart from **1g** a higher preference for one of the three diastereomers was observed. When using DBU (Cond. B, entry 11, Table 1), we observed a rather high preference for D2 in most cases and apart from the nitro‐substituted cyclopropane **1j** all target compounds were obtained in reasonably high yields. Interesting results were obtained with the sulfonium salts **3** (Cond. C, entry 14, Table 1) which favoured D1 only for the parent target **1a** and the methoxy‐compound **1b** but then resulted in preferred formation of D3 for the other substrates (D3 of **1j** could be crystallized and showed the same relative configuration as D3 of **1a** and **1b**!). We also tested the potential of ester‐ and amide‐stabilized ylides to access the cyclopropanes **1k** and **1l**. Unfortunately, those ylides were found to be less suited for these reactions. We have not been able to obtain the amide‐based **1l** under any conditions and the ester‐containing **1k** could only be accessed in low yields (but with a very high diastereomeric purity) when using the corresponding ammonium salt in the presence of Cs_2_CO_3_. We were however not able to increase the yield by optimizing the reaction conditions.

**Scheme 3:**
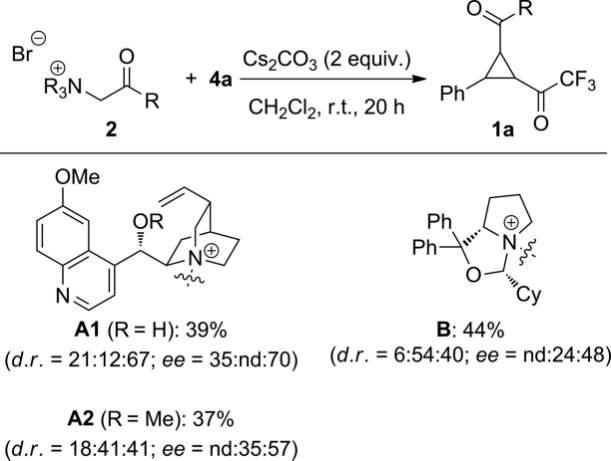
Attempted enantioselective cyclopropanation.

Finally, we investigated the use of chiral amine‐leaving groups for the enantioselective synthesis of cyclopropane **1a** (Scheme 3). Cinchona alkaloids are usually the chiral amines of choice for most asymmetric ammonium ylide‐mediated cyclizations^7^, ^12^, ^16^ and we thus tested a variety of different derivatives **A** first. As illustrated for the two examples shown in Scheme 3, the yields were not very high in these cases and no improvement by prolonged reactions time or other changes were possible. The diastereomeric ratios were in the same ranges as for the achiral approaches and the highest enantiomeric excess that could be obtained was 70 % *ee* for the major diastereomer when using **A1**. As we have recently shown that the proline‐based bicyclic chiral amines **B** are superior leaving groups for ammonium ylide‐mediated epoxidations,[12a] we also tested these. Unfortunately, the outcome was not satisfying and we thus carried out no further optimizations with these chiral auxiliaries. Thus, despite a first proof‐of‐concept for an asymmetric version of this reaction was obtained, we were not able to reach the high enantioselectivities that we observed for other ammonium ylide‐mediated cyclizations in the past.^12^

## Conclusions

We have shown that the use of differently substituted carbonyl‐stabilized ammonium‐ and sulfonium ylides allows for the synthesis of highly‐functionalized trifluoroacetyl‐substituted cyclopropanes in high yields. It was found that the diastereoselectivities in these reactions strongly depend on the nature of the chosen ylide and the employed base, thus leading to operationally simple and complementary conditions. A first proof‐of‐concept for an asymmetric protocol was obtained as well but with yet relatively low yields and medium selectivities.

## Experimental Section

**Supporting Information** (see footnote on the first page of this article): General details as well as the analytical details and characterization data of all the novel cyclopropanes can be found in the online supporting information.

**General Cyclopropanation Procedure:** To a stirred suspension of the base (2 equiv. Cs_2_CO_3_ or 10 equiv. DBU) in 3 mL of CH_2_Cl_2_, the ammonium salt **2** (0.1 mmol) (or 0.12 mmol sulfonium salt **3**) and the acceptor **4** (0.1 mmol) were added. The reaction mixture was stirred for 20 h at room temperature. Subsequently, the reaction was quenched by addition of 10 mL of H_2_O_deion_ and extracted three times with 10 mL of EtOAc. The combined organic phases were dried with Na_2_SO_4_ and the solvents evaporated to dryness. The crude product was purified by column chromatography over silica gel with gradients of heptane/EtOAc (100:0 – 20:1 – 10:1 – 5:1 – 2:1) to yield the cyclopropanes **1** in the reported yields.

## Supporting information

Supporting Information
