# Supplementary material for: Synthesis of Trifluoroacetyl‐Substituted Cyclopropanes Using Onium Ylides
Source: European J Org Chem. 2018 Jan 22;2018(3):418–21. doi: 10.1002/ejoc.201701699 (PMC5817241; doi:10.1002/ejoc.201701699)
Supplement: Supplementary file 1 — Supporting Information [file EJOC-2018-418-s001.pdf]

Eur. J. Org. Chem. · ISSN 1099–0690

https://doi.org/10.1002/ejoc.201701699

SUPPORTING INFORMATION

Title: Molecular Structure and Crystal Packing of Monofluoromethoxyarenes

Author(s): Michael Winter, Christina Gaunersdorfer, Lukas Roiser, Katharina Zielke, Uwe Monkowius, Mario Waser*

1. General Information: ... 3
1.1. General Methods ... 3
1.2. X-Ray Analysis ... 4

2. Syntheses ... 5
2.1 General Cyclopropanation Procedures ... 5
2.2 Analytical Details of Cyclopropanes 1 ... 6
2.3 Asymmetric Cyclopropanation ... 13

4. Copies of NMR-Spectra of new Compounds ... 14

1. General Information:

1.1. General Methods

^{1}H- and ^{13}C-NMR spectra were recorded on a Bruker Avance III 300 MHz spectrometer with a broad band observe probe and a sample changer for 16 samples and on a Bruker Avance III 700 MHz spectrometer with with an Ascend magnet and TCI cryoprobe, which are both property to the Austro-Czech NMR-Research Center “RERI-uasb”. All NMR spectra were referenced on the solvent peak. High resolution mass spectra were obtained using an Agilent 6520 Q-TOF mass spectrometer with an ESI source and an Agilent G1607A coaxial sprayer or a Thermo Fisher Scientific LTQ Orbitrap XL with an Ion Max API Source. Analyses were made in the positive or negative ionization mode as stated in the specific cases. Purine (exact mass for $[M+H]^+$ = 121.050873) and 1,2,3,4,5,6-hexakis(2,2,3,3-tetrafluoropropoxy)-1,3,5,2,4,6-triaza-triphosphinane (exact mass for $[M+H]^+$ = 922.009798) were used for internal mass calibration.

Preparative column chromatography was carried out using Davisil LC 60A 70-200 MICRON silica gel. TLC probes were detected at 254 nm or stained with with an appropriate staining solution (compare section 3.1.3).

HPLC was performed using a Dionex Summit HPLC system with a Chiralcel AD-H (250 x 4.6 mm, 10 µm) chiral stationary phase.

All chemicals were purchased from commercial suppliers and used without further purification unless otherwise stated. All reactions were carried out under Argon.

The acceptors **4** are literature known and were synthesized as described previously.[1] The known ammonium salts **2** and sulfonium salts **3** were synthesized in analogy to recent procedures.[2,3]

---

1) a) P. Suman, T. N. Rao, B. C. Raju, *Helvetica Chimica Acta* **2013**, 96, 1548-1559; b) Y. Wang, J. Han, J. Chen, Weiguo Cao, *Tetrahedron* **2015**, 71, 8256-8262.
2) N. Meisinger, L. Roiser, U. Monkowius, M. Himmelsbach, R. Robiette, M. Waser, *Chem. Eur. J.*, **2017**, *23*, 5137-5142.
3) a) K. W. Ratts, A. N. Yao, *J. Org. Chem*. **1966**, *31*, 1185-1188; b) E. Grange, V. K. Aggarwal, *Chem. Eur. J.* **2006**, 12, 568-575.

1.2. X-Ray Analysis

Single-crystal structure analyses were carried out on a Bruker Smart X2S (**1a-D3**) and on a Bruker D8 Quest Eco diffractometer operating with Mo-K_{α} radiation (λ= 0.71073 Å). Further crystallographic and refinement data can be found in Table 1. The structures were solved by direct methods (SHELXS-97)[4] and refined by full-matrix least squares on F^2 (SHELXL-97).[5] The H atoms were calculated geometrically, and a riding model was applied in the refinement process. For **5b**, the hydrogen atom engaged in the intramolecular hydrogen bondwas refined. CCDC 1588560-1588563 contain the supplementary crystallographic data for compound **1a-D3**, **1f-D3**, **1b-D3**, and **5b**. These data can be obtained free of charge from The Cambridge Crystallographic Data Centre at www.ccdc.cam.ac.uk.

Table 1: Crystal Data and Data Collection and Structure Refinement Details for Compounds **1a-D3**, **1f-D3**, **1b-D3**, and **5b**.

**Crystal Data**	**1a-D3**	**1f-D3**	**1b-D3**	**5b**
Empirical formula	$C_{18}H_{13}F_3O_2$	$C_{18}H_{12}F_3NO_4$	$C_{19}H_{15}F_3O_3$	$C_{19}H_{17}F_3O_4$
Formula weight	318.28	363.29	348.31	366.33
Crystal size (mm)	0.64 × 0.21 × 0.12	0.41 × 0.33 × 0.10	1.11 × 0.55 × 0.08	0.60 × 0.13 × 0.10
Crystal system	monoclinic	monoclinic	monoclinic	monoclinic
Space group	$P2_1/c$	$P\bar{1}$	$P2_1/c$	$P2_1/c$
a (Å)	5.5711(7)	9.7495(5)	11.770(2)	5.3147(3)
b (Å)	16.096(2)	10.0184(5)	7.9978(14)	27.0493(14)
c (Å)	17.395(2)	10.4433(5)	18.560(3)	12.2015(7)
α (deg)	90	100.157(2)	90	90
β(deg)	96.646(4)	117.110(2)	103.865	100.721(3)
γ (deg)	90	107.080(2)	90	90
V (Å^3)	1549.4(3)	808.20(7)	1696.2(5)	1723.46(16)
D_{calcd} (g cm^{-1})	1.365	1.493	1.364	1.412
Z	4	2	4	4
μ (mm^{-1})	0.11	0.13	0.11	0.12
T (K)	296	296	296	296
θ range (°)	2.5-23.4	2.4-23.4	2.8-26.4	2.8-26.4
No. of reflections measured	31110	25406	27458	34316
No. of independent reflections	3143	2348	3480	3519
Obs. Reflections [$I > 2\sigma(I)$]	1947	1720	1308	2042
Parameters refined/restraints	236/0	264/12	227/0	241
Absorption correction	multi-scan	multi-scan	multi-scan	multi-scan
T_{min}, T_{max}	0.35, 0.99	0.95, 0.99	0.88, 0.99	0.93, 0.99
max/min σ_{fin} (e Å^{-3})	0.12/-0.16	0.12/-0.14	0.39/-0.25	0.21/-0.25
R_1 [$I \geq 2\sigma(I)$]	0.048	0.046	0.124	0.100
wR_2	0.128	0.098	0.231	0.145
CCDC no.	1588560	1588561	1588562	1588563

4 G. M. Sheldrick, *SHELXS-97, Program for the Solution of Crystal Structures*, Göttingen, Germany, 1997. See also: G. M. Sheldrick, *Acta Crystallographica*, 1990, **A46**, 467-473.
5 G. M. Sheldrick, *SHELXL-97, Program for crystal structure refinement*, Göttingen, Germany, 1997. See also: G. M. Sheldrick, *Acta Crystallographica*, 2008, **A64**, 112-122.

2. Syntheses

2.1 General Cyclopropanation Procedures

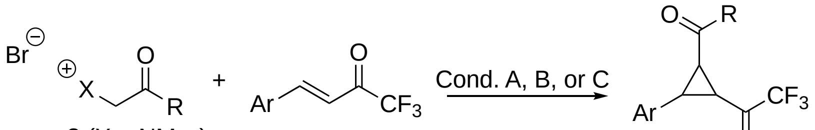

2 (X = NMe3)
3 (X = SMe2)
4
1

Conditions A: To a stirred suspension of Cs_2CO_3 (2 eq.) in 3 mL DCM, the ammonium salt **2** (0.1 mmol) and the acceptor **4** (0.1 mmol) were added. The reaction mixture was stirred for 20 h at room temperature. Subsequently, the reaction was quenched by addition of 10 mL water and extracted three times with 10 mL ethyl acetate. The combined organic phases were dried with dry sodium sulfate and evaporated to dryness. The crude product was purified by column chromatography over silica gel with gradients of heptane-ethyl acetate (100:0 – 20:1 – 10:1 – 5:1 – 2:1) to yield the cyclopropanes in the reported yields.

Conditions B: To a stirred mixture of DBU (10 eq.) in 3 mL DCM, the ammonium salt **2** (0.1 mmol) and the acceptor **4** (0.1 mmol) were added. The reaction mixture was stirred for 20 h at room temperature. Subsequently, the reaction was quenched by addition of 10 mL water and extracted three times with 10 mL ethyl acetate. The combined organic phases were dried with dry sodium sulfate and evaporated to dryness. The crude product was purified by column chromatography over silica gel with gradients of heptane-ethyl acetate (100:0 – 20:1 – 10:1 – 5:1 – 2:1) to yield the cyclopropanes in the reported yields.

Conditions C: To a stirred suspension of Cs_2CO_3 (2 eq.) in 3 mL DCM, the sulfonium salt **3** (0.12 mmol) and the acceptor **4** (0.1 mmol) were added. The reaction mixture was stirred for 20 h at room temperature. Subsequently, the reaction was quenched by addition of 10 mL water and extracted three times with 10 mL ethyl acetate. The combined organic phases were dried with dry sodium sulfate and evaporated to dryness. The crude product was purified by column chromatography over silica gel with gradients of heptane-ethyl acetate (100:0 – 20:1 – 10:1 – 5:1 – 2:1) to yield the cyclopropanes in the reported yields.

2.2 Analytical Details of Cyclopropanes 1

1a: Isolated in the yields and with the diastereomeric ratios reported in Scheme 2 in the main manuscript under the different conditions.

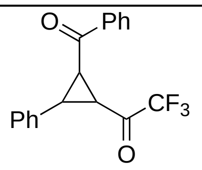

1a
A: 68% (*d.r.* = 16:38:**46**)
B: 77% (*d.r.* = 13:**78**:9)
C: 68% (*d.r.* = **85**:6:9)

HRMS (ESI): *m/z* calculated for $C_{18}H_{14}F_3O_2^+$: 319.0940 [M+H]$^+$; found: 319.0943 (measured from the crude reaction mixture).

1a-D1: ^{1}H-NMR (300 MHz, $CDCl_3$, 298 K): δ = 7.97 (d, J = 7.4 Hz, 2H), 7.60 (t, J = 7.4 Hz, 1H), 7.48 (t, J = 7.4 Hz, 2H), 7.41-7.32 (m, 4H), 7.24-7.22 (m, 1H), 3.52-3.49 (m, 2H), 2.98 (dd, J^1 = 6.7 Hz, J^2 = 8.5 Hz, 1H) ppm; ^{19}F-NMR (282 MHz, $CDCl_3$, 298 K): δ = -78.17 (s, 3F) ppm.

1a-D2: ^{1}H-NMR (700 MHz, $CDCl_3$, 298 K): δ = 7.96 (d, J = 7.6 Hz, 2H), 7.59 (t, J = 7.7 Hz, 1H), 7.47 (t, J = 7.7 Hz, 2H), 7.24-7.20 (m, 3H), 7.16 (d, J = 7.1 Hz, 2H), 3.83 (dd, J^1 = 5.8 Hz, J^2 = 10.6 Hz, 1H), 3.82 (dd, J^1 = 5.8 Hz, J^2 = 6.5 Hz, 1H), 3.49 (dd, J^1 = 6.5 Hz, J^2 = 10.6 Hz, 1H) ppm; ^{19}F-NMR (282 MHz, $CDCl_3$, 298 K): δ = -78.61 (s, 3F) ppm; ^{13}C-NMR (76 MHz, $CDCl_3$, 298 K): δ = 191.8 (**C**=O), 189.7 (q, J = 37.0 Hz, **C**O-CF_3), 137.0 (Ar-**C**), 136.0 (Ar-**C**), 133.9 (Ar-**C**), 132.7 (Ar-**C**), 129.0 (Ar-**C**), 128.7 (Ar-**C**), 128.6 (Ar-**C**), 128.5 (Ar-**C**), 128.1 (Ar-**C**), 115.9 (q, J = 293.9 Hz, **C**F_3), 39.9 (**C**H), 38.1 (**C**H), 28.1 (**C**H) ppm.

1a-D3: ^{1}H-NMR (700 MHz, $CDCl_3$, 298 K): δ = 8.10 (d, J = 9.4 Hz, 3H), 7.66 (t, J = 7.4 Hz, 1H), 7.56 (t,, J = 7.9 Hz, 2H), 7.34 (t, J = 7.9 Hz, 2H), 7.30-7.28 (m, 3H), 4.13 (dd, J^1 = 4.9 Hz, J^2 = 6.7 Hz, 1H), 3.62 (dd, J^1 = 6.7 Hz, J^2 = 9.7 Hz, 1H), 3.49 (dd, J^1 = 4.9 Hz, J^2 = 9.7 Hz, 1H) ppm; ^{19}F-NMR (282 MHz, $CDCl_3$, 298 K): δ = -79.08 (s, 3F) ppm; ^{13}C-NMR (176 MHz, $CDCl_3$, 298 K): δ = 195.3 (**C**=O), 185.6 (q, J = 36.4 Hz, **C**O-CF_3), 136.6 (Ar-**C**), 134.2 (Ar-**C**), 132.2 (Ar-**C**), 129.2 (Ar-**C**), 129.2 (Ar-**C**), 128.8 (Ar-**C**), 128.6 (Ar-**C**), 128.3 (Ar-**C**), 115.5 (q, J = 295.2 Hz, **C**F_3), 40.2 (**C**H), 34.0 (**C**H), 30.8 (**C**H) ppm. IR (film): $\tilde{\nu}$ = 2959, 2924, 2857, 2363, 2345, 1750, 1680, 1454, 1379, 1269, 1213, 1155, 1068, 1053, 1015, 981, 910 cm^{-1}.

1b: Isolated in the yields and with the diastereomeric ratios reported in Scheme 2 in the main manuscript under the different conditions.

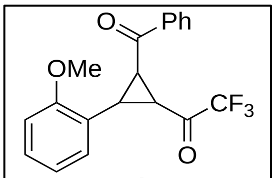

1b
A: 69% (*d.r.* = 6:30:**64**)
B: 77% (*d.r.* = 9:**48**:43)
C: 79% (*d.r.* = **84**:8:8)

HRMS (ESI): m/z calculated for $C_{19}H_{16}F_3O_3^+$: 349.1046 [M+H]$^+$; found: 349.1048 (measured from the crude reaction mixture).

1b-D1: ^{1}H-NMR (700 MHz, $CDCl_3$, 298 K): δ = 8.00 (d, J = 7.6 Hz, 3H), 7.63 (t, J = 7.2 Hz, 1H), 7.50 (t, J = 7.9 Hz, 2H), 7.32-7.29 (m, 2H), 6.88 (d, J = 8.2 Hz, 1H), 3.72 (s, 3H), 3.58 (dd, J^1 = 6.7 Hz, J^2 = 6.7 Hz, 1H), 3.58 (dd, J^1 = 6.7 Hz, J^2 = 9.0 Hz, 1H), 3.01 (dd, J^1 = 6.7 Hz, J^2 = 9.0 Hz, 1H) ppm; ^{19}F-NMR (282 MHz, $CDCl_3$, 298 K): δ = -78.19 (s, 3F) ppm; ^{13}C-NMR (176 MHz, $CDCl_3$, 298 K): δ = 193.4 (**C**=O), 187.1 (q, J = 35.4 Hz, **C**O-CF_3), 136.1 (Ar-**C**), 133.7 (Ar-**C**), 129.0 (Ar-**C**), 128.7 (Ar-**C**), 128.6 (Ar-**C**), 127.4 (Ar-**C**), 124.7 (Ar-

C), 120.6 (Ar-**C**), 115.9 (q, J = 292.0 Hz, **C**F_3), 110.6 (Ar-**C**), 55.3 (**C**H_3), 37.7 (**C**H), 31.0 (**C**H), 28.5 (**C**H) ppm.

1b-D2: ^{1}H-NMR (300 MHz, $CDCl_3$, 298 K): δ = 8.01 (d, J = 7.2 Hz, 2H), 7.58 (t, J = 7.3 Hz, 1H), 7.48 (t, J = 7.8 Hz, 2H), 7.23-7.13 (m, 2H), 6.89 (t, J = 7.3 Hz, 1H), 6.69 (d, J = 7.9 Hz, 1H), 3.88 (dd, J^1 = 4.7 Hz, J^2 = 10.1 Hz, 1H), 3.71 (dd, J^1 = 4.1 Hz, J^2 = 6.2 Hz, 1H), 3.47 (s, 3H), 3.39 (dd, J^1 = 6.2 Hz, J^2 = 10.1 Hz, 1H) ppm; ^{19}F-NMR (282 MHz, $CDCl_3$, 298 K): δ = -78.56 (s, 3F) ppm; ^{13}C-NMR (76 MHz, $CDCl_3$, 298 K): δ = 192.0 (**C**=O), 189.7 (q, J = 37.8 Hz, **C**O-CF_3), 157.7 (Ar-**C**), 137.0 (Ar-**C**), 133.3 (Ar-**C**), 130.0 (Ar-**C**), 129.2 (Ar-**C**), 128.5 (Ar-**C**), 128.4 (Ar-**C**), 126.3 (q, J = 289.2 Hz, **C**F_3), 120.6 (Ar-**C**), 109.9 (Ar-**C**), 54.6 (**C**H_3), 37.0 (**C**H), 36.3 (**C**H), 28.5 (**C**H) ppm.

1b-D3: ^{1}H-NMR (700 MHz, $CDCl_3$, 298 K): δ = 8.12 (d, J = 7.0 Hz, 2H), 7.66 (t, J = 7.3 Hz, 1H), 7.56 (t, J = 7.9 Hz, 2H), 7.30 (t, J = 7.9 Hz, 2H), 7.00 (t, J = 7.0 Hz, 1H), 6.82 (d, J = 8.4 Hz, 1H), 3.93 (dd, J^1 = 4.9 Hz, J^2 = 6.5 Hz, 1H), 3.78 (s, 3H), 3.51 (dd, J^1 = 6.5 Hz, J^2 = 9.6 Hz, 1H), 3.43 (dd, J^1 = 4.9 Hz, J^2 = 9.6 Hz, 1H) ppm; ^{19}F-NMR (282 MHz, $CDCl_3$, 298 K): δ = -78.14 (s, 3F) ppm; ^{13}C-NMR (176 MHz, $CDCl_3$, 298 K): δ = 195.4 (**C**=O), 185.7 (q, J = 37.9 Hz, **C**O-CF_3), 157.4 (Ar-**C**), 136.5 (Ar-**C**), 133.9 (Ar-**C**), 130.6 (Ar-**C**), 129.5 (Ar-**C**), 128.9 (Ar-**C**), 128.4 (Ar-**C**), 121.1 (Ar-**C**), 120.4 (Ar-**C**), 119.5 (q, J = 289.8 Hz, **C**F_3), 110.1 (Ar-**C**), 55.0 (**C**H_3), 35.8 (**C**H), 32.6 (**C**H), 32.5 (**C**H) ppm. IR (film): $\tilde{\nu}$ = 3071, 2951, 2843, 2365, 2340, 1744, 1678, 1601, 1584, 1495, 1462, 1449, 1444, 1371, 1327, 1269, 1255, 1209, 1184, 1153, 1119, 1047, 1022, 987, 937, 889 cm^{-1}.

5b: ^{1}H-NMR (700 MHz, $CDCl_3$, 298 K): δ = 8.00 (d, J = 7.8 Hz, 2H), 7.63 (t, J = 7.3 Hz, 1H), 7.50 (t, J = 7.9 Hz, 2H), 7.31 (t, J = 7.9 Hz, 2H), 6.88 (d, J = 8.0 Hz, 1H), 3.72 (s, 3H), 3.33 (dd, J^1 = 6.7 Hz, J^2 = 6.7 Hz, 1H), 3.05 (dd, J^1 = 6.7 Hz, J^2 = 9.0 Hz, 1H), 2.55 (dd, J^1 = 6.7 Hz, J^2 = 9.0 Hz, 1H) ppm; ^{19}F-NMR (282 MHz, $CDCl_3$, 298 K): δ = -85.60 (s, 3F) ppm.

1c: Isolated in the yields and with the diastereomeric ratios reported in Scheme 2 in the main manuscript under the different conditions.

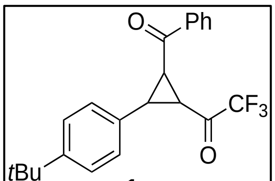

1c

A: 83% (*d.r.* = 25:35:**40**)
B: 87% (*d.r.* = 10:**78**:12)
C: 88% (*d.r.* = 45:4:**51**)

HRMS (ESI): *m/z* calculated for $C_{22}H_{22}F_3O_2^+$: 375.1566 [M+H]$^+$; found: 375.1568 (measured from the crude reaction mixture).

1c-D1 (could not be isolated in pure form, therefore only the 19F signal measured from the crude mixture is given): ^{19}F-NMR (282 MHz, $CDCl_3$, 298 K): δ = -78.18 (s, 3F) ppm**.**

1c-D2: ^{1}H-NMR (300 MHz, $CDCl_3$, 298 K): δ = 7.97 (d, J = 7.7 Hz, 2H), 7.58 (t, J = 7.4 Hz, 1H), 7.47 (t, J = 7.2 Hz, 2H), 7.24 (d, J = 9.9 Hz, 2H), 7.09 (d, J = 8.3 Hz, 2H), 3.72 (s, 3H), 3.81 (dd, J^1 = 6.4 Hz, J^2 = 6.4 Hz, 1H), 3.80 (dd, J^1 = 6.4 Hz, J^2 = 9.6 Hz, 1H), 3.44 (dd, J^1 = 6.4 Hz, J^2 = 9.6 Hz, 1H), 1.23 (s, 9H) ppm; ^{19}F-NMR (282 MHz, $CDCl_3$, 298 K): δ = -78.61 (s, 3F) ppm; ^{13}C-NMR (76 MHz, $CDCl_3$, 298 K): δ = 193.4 (**C**=O), 187.1 (q, J = 35.4 Hz, **C**O-CF_3), 136.1 (Ar-**C**), 133.7 (Ar-**C**), 129.0 (Ar-**C**), 128.7 (Ar-**C**), 128.6 (Ar-**C**), 127.4 (Ar-**C**), 124.7 (Ar-**C**), 120.6 (Ar-**C**), 115.9 (q, J = 292.0 Hz, **C**F_3), 110.6 (Ar-**C**), 39.8 (**C**H), 39.2 (**C**-C_4), 38.2 (**C**H), 32.4 (**C**H_3), 31.3 (**C**H_3), 31.2 (**C**H_3), 28.2 (**C**H) ppm.

1c-D3: ^{1}H-NMR (700 MHz, $CDCl_3$, 298 K): δ = 8.10 (d, J = 7.6 Hz, 2H), 7.66 (t, J = 7.3 Hz, 1H), 7.55 (t, J = 7.7 Hz, 2H), 7.35 (d, J = 8.2 Hz, 2H), 7.22 (d, J = 8.2 Hz, 2H), 4.12 (dd, J^1 = 4.9 Hz, J^2 = 6.2 Hz, 1H), 3.51 (dd, J^1 = 6.2 Hz, J^2 = 9.6 Hz, 1H), 3.49 (dd, J^1 = 4.9 Hz, J^2 = 9.6 Hz, 1H), 1.30 (s, 9H) ppm; ^{19}F-NMR (282 MHz, $CDCl_3$, 298 K): δ = -79.01 (s, 3F) ppm; ^{13}C-NMR (176 MHz, $CDCl_3$, 298 K): δ = 195.5 (**C**=O), 185.5 (q, J = 36.8 Hz, **C**O-CF_3), 151.3 (Ar-**C**), 136.6 (Ar-**C**), 134.2 (Ar-**C**), 130.2 (Ar-**C**), 129.1 (Ar-**C**), 129.0 (Ar-**C**), 128.8 (Ar-**C**), 128.6 (Ar-**C**), 125.7 (Ar-**C**), 115.4 (q, J = 290.9 Hz, **C**F_3), 53.6 (**C**-C_4), 40.2 (**C**H), 34.7 (**C**H), 34.0 (**C**H), 31.4 (**C**H_3), 31.2 (**C**H_3), 31.0 (**C**H_3) ppm. IR (film): $\tilde{\nu}$ = 2970, 2866, 2365, 2338, 1750, 1682, 1601, 1577, 1508, 1452, 1375, 1362, 1331, 1274, 1205, 1145, 1063, 1020, 1009, 984 cm^{-1}.

<u>**1d**</u>: Isolated in the yields and with the diastereomeric ratios reported in Scheme 2 in the main manuscript under the different conditions.

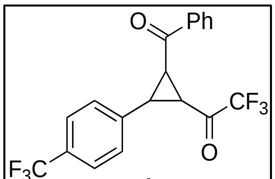

1d
A: 77% (*d.r.* = 24:**58**:18)
B: 76% (*d.r.* = 5:**86**:9)
C: 59% (*d.r.* = 23:8:**69**)

HRMS (ESI): *m/z* calculated for $C_{19}H_{13}F_6O_2^+$: 387.0814 $[M+H]^+$; found: 387.0826 (measured from the crude reaction mixture).

1d-D1: ^{1}H-NMR (300 MHz, $CDCl_3$, 298 K): δ = 7.95 (d, J = 8.0 Hz, 2H), 7.66-7.59 (m, 3H), 7.53-7.46 (m, 3H), 7.37 (d, J = 8.1 Hz, 2H), 3.55-3.52 (m, 2H), 3.01 (dd, J^1 = 6.8 Hz, J^2 = 8.4 Hz, 1H) ppm; ^{19}F-NMR (282 MHz, $CDCl_3$, 298 K): δ = -62.64 (s, 3F), -78.14 (s, 3F) ppm.

1d-D2: ^{1}H-NMR (700 MHz, $CDCl_3$, 298 K): δ = 7.96 (d, J = 7.4 Hz, 2H), 7.61 (t, J = 7.4 Hz, 1H), 7.50-7.48 (m, 4H), 7.29 (d, J = 8.3 Hz, 2H), 3.86 (dd, J^1 = 4.5 Hz, J^2 = 6.3 Hz, 1H), 3.85 (dd, J^1 = 4.5 Hz, J^2 = 9.7 Hz, 1H), 3.50 (dd, J^1 = 6.3 Hz, J^2 = 9.7 Hz, 1H) ppm; ^{19}F-NMR (282 MHz, $CDCl_3$, 298 K): δ = -62.74 (s, 3F, Ar-C**F**$_3$), -78.63 (s, 3F) ppm; ^{13}C-NMR (176 MHz, $CDCl_3$, 298 K): δ = 191.5 (**C**=O), 189.2 (q, J = 37.5 Hz, **C**O-CF_3), 136.7 (Ar-**C**), 134.2 (Ar-**C**), 130.3 (q, J = 32.7 Hz, Ar-**C**F_3), 129.2 (Ar-**C**), 129.1 (Ar-**C**), 128.5 (Ar-**C**), 127.4 (Ar-**C**), 125.6 (Ar-**C**), 115.7 (q, J = 289.2 Hz, **C**F_3), 38.8 (**C**H), 37.7 (**C**H), 28.2 (**C**H) ppm.

1d-D3: ^{1}H-NMR (700 MHz, $CDCl_3$, 298 K): δ = 8.10 (d, J = 7.3 Hz, 2H), 7.66 (t, J = 7.6 Hz, 1H), 7.55 (t, J = 8.2 Hz, 2H), 7.35 (d, J = 8.2 Hz, 2H), 7.22 (d, J = 8.2 Hz, 2H), 4.12 (dd, J^1 = 4.9 Hz, J^2 = 6.2 Hz, 1H), 3.57 (dd, J^1 = 6.2 Hz, J^2 = 9.6 Hz, 1H), 3.50 (dd, J^1 = 4.9 Hz, J^2 = 9.6 Hz, 1H) ppm; ^{19}F-NMR (282 MHz, $CDCl_3$, 298 K): δ = -62.72 (s, 3F, ArC**F**$_3$), -79.12 (s, 3F) ppm; ^{13}C-NMR (176 MHz, $CDCl_3$, 298 K): δ = 195.4 (**C**=O), 185.6 (q, J = 38.9 Hz, **C**O-CF_3), 151.2 (Ar-**C**), 136.6 (Ar-**C**), 134.2 (Ar-**C**), 130.2 (Ar-**C**), 129.1 (Ar-**C**), 129.0 (Ar-**C**), 128.8 (Ar-**C**), 128.6 (Ar-**C**), 125.7 (Ar-**C**), 115.5 (q, J = 286.5 Hz, **C**F_3), 90.49 (Ar-**C**), 40.2 (**C**H), 34.0 (**C**H), 31.2 (**C**H) ppm. IR (film): $\tilde{\nu}$ = 3071, 2947, 2870, 2365, 2345, 1749, 1682, 1620, 1597, 1580, 1449, 1379, 1329, 1275, 1215, 1165, 1149, 1122, 1067, 1020, 989, 891 cm^{-1}.

<u>**1e**</u>: Isolated in the yields and with the diastereomeric ratios reported in Scheme 2 in the main manuscript under the different conditions.

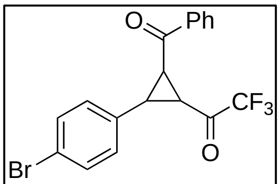

1e
A: 79% (*d.r.* = 24:**40**:36)
B: 67% (*d.r.* = 4:**92**:4)
C: 61% (*d.r.* = 15:5:**80**)

HRMS (ESI): *m/z* calculated for $C_{18}H_{11}BrF_3O_2^-$: 394.9900 $[M-H]^-$; found: 394.9904 (measured from the crude reaction mixture).

1e-D1 (only isolated in minor quantities coeluding with the major diastereomers, therefore only the 19F signal measured from the crude mixture is given): ^{19}F-NMR (282 MHz, $CDCl_3$, 298 K): δ = -78.18 (s, 3F) ppm.

1e-D2: ^{1}H-NMR (700 MHz, $CDCl_3$, 298 K): δ = 7.95 (d, J = 7.4 Hz, 2H), 7.60 (t, J = 7.4 Hz, 1H), 7.48 (t, J = 7.7 Hz, 2H), 7.34 (d, J = 8.4 Hz, 2H), 7.03 (d, J = 8.3 Hz, 2H), 3.81 (dd, J^1 = 4.8 Hz, J^2 = 10.0 Hz, 1H), 3.78 (dd, J^1 = 4.8 Hz, J^2 = 5.9 Hz, 1H), 3.42 (dd, J^1 = 5.9 Hz, J^2 = 10.0 Hz, 1H); ^{19}F-NMR (282 MHz, $CDCl_3$, 298 K): δ = -78.63 (s, 3F) ppm; ^{13}C-NMR (176 MHz, $CDCl_3$, 298 K): δ = 191.6 (**C**=O), 189.4 (q, J = 36.8 Hz, **C**O-CF_3), 136.8 (Ar-**C**), 134.1 (Ar-**C**), 131.8 (Ar-**C**), 131.7 (Ar-**C**), 131.0 (Ar-**C**), 129.1 (Ar-**C**), 128.5 (Ar-**C**), 122.2 (Ar-**C**), 115.8 (q, J = 289.3 Hz, **C**F_3), 38.9 (**C**H), 37.8 (**C**H), 28.1 (**C**H) ppm.

1e-D3: ^{1}H-NMR (700 MHz, $CDCl_3$, 298 K): δ = 8.09 (d, J = 7.4 Hz, 2H), 7.67 (t, J = 7.4 Hz, 1H), 7.56 (t, J = 7.9 Hz, 2H), 7.47 (d, J = 8.4 Hz, 2H), 7.16 (d, J = 8.2 Hz, 2H), 4.08 (dd, J^1 = 5.0 Hz, J^2 = 6.8 Hz, 1H), 3.56 (dd, J^1 = 6.8 Hz, J^2 = 9.7 Hz, 1H), 3.48 (dd, J^1 = 5.0 Hz, J^2 = 9.7 Hz, 1H) ppm; ^{19}F-NMR (282 MHz, $CDCl_3$, 298 K): δ = -79.11 (s, 3F) ppm; ^{13}C-NMR (176 MHz, $CDCl_3$, 298 K): δ = 194.9 (**C**=O), 185.5 (q, J = 36.8 Hz, **C**O-CF_3), 136.4 (Ar-**C**), 134.4 (Ar-**C**), 131.9 (Ar-**C**), 131.2 (Ar-**C**), 130.8 (Ar-**C**), 129.2 (Ar-**C**), 128.6 (Ar-**C**), 122.4 (Ar-**C**), 115.3 (q, J = 290.3 Hz, **C**F_3), 39.2 (**C**H), 33.8 (**C**H), 30.7 (**C**H) ppm. IR (film): $\tilde{\nu}$ = 3061, 2962, 2926, 2853, 2360, 2341, 1743, 1673, 1597, 1580, 1491, 1450, 1427, 1375, 1325, 1269, 1206, 1147, 1093, 1074, 1050, 1011, 982, 874 cm^{-1}.

1f: Isolated in the yields and with the diastereomeric ratios reported in Scheme 2 in the main manuscript under the different conditions.

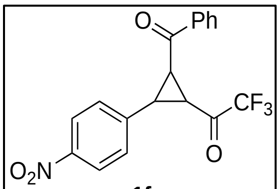

1f
A: 83% (*d.r.* = 17:**55**:28)
B: 75% (*d.r.* = 4:**87**:9)
C: 72% (*d.r.* = 18:8:**74**)

HRMS (ESI): *m/z* calculated for $C_{18}H_{11}F_3NO_4^-$: 362.0646 $[M-H]^-$; found: 362.0652 (measured from the crude reaction mixture).

1f-D1: ^{1}H-NMR (300 MHz, $CDCl_3$, 298 K): δ = 8.29 (d, J = 8.6 Hz, 2H), 7.98 (d, J = 7.5 Hz, 2H), 7.57-7.41 (m, 6H), 3.62-3.57 (m, 2H), 3.09 (dd, J^1 = 6.7 Hz, J^2 = 8.7 Hz, 1H) ppm; ^{19}F-NMR (282 MHz, $CDCl_3$, 298 K): δ = -78.09 (s, 3F) ppm.

1f-D2: ^{1}H-NMR (700 MHz, $CDCl_3$, 298 K): δ = 8.10 (d, J = 8.8 Hz, 2H), 7.94 (d, J = 7.4 Hz, 2H), 7.62 (t, J = 7.5 Hz, 1H), 7.49 (d, J = 7.8 Hz, 2H), 7.35 (d, J = 8.6 Hz, 2H), 3.89 (dd, J^1 = 4.8 Hz, J^2 = 10.1 Hz, 1H), 3.86 (dd, J^1 = 4.8 Hz, J^2 = 6.1 Hz, 1H), 3.53 (dd, J^1 = 6.1 Hz, J^2 = 10.1 Hz, 1H); ^{19}F-NMR (282 MHz, $CDCl_3$, 298 K): δ = -78.60 (s, 3F) ppm; ^{13}C-NMR (176 MHz, $CDCl_3$, 298 K): δ = 191.3 (**C**=O), 188.9 (q, J = 37.5 Hz, **C**O-CF_3), 147.6 (Ar-**C**), 140.1 (Ar-**C**), 136.6 (Ar-**C**), 134.4 (Ar-**C**), 129.8 (Ar-**C**), 129.2 (Ar-**C**), 128.5 (Ar-**C**), 123.9 (Ar-**C**), 115.7 (q, J = 289.6 Hz, **C**F_3), 38.2 (**C**H), 37.6 (**C**H), 28.3 (**C**H) ppm.

1f-D3: ^{1}H-NMR (700 MHz, $CDCl_3$, 298 K): δ = 8.21(d, J = 8.6 Hz, 2H), 8.10 (d, J = 7.3 Hz, 2H), 7.70 (t, J = 7.9 Hz, 1H), 7.58 (t, J = 7.8 Hz, 2H), 7.47 (d, J = 8.6 Hz, 2H), 4.15 (dd, J^1 = 4.9 Hz, J^2 = 6.7 Hz, 1H), 3.68 (dd, J^1 = 6.7 Hz, J^2 = 9.6 Hz, 1H), 3.53 (dd, J^1 = 4.9 Hz, J^2 = 9.6 Hz, 1H) ppm; ^{19}F-NMR (282 MHz, $CDCl_3$, 298 K): δ = -79.11 (s, 3F) ppm; ^{13}C-NMR (176 MHz, $CDCl_3$, 298 K): δ = 194.3 (**C**=O), 185.4 (q, J = 37.3 Hz, **C**O-CF_3), 147.8 (Ar-**C**), 139.6 (Ar-**C**), 136.2 (Ar-**C**), 134.6 (Ar-**C**), 130.2 (Ar-**C**), 129.3 (Ar-**C**), 128.6 (Ar-**C**), 124.0 (Ar-**C**), 115.3 (q, J = 291.1 Hz, **C**F_3), 38.5 (**C**H), 33.9 (**C**H), 30.8 (**C**H) ppm. IR (film): $\tilde{\nu}$ = 3080,

3064, 2857, 2363, 2342, 1745, 1673, 1600, 1520, 1451, 1345, 1272, 1183, 1110, 1051, 1015, 984, 892, 882 cm^{-1}.

<u>**1g**</u>: Isolated in the yields and with the diastereomeric ratios reported in Scheme 2 in the main manuscript under the different conditions.

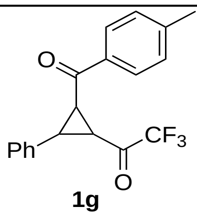

1g
A: 73% (*d.r.* = 15:10:**75**)
B: 60% (*d.r.* = 5:**77**:18)
C: 74% (*d.r.* = 11:26:**63**)

HRMS (ESI): *m/z* calculated for $C_{19}H_{16}F_3O_2^+$: 333.1097 $[M+H]^+$; found: 333.1100 (measured from the crude reaction mixture);

1g-D1 (only isolated in minor quantities coeluding with the major diastereomers, therefore only the 19F signal measured from the crude mixture is given): ^{19}F-NMR (282 MHz, $CDCl_3$, 298 K): δ = -78.16 (s, 3F) ppm.

1g-D2: ^{1}H-NMR (700 MHz, $CDCl_3$, 298 K): δ = 7.89 (d, J = 8.1 Hz, 2H), 7.23-7.18 (m, 4H), 7.15 (q, J = 6.8 Hz, 2H), 3.82 (dd, J^1 = 5.0 Hz, J^2 = 5.9 Hz, 1H), 3.79 (dd, J^1 = 5.0 Hz, J^2 = 10.1 Hz, 1H), 3.47 (dd, J^1 = 5.9 Hz, J^2 = 10.1 Hz, 1H) ppm; ^{19}F-NMR (282 MHz, $CDCl_3$, 298 K): δ = -78.61 (s, 3F) ppm; ^{13}C-NMR (176 MHz, $CDCl_3$, 298 K): δ = 191.3 (**C**=O), 189.8 (q, J = 37.1 Hz, **C**O-CF_3), 144.9 (Ar-**C**), 134.6 (Ar-**C**), 132.8 (Ar-**C**), 129.6 (Ar-**C**), 128.7 (Ar-**C**), 128.6 (Ar-**C**), 128.6 (Ar-**C**), 128.0 (Ar-**C**), 115.9 (q, J = 289.9 Hz, **C**F_3), 110.6 (Ar-**C**), 39.8 (**C**H) 38.1 (**C**H), 28.1 (**C**H), 21.9 (**C**H_3) ppm.

1g-D3: ^{1}H-NMR (700 MHz, $CDCl_3$, 298 K): δ = 8.00 (d, J = 8.1 Hz, 2H), 7.34 (dd, J^1 = 7.9 Hz, J^2 = 13.6 Hz, 4H), 7.30-7.28 (m, 3H), 4.11 (dd, J^1 = 5.0 Hz, J^2 = 6.6 Hz, 1H), 3.61 (dd, J^1 = 6.6 Hz, J^2 = 9.6 Hz, 1H), 3.44 (dd, J^1 = 5.0 Hz, J^2 = 9.6 Hz, 1H) ppm; ^{19}F-NMR (282 MHz, $CDCl_3$, 298 K): δ = -79.09 (s, 3F) ppm; ^{13}C-NMR (176 MHz, $CDCl_3$, 298 K): δ = 194.8 (**C**=O), 185.6 (q, J = 36.3 Hz, **C**O-CF_3), 145.3 (Ar-**C**), 134.1 (Ar-**C**), 132.3 (Ar-**C**), 129.8 (Ar-**C**), 128.7 (Ar-**C**), 128.7 (Ar-**C**), 128.3 (Ar-**C**), 115.5 (q, J = 290.7 Hz, **C**F_3), 40.1 (**C**H), 33.8 (**C**H), 30.7 (**C**H), 21.9 (**C**H_3) ppm. IR (film): $\tilde{\nu}$ = 3067, 3036, 2928, 2367, 2336, 1748, 1666, 1609, 1576, 1501, 1454, 1435, 1377, 1328, 1269, 1204, 1182, 1150, 1097, 1063, 1051, 1047, 1029, 987, 920, 873, 856 cm^{-1}.

<u>**1h**</u>: Isolated in the yields and with the diastereomeric ratios reported in Scheme 2 in the main manuscript under the different conditions.

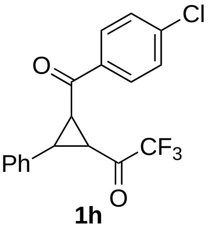

1h
A: 72% (*d.r.* = 32:**37**:31)
B: 76% (*d.r.* = 4:**83**:13)
C: 69% (*d.r.* = 18:12:**70**)

HRMS (ESI): *m/z* calculated for $C_{18}H_{11}F_3NO_4^-$: 351.0405 $[M-H]^-$; found: 351.0398 (measured from the crude reaction mixture);

1h-D1 (only isolated in minor quantities coeluding with the major diastereomers, therefore only the 19F signal measured from the crude mixture is given): ^{19}F-NMR (282 MHz, $CDCl_3$, 298 K): δ = -78.20 (s, 3F) ppm.

1h-D2: ^{1}H-NMR (700 MHz, $CDCl_3$, 298 K): δ = 7.90 (d, J = 8.6 Hz, 2H), 7.44 (d, J = 8.6 Hz, 2H), 7.24-7.20 (m, 3H), 7.15-7.13 (m, 2H), 3.83 (dd, J^1 = 4.8 Hz, J^2 = 5.8 Hz, 1H), 3.76 (dd, J^1 = 4.8 Hz, J^2 = 10.2 Hz, 1H), 3.48 (dd, J^1 = 5.8 Hz, J^2 = 10.2 Hz, 1H) ppm; ^{19}F-NMR (282 MHz, $CDCl_3$, 298 K): δ = -78.61 (s, 3F) ppm; ^{13}C-NMR (176 MHz, $CDCl_3$, 298 K): δ = 190.5 (**C**=O), 189.4 (q, J = 37.7 Hz, **C**O-CF_3), 140.3 (Ar-**C**), 135.1 (Ar-**C**), 132.3 (Ar-**C**), 129.7 (Ar-

C), 129.2 (Ar-**C**), 128.6 (Ar-**C**), 128.5 (Ar-**C**), 128.0 (Ar-**C**), 115.8 (q, J = 291.2 Hz, **C**F_3), 39.7 (**C**H), 37.7 (**C**H), 27.9 (**C**H) ppm.

1h-D3: ^{1}H-NMR (300 MHz, $CDCl_3$, 298 K): δ = 8.04 (d, J = 8.5 Hz, 2H), 7.53 (d, J = 8.5 Hz, 2H), 7.35-7.28 (m, 5H), 4.07 (dd, J^1 = 4.9 Hz, J^2 = 6.3 Hz, 1H), 3.61 (dd, J^1 = 6.3 Hz, J^2 = 9.5 Hz, 1H), 3.49 (dd, J^1 = 4.9 Hz, J^2 = 9.5 Hz, 1H) ppm; ^{19}F-NMR (282 MHz, $CDCl_3$, 298 K): δ = -79.06 (s, 3F) ppm; ^{13}C-NMR (76 MHz, $CDCl_3$, 298 K): δ = 194.2 (**C**=O), 185.4 (q, J = 36.9 Hz, **C**O-CF_3), 140.9 (Ar-**C**), 134.8 (Ar-**C**), 132.0 (Ar-**C**), 130.0 (Ar-**C**), 129.5 (Ar-**C**), 129.1 (Ar-**C**), 128.7 (Ar-**C**), 128.4 (Ar-**C**), 115.4 (q, J = 291.0 Hz, **C**F_3), 40.2 (**C**H), 34.0 (**C**H), 30.6 (**C**H) ppm. IR (film): $\tilde{\nu}$ = 3063, 3033, 2958, 2927, 2855, 2360, 2341, 1746, 1675, 1590, 1571, 1489, 1451, 1428, 1400, 1374, 1324, 1271, 1208, 1179, 1150, 1111, 1093, 1064, 1048, 1027, 1011, 983, 872, 851, 835 cm^{-1}.

<u>**1i**:</u> Isolated in the yields and with the diastereomeric ratios reported in Scheme 2 in the main manuscript under the different conditions.

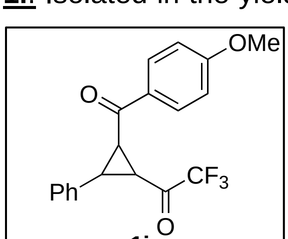

1i
A: 61% (*d.r.* = 11:**47**:42)
B: 70% (*d.r.* = 8:**63**:29)
C: 73% (*d.r.* = 13:9:**78**)

HRMS (ESI): *m/z* calculated for $C_{19}H_{16}F_3O_3^+$: 349.1046 $[M+H]^+$; found: 349.1050 (measured from the crude reaction mixture);

1i-D1 (only isolated in minor quantities coeluding with the major diastereomers, therefore only the 19F signal measured from the crude mixture is given): ^{19}F-NMR (282 MHz, $CDCl_3$, 298 K): δ = -78.14 (s, 3F) ppm.

1i-D2: ^{1}H-NMR (700 MHz, $CDCl_3$, 298 K): δ = 7.94 (d, J = 8.6 Hz, 2H), 7.39-7.33 (m, 3H), 7.24-7.20 (m, 2H), 6.94 (d, J = 8.7 Hz, 2H), 3.87 (s, 3H), 3.47 (dd, J^1 = 8.3 Hz, J^2 = 10.4 Hz, 1H), 3.45 (dd, J^1 = 5.8 Hz, J^2 = 10.4 Hz, 1H), 2.94 (dd, J^1 = 5.8 Hz, J^2 = 8.3 Hz, 1H) ppm; ^{19}F-NMR (282 MHz, $CDCl_3$, 298 K): δ = -78.14 (s, 3F) ppm; ^{13}C-NMR (176 MHz, $CDCl_3$, 298 K): δ = 191.1 (**C**=O), 186.9 (q, J = 36.5 Hz, **C**O-CF_3), 164.3 (Ar-**C**), 136.9 (Ar-**C**), 131.1 (Ar-**C**), 129.2 (Ar-**C**), 128.7 (Ar-**C**), 128.6 (Ar-**C**), 127.9 (Ar-**C**), 126.7 (Ar-**C**), 115.7 (q, J = 295.3 Hz, **C**F_3), 114.2 (Ar-**C**), 55.7 (**C**H_3) 39.5 (**C**H), 32.3 (**C**H), 31.2 (**C**H) ppm.

1i-D3: ^{1}H-NMR (700 MHz, $CDCl_3$, 298 K): δ 8.10 (d, J = 8.7 Hz, 2H), 7.33 (t, J = 7.3 Hz, 2H), 7.29-7.27 (m, 3H), 7.02 (d, J = 8.6 Hz, 2H), 4.01 (dd, J^1 = 4.1 Hz, J^2 = 7.1 Hz, 1H), 3.91 (s, 3H), 3.61 (dd, J^1 = 7.1 Hz, J^2 = 9.0 Hz, 1H), 3.48 (dd, J^1 = 4.1 Hz, J^2 = 9.0 Hz, 1H) ppm; ^{19}F-NMR (282 MHz, $CDCl_3$, 298 K): δ = -79.09 (s, 3F) ppm; ^{13}C-NMR (176 MHz, $CDCl_3$, 298 K): δ = 193.5 (**C**=O), 185.7 (q, J = 36.8 Hz, **C**O-CF_3), 164.5 (Ar-**C**), 132.4 (Ar-**C**), 131.0 (Ar-**C**), 129.6 (Ar-**C**), 129.2 (Ar-**C**), 128.7 (Ar-**C**), 128.2 (Ar-**C**), 115.4 (q, J = 290.8 Hz, **C**F_3), 114.3 (Ar-**C**), 55.8 (**C**H_3), 40.0 (**C**H), 33.7 (**C**H), 30.6 (**C**H) ppm. IR (film): $\tilde{\nu}$ = 3069, 2945, 2847, 2363, 2326, 1740, 1670, 1597, 1589, 1510, 1464, 1435, 1383, 1331, 1267, 1209, 1175, 1145, 1096, 1065, 1030, 1003, 978, 840 cm^{-1}.

<u>**1j**:</u> Isolated in the yields and with the diastereomeric ratios reported in Scheme 2 in the main manuscript under the different conditions.

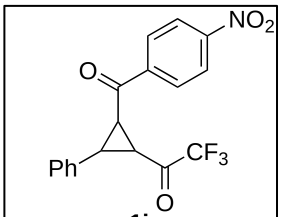

1j
A: 70% (*d.r.* = **50**:20:30)
B: 8% (*d.r.* = 17:**76**:7)
C: 61% (*d.r.* = 36:10:**54**)

HRMS (ESI): *m/z* calculated for $C_{18}H_{11}F_3NO_4^-$: 362.0646 $[M-H]^-$; found: 362.0668 (measured from the crude reaction mixture);

1j-D1: ^{1}H-NMR (300 MHz, $CDCl_3$, 298 K): δ = 8.35-8.30 (m, 2H), 8.13 (d, J = 8.2 Hz, 2H), 7.44-7.38 (m, 3H), 7.27-7.23 (m, 3H), 3.57-3.45 (m, 2H), 3.01 (dd, J^1 = 6.2 Hz, J^2 = 9.1 Hz, 1H) ppm; ^{19}F-NMR (282 MHz, $CDCl_3$, 298 K): δ = -78.22 (s, 3F) ppm.

1j-D2: ^{1}H-NMR (700 MHz, $CDCl_3$, 298 K): δ = 8.32 (d, J = 8.7 Hz, 2H), 7.10 (d, J = 8.8 Hz, 2H), 7.41 (t, J = 7.4 Hz, 1H), 7.24 (d, J = 7.5 Hz, 2H), 7.14 (d, J = 6.8 Hz, 2H), 3.82 (dd, J^1 = 5.0 Hz, J^2 = 5.9 Hz, 1H), 3.79 (dd, J^1 = 5.0 Hz, J^2 = 10.1 Hz, 1H), 3.47 (dd, J^1 = 5.9 Hz, J^2 = 10.1 Hz, 1H) ppm; ^{19}F-NMR (282 MHz, $CDCl_3$, 298 K): δ = -78.61 (s, 3F) ppm; ^{13}C-NMR (76 MHz, $CDCl_3$, 298 K): δ = 190.6 (**C**=O), 189.2 (q, J = 38.1 Hz, **C**O-CF_3), 141.1 (Ar-**C**), 131.9 (Ar-**C**), 129.6 (Ar-**C**), 129.4 (Ar-**C**), 128.8 (Ar-**C**), 128.6 (Ar-**C**), 128.4 (Ar-**C**), 124.4 (Ar-**C**), 40.1 (**C**H), 38.0 (**C**H), 28.1 (**C**H) ppm.

1j-D3: ^{1}H-NMR (700 MHz, $CDCl_3$, 298 K): δ = 8.40 (d, J = 8.6 Hz, 2H), 8.26 (d, J = 8.7 Hz, 2H), 7.36 (t, J = 7.5 Hz, 2H), 7.32 (t, J = 7.10 Hz, 1H), 7.28 (d, J = 7.5 Hz, 2H), 4.11 (dd, J^1 = 4.8 Hz, J^2 = 6.8 Hz, 1H), 3.65 (dd, J^1 = 6.8 Hz, J^2 = 9.7 Hz, 1H), 3.55 (dd, J^1 = 4.8 Hz, J^2 = 9.7 Hz, 1H) ppm; ^{19}F-NMR (282 MHz, $CDCl_3$, 298 K): δ = -79.02 (s, 3F) ppm; ^{13}C-NMR (176 MHz, $CDCl_3$, 298 K): δ = 194.2 (**C**=O), 185.1 (q, J = 36.9 Hz, **C**O-CF_3), 151.0 (Ar-**C**), 140.8 (Ar-**C**), 131.5 (Ar-**C**), 129.6 (Ar-**C**), 129.0 (Ar-**C**), 128.9 (Ar-**C**), 128.6 (Ar-**C**), 124.4 (Ar-**C**), 115.4 (q, J = 290.8 Hz, **C**F_3), 40.5 (**C**H), 34.4 (**C**H), 30.9 (**C**H) ppm. IR (film): $\bar{\nu}$ = 3073, 3046, 2951, 2365, 2330, 1744, 1674, 1587, 1487, 1437, 1398, 1379, 1323, 1275, 1207, 1181, 1148, 1096, 1030, 1010, 1004, 957, 920, 852 cm^{-1}.

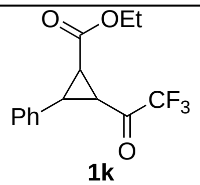

1k
A: 32% (single isomer)
B: no rxt
C: <5%

1k: Isolated in the yields and with the diastereomeric ratios reported in Scheme 2 in the main manuscript under the different conditions.

1k: ^{1}H-NMR (700 MHz, $CDCl_3$, 298 K): δ = 7.32-7.27 (m, 3H), 7.25-6.24 (m, 2H), 4.02-3.95 (m, 2H), 3.44 (dd, J^1 = 4.6 Hz, J^2 = 5.8 Hz, 1H), 3.22 (dd, J^1 = 5.8 Hz, J^2 = 10.1 Hz, 1H), 2.89 (dd, J^1 = 4.6 Hz, J^2 = 10.1 Hz, 1H), 1.04 (t, J = 7.1 Hz, 3 H) ppm; ^{19}F-NMR (282 MHz, $CDCl_3$, 298 K): δ = -78.67 (s, 3F) ppm; ^{13}C-NMR (176 MHz, $CDCl_3$, 298 K): δ = 189.0 (q, J = 37.4 Hz, F_3C-**C**=O), 167.2 (**C**=O), 133.2 (Ar-**C**), 129.1 (Ar-**C**), 128.9 (Ar-**C**), 128.6 (Ar-**C**), 128.1 (Ar-**C**), 126.6 (Ar-**C**), 115.9 (q, J = 288.2 Hz, **C**F_3), 61.6 (**C**H_2-O), 37.1 (**C**H), 33.1 (**C**H), 28.1 (**C**H), 14.1 (**C**H_3-CH_2-O) ppm.

HRMS (ESI): *m/z* calculated for $C_{14}H_{14}F_3O_3^+$: 287.0895 $[M+H]^+$; found: 287.0893.

2.3 Asymmetric Cyclopropanation

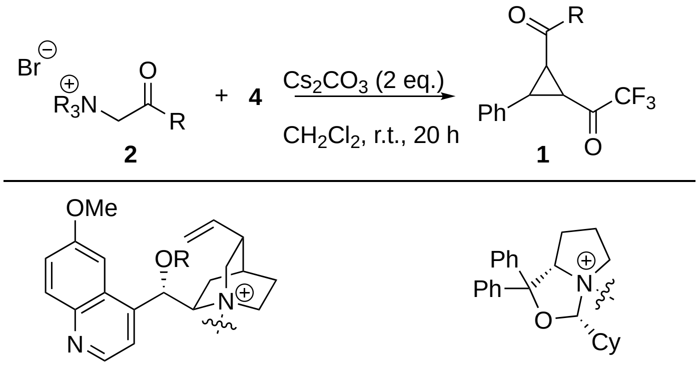

A1 (R = H): 39%
(*d.r.* = 21:12:67; *ee* = 35:nd:70)

B: 44%
(*d.r.* = 6:54:40; *ee* = nd:24:48)

A2 (R = Me): 37%
(*d.r.* = 18:41:41; *ee* = nd:35:57)

The reactions were carried out as described above (Cond. A) with the chiral ammonium salts that were synthesized as described recently.[2] Determination of the enantiomeric excess was carried out by HPLC analysis using a chiral stationary phase (Chiralcel AD-H (250 x 4.6 mm, 10 µm)) with *n*-hexane/*i*-PrOH (98:2) as an eluent at 10 °C. Retention times: D1: t_{major} = 16.1 min, t_{minor} = 20.3 min; D2: t_{major} = 14.6 min, t_{minor} = 16.5 min; D3: t_{major} = 16.1 min, t_{minor} = 19.9 min.

4 WIN-195-02_100_1_flo5

Sample Name:	WIN-195-02_100_1_flo5	*Injection Volume:*	20,0
Vial Number:	BC8	*Channel:*	UV_VIS_1
Sample Type:	unknown	*Wavelength:*	220
Control Program:	AD_H_60Min_100A_flow0_5	*Bandwidth:*	4
Quantif. Method:	default	*Temperature/Column:*	10
Recording Time:	14.8.2017 14:43	*Flow ml/min:*	0,500
Run Time (min):	27,68	*Sample Amount:*	1,0000

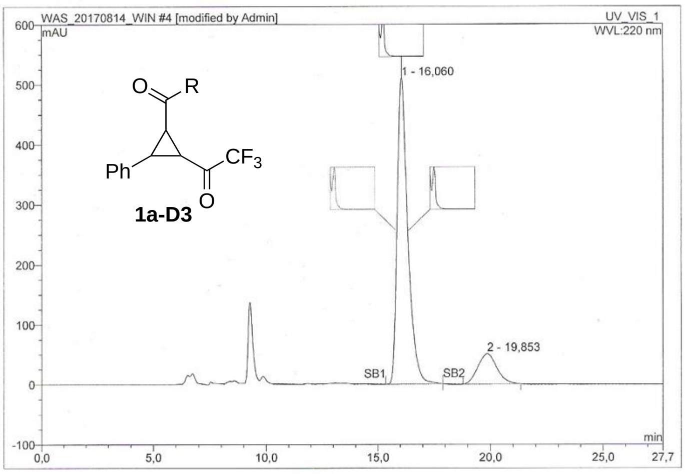

No.	Ret.Time min	Peak Name	Height mAU	Area mAU*min	Rel.Area %	Amount	Type
1	16,06	n.a.	510,080	280,979	85,09	n.a.	BMB
2	19,85	n.a.	50,727	49,232	14,91	n.a.	BMB
Total:			560,807	330,211	100,00	0,000	

4. Copies of NMR-Spectra of new Compounds

In most cases, especially for D1 and D2 the diastereomers could not totally be separated and the NMR spectra show mixtures thereof (including the hydrate **5**). In the ^{1}H NMR only the signals for the indicated major diastereomer are integrated.

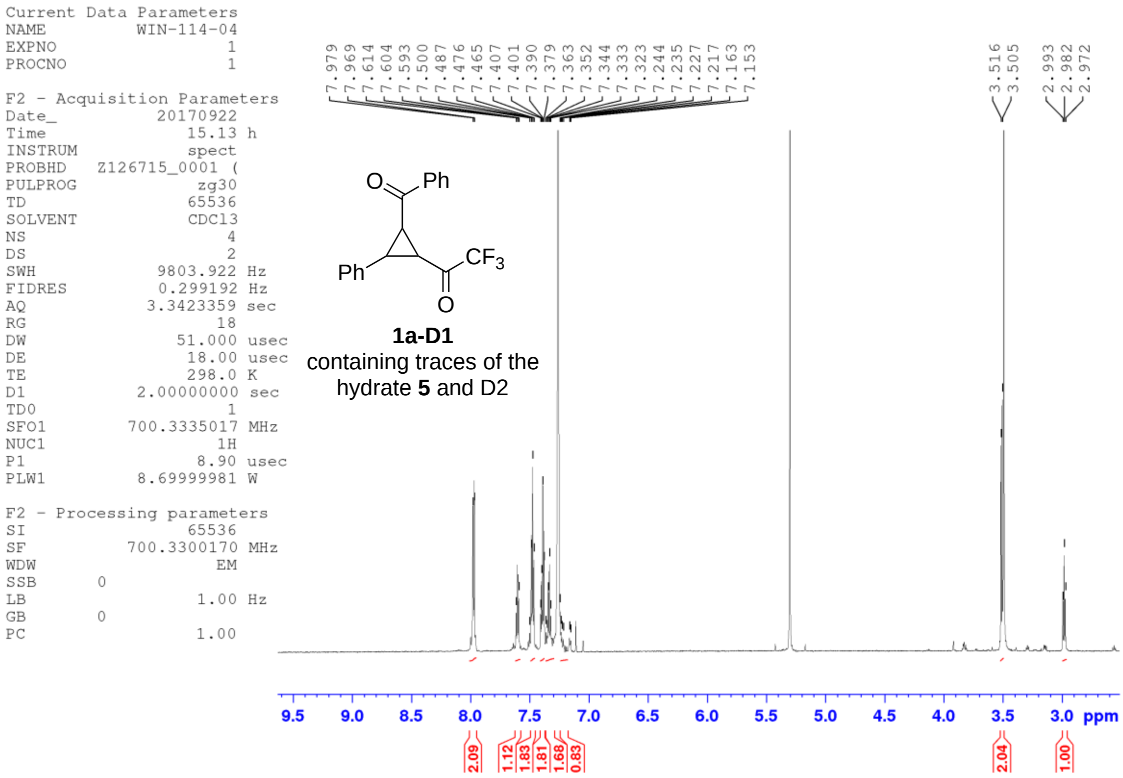

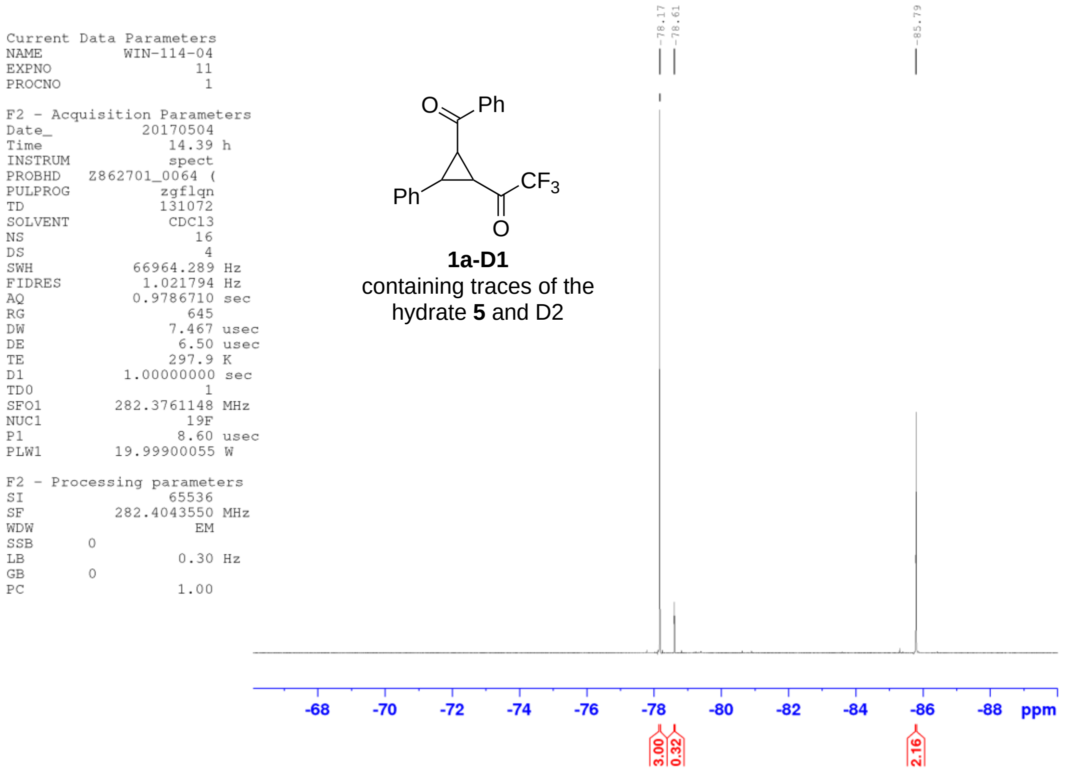
Current Data Parameters
NAME WIN-114-04
EXPNO 11
PROCNO 1
F2 - Acquisition Parameters
Date_ 20170504
Time 14.39 h
INSTRUM spect
PROBHD Z862701_0064 (
PULPROG zgflqn
TD 131072
SOLVENT CDCl3
NS 16
DS 4
SWH 66964.289 Hz
FIDRES 1.021794 Hz
AQ 0.9786710 sec
RG 645
DW 7.467 usec
DE 6.50 usec
TE 297.9 K
D1 1.00000000 sec
TD0 1
SFO1 282.3761148 MHz
NUC1 19F
P1 8.60 usec
PLW1 19.99900055 W
F2 - Processing parameters
SI 65536
SF 282.4043550 MHz
WDW EM
SSB 0
LB 0.30 Hz
GB 0
PC 1.00
-78.17
-78.61
-85.79
O
Ph
Ph
CF3
O
1a-D1
containing traces of the
hydrate 5 and D2
-68
-70
-72
-74
-76
-78
-80
-82
-84
-86
-88
ppm
3.00
0.32
2.16

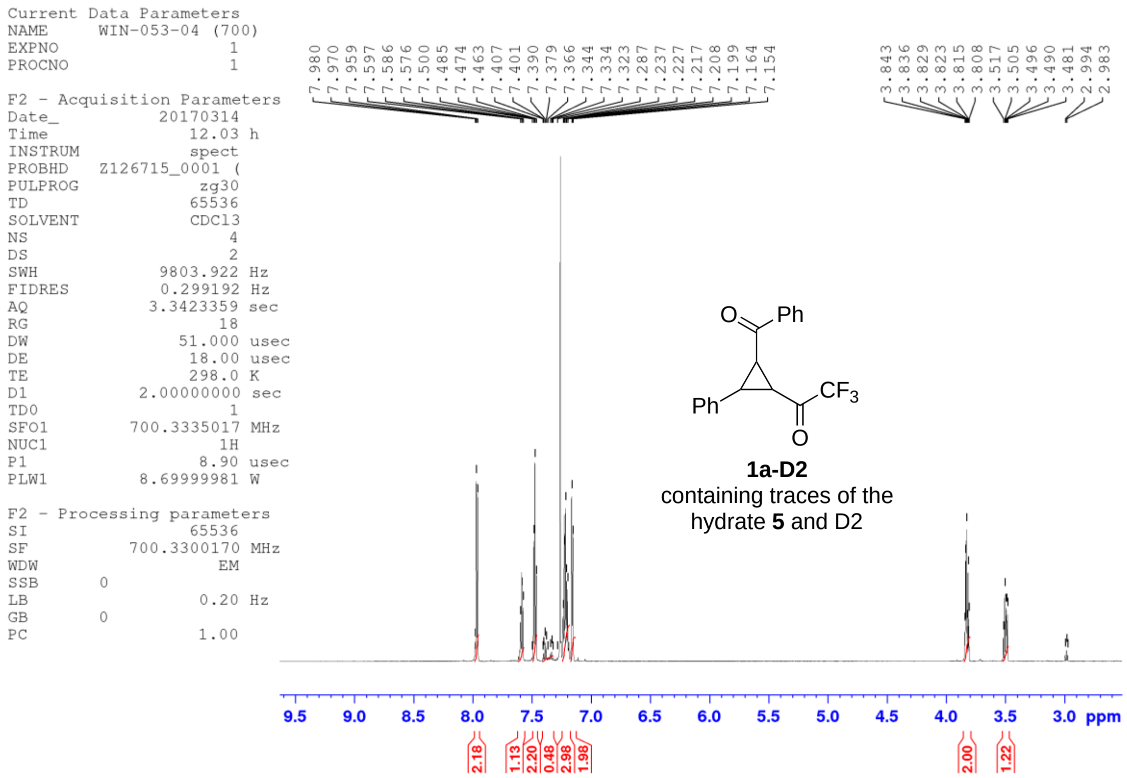
Current Data Parameters
NAME WIN-053-04 (700)
EXPNO 1
PROCNO 1
F2 - Acquisition Parameters
Date_ 20170314
Time 12.03 h
INSTRUM spect
PROBHD Z126715_0001 (
PULPROG zg30
TD 65536
SOLVENT CDCl3
NS 4
DS 2
SWH 9803.922 Hz
FIDRES 0.299192 Hz
AQ 3.3423359 sec
RG 18
DW 51.000 usec
DE 18.00 usec
TE 298.0 K
D1 2.00000000 sec
TD0 1
SFO1 700.3335017 MHz
NUC1 1H
P1 8.90 usec
PLW1 8.69999981 W
F2 - Processing parameters
SI 65536
SF 700.3300170 MHz
WDW EM
SSB 0
LB 0.20 Hz
GB 0
PC 1.00
7.980
7.970
7.959
7.597
7.586
7.576
7.500
7.485
7.474
7.463
7.407
7.401
7.390
7.379
7.366
7.344
7.334
7.323
7.287
7.237
7.227
7.217
7.208
7.199
7.164
7.154
3.843
3.836
3.829
3.823
3.815
3.808
3.517
3.505
3.496
3.490
3.481
2.994
2.983
O
Ph
Ph
CF3
O
1a-D2
containing traces of the
hydrate 5 and D2
9.5
9.0
8.5
8.0
7.5
7.0
6.5
6.0
5.5
5.0
4.5
4.0
3.5
3.0
ppm
2.18
1.13
2.20
0.48
2.98
1.98
2.00
1.22

Current Data Parameters
NAME WIN-053-04 (700)
EXPNO 2
PROCNO 1

F2 - Acquisition Parameters
Date_ 20170314
Time 15.28 h
INSTRUM spect
PROBHD Z126715_0001 (
PULPROG zgpg30
TD 65536
SOLVENT CDCl3
NS 4096
DS 4
SWH 40760.871 Hz
FIDRES 1.243923 Hz
AQ 0.8039083 sec
RG 2050
DW 12.267 usec
DE 18.00 usec
TE 298.0 K
D1 2.00000000 sec
D11 0.03000000 sec
TD0 1
SFO1 176.1183703 MHz
NUC1 13C
P1 12.00 usec
PLW1 129.00000000 W
SFO2 700.3328013 MHz
NUC2 1H
CPDPRG[2 waltz16
PCPD2 65.00 usec
PLW2 8.69999981 W
PLW12 0.16311000 W
PLW13 0.08213100 W

F2 - Processing parameters

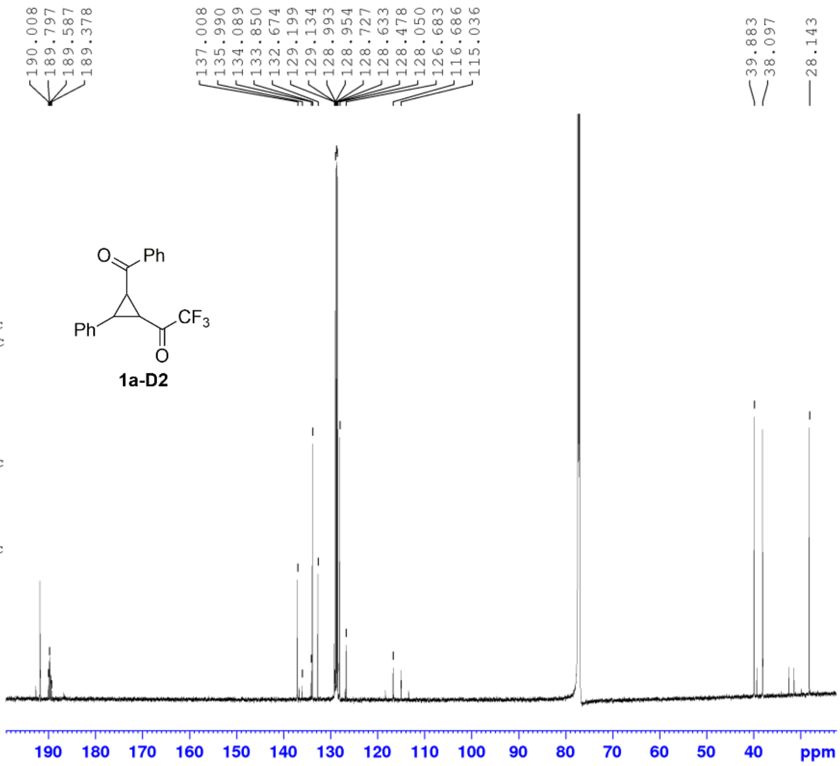

Current Data Parameters
NAME WIN-053-04
EXPNO 11
PROCNO 1

F2 - Acquisition Parameters
Date_ 20170309
Time 16.03 h
INSTRUM spect
PROBHD Z862701_0064 (
PULPROG zgflqn
TD 131072
SOLVENT CDCl3
NS 16
DS 4
SWH 66964.289 Hz
FIDRES 1.021794 Hz
AQ 0.9786710 sec
RG 645
DW 7.467 usec
DE 6.50 usec
TE 298.1 K
D1 1.00000000 sec
TD0 1
SFO1 282.3761148 MHz
NUC1 19F
P1 8.60 usec
PLW1 19.99900055 W

F2 - Processing parameters
SI 65536
SF 282.4043550 MHz
WDW EM
SSB 0
LB 0.30 Hz
GB 0
PC 1.00

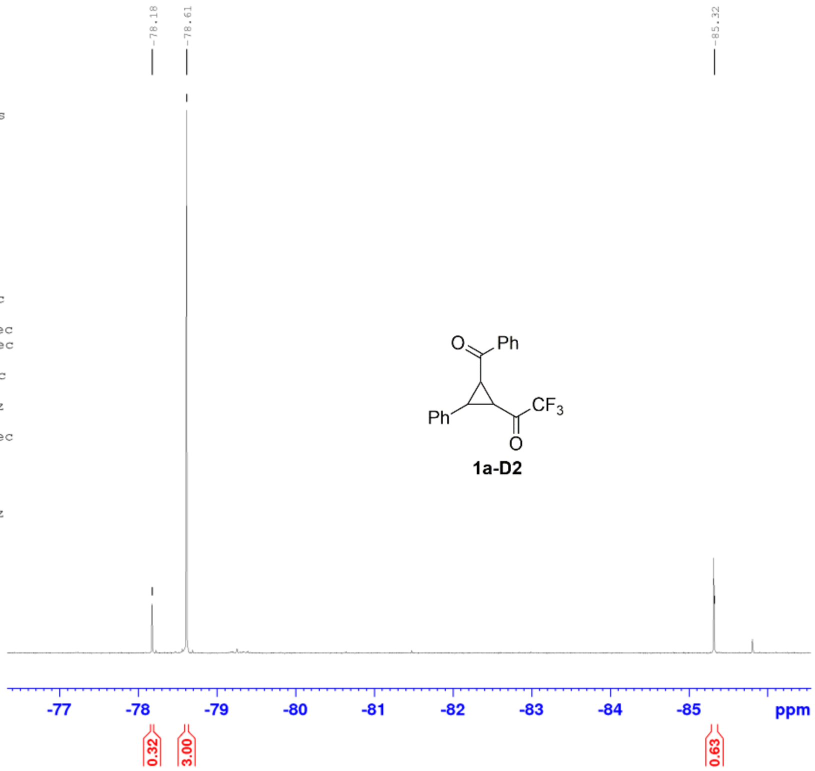

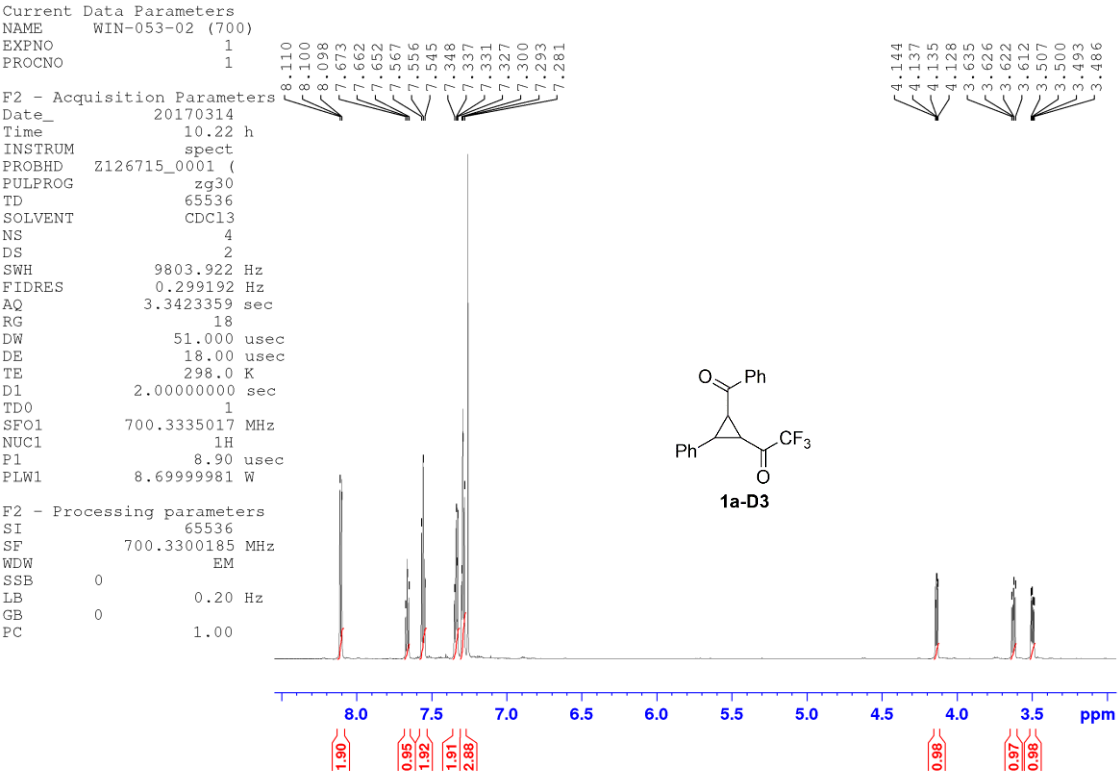
Current Data Parameters
NAME WIN-053-02 (700)
EXPNO 1
PROCNO 1
F2 - Acquisition Parameters
Date_ 20170314
Time 10.22 h
INSTRUM spect
PROBHD Z126715_0001 (
PULPROG zg30
TD 65536
SOLVENT CDCl3
NS 4
DS 2
SWH 9803.922 Hz
FIDRES 0.299192 Hz
AQ 3.3423359 sec
RG 18
DW 51.000 usec
DE 18.00 usec
TE 298.0 K
D1 2.00000000 sec
TD0 1
SFO1 700.3335017 MHz
NUC1 1H
P1 8.90 usec
PLW1 8.69999981 W
F2 - Processing parameters
SI 65536
SF 700.3300185 MHz
WDW EM
SSB 0
LB 0.20 Hz
GB 0
PC 1.00
8.110 8.100 8.098 7.673 7.662 7.652 7.567 7.556 7.545 7.348 7.337 7.331 7.327 7.300 7.293 7.281
4.144 4.137 4.135 4.128 3.635 3.626 3.622 3.612 3.507 3.500 3.493 3.486
O Ph
Ph
CF3
O
1a-D3
8.0 7.5 7.0 6.5 6.0 5.5 5.0 4.5 4.0 3.5 ppm
1.90 0.95 1.92 1.91 2.88 0.98 0.97 0.98

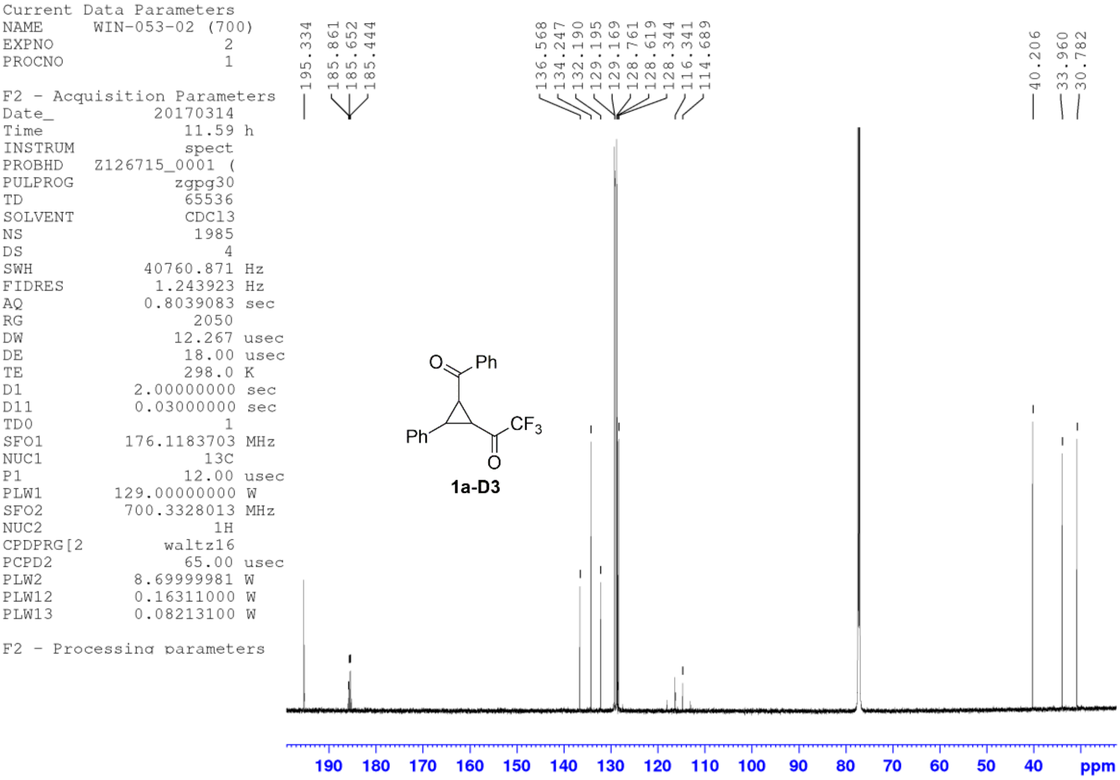
Current Data Parameters
NAME WIN-053-02 (700)
EXPNO 2
PROCNO 1
F2 - Acquisition Parameters
Date_ 20170314
Time 11.59 h
INSTRUM spect
PROBHD Z126715_0001 (
PULPROG zgpg30
TD 65536
SOLVENT CDCl3
NS 1985
DS 4
SWH 40760.871 Hz
FIDRES 1.243923 Hz
AQ 0.8039083 sec
RG 2050
DW 12.267 usec
DE 18.00 usec
TE 298.0 K
D1 2.00000000 sec
D11 0.03000000 sec
TD0 1
SFO1 176.1183703 MHz
NUC1 13C
P1 12.00 usec
PLW1 129.00000000 W
SFO2 700.3328013 MHz
NUC2 1H
CPDPRG[2 waltz16
PCPD2 65.00 usec
PLW2 8.69999981 W
PLW12 0.16311000 W
PLW13 0.08213100 W
F2 - Processing parameters
195.334 185.861 185.652 185.444
136.568 134.247 132.190 129.195 129.169 128.761 128.619 128.344 116.341 114.689
40.206 33.960 30.782
O Ph
Ph
CF3
O
1a-D3
190 180 170 160 150 140 130 120 110 100 90 80 70 60 50 40 ppm

```
Current Data Parameters
NAME           WIN-053-02
EXPNO                  11
PROCNO                  1

F2 - Acquisition Parameters
Date_            20170309
Time                15.51 h
INSTRUM             spect
PROBHD   Z862701_0064 (
PULPROG           zgflqn
TD                 131072
SOLVENT             CDCl3
NS                     16
DS                      4
SWH             66964.289 Hz
FIDRES           1.021794 Hz
AQ              0.9786710 sec
RG                    645
DW                  7.467 usec
DE                   6.50 usec
TE                  298.1 K
D1             1.00000000 sec
TD0                     1
SFO1          282.3761148 MHz
NUC1                  19F
P1                   8.60 usec
PLW1          19.99900055 W

F2 - Processing parameters
SI                  65536
SF            282.4043550 MHz
WDW                    EM
SSB      0
LB                   0.30 Hz
GB       0
PC                   1.00
```

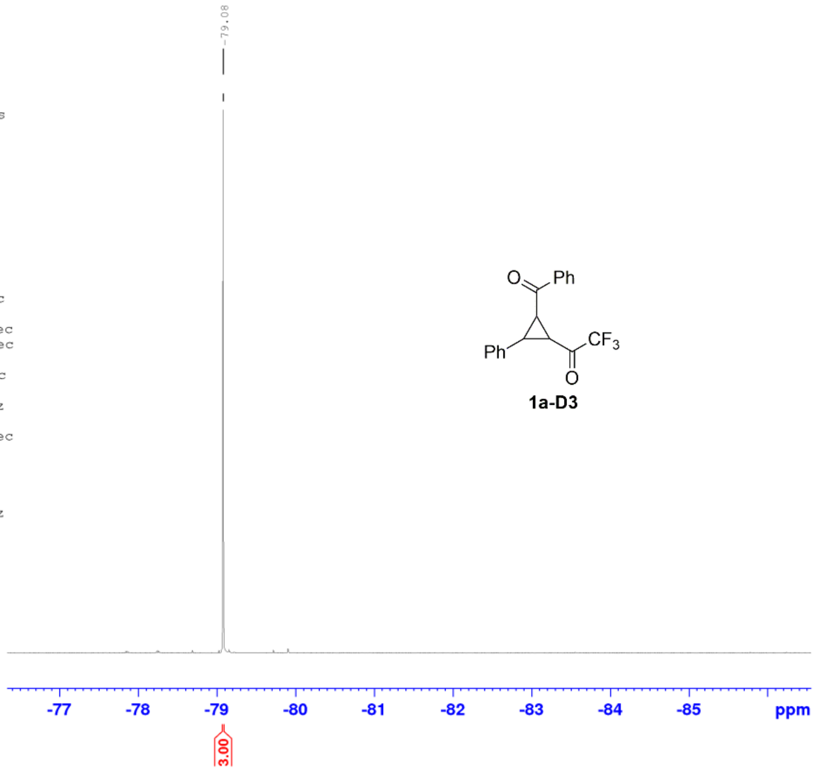


```
Current Data Parameters
NAME      WIN-133-03_700er
EXPNO                    1
PROCNO                   1

F2 - Acquisition Parameters
Date_             20170906
Time                 12.23 h
INSTRUM              spect
PROBHD    Z126715_0001 (
PULPROG               zg30
TD                   65536
SOLVENT              CDCl3
NS                       8
DS                       2
SWH               9803.922 Hz
FIDRES            0.299192 Hz
AQ               3.3423359 sec
RG                      16
DW                  51.000 usec
DE                   18.00 usec
TE                   298.0 K
D1              2.00000000 sec
TD0                      1
SFO1           700.3335017 MHz
NUC1                    1H
P1                    8.90 usec
PLW1            8.69999981 W

F2 - Processing parameters
SI                   65536
SF             700.3300170 MHz
WDW                     EM
SSB       0
LB                    0.20 Hz
GB        0
PC                    1.00
```

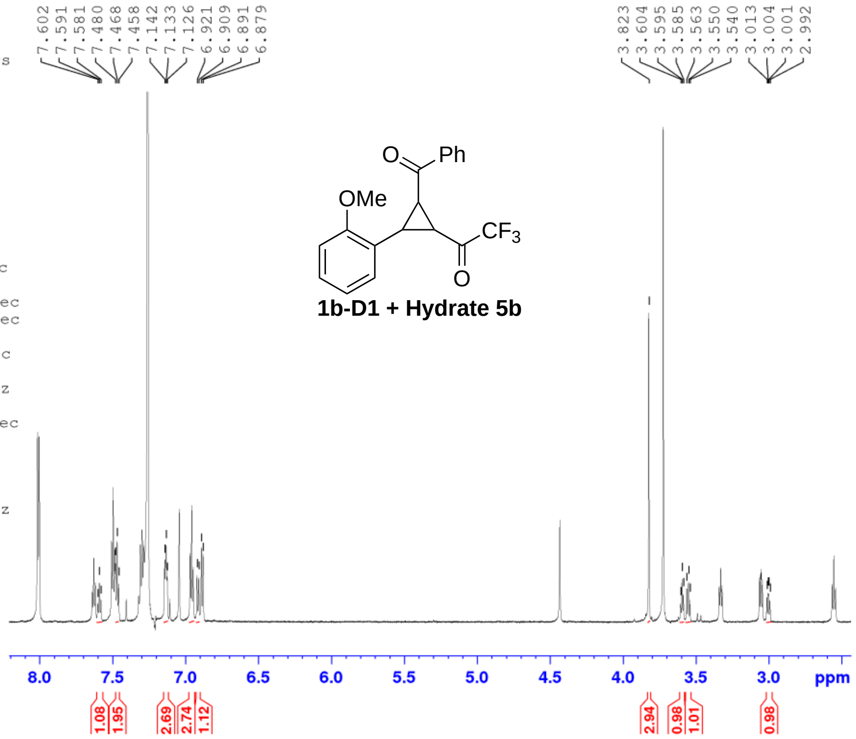

```
Current Data Parameters
NAME          WIN-133-03
EXPNO                 25
PROCNO                 1

F2 - Acquisition Parameters
Date_           20170621
Time                5.26 h
INSTRUM            spect
PROBHD   Z862701_0064 (
PULPROG          zgpg30
TD                 65536
SOLVENT            CDCl3
NS                  2048
DS                     4
SWH            18028.846 Hz
FIDRES          0.550197 Hz
AQ             1.8175317 sec
RG                  2050
DW                27.733 usec
DE                 27.73 usec
TE                 298.0 K
D1            2.00000000 sec
D11           0.03000000 sec
TD0                    1
SFO1          75.4752949 MHz
NUC1                 13C
P1                  7.75 usec
PLW1         50.00000000 W
SFO2         300.1312005 MHz
NUC2                  1H
CPDPRG[2         waltz16
PCPD2              90.00 usec
PLW2         20.00000000 W
PLW12         0.16806000 W
PLW13         0.08453100 W

F2 - Processing parameters
```

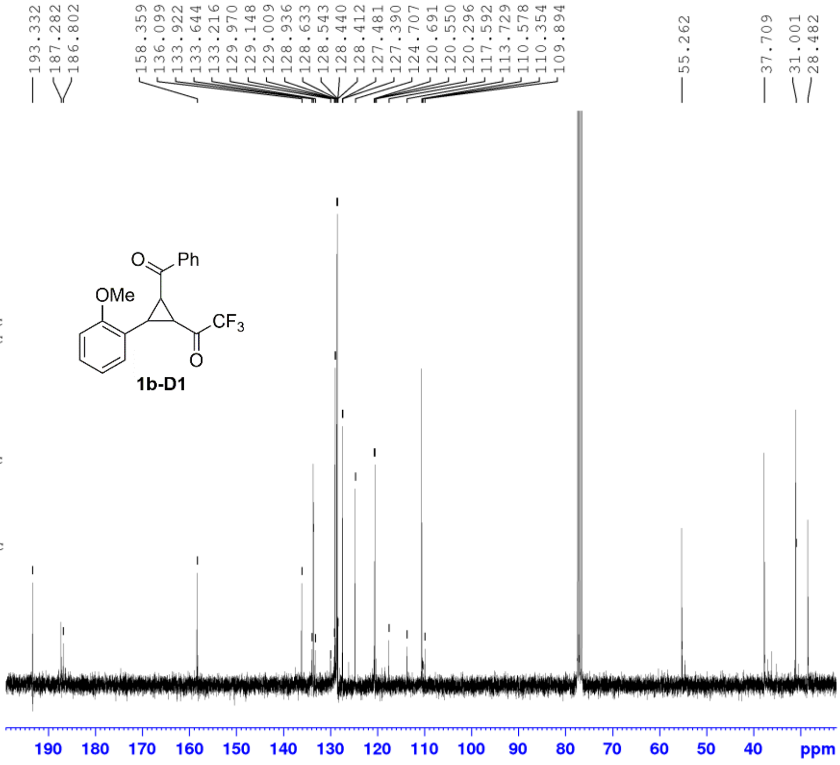


```
Current Data Parameters
NAME          WIN-133-03
EXPNO                 11
PROCNO                 1

F2 - Acquisition Parameters
Date_           20170620
Time               14.09 h
INSTRUM            spect
PROBHD   Z862701_0064 (
PULPROG          zgflqn
TD                131072
SOLVENT            CDCl3
NS                    16
DS                     4
SWH            66964.289 Hz
FIDRES          1.021794 Hz
AQ             0.9786710 sec
RG                   724
DW                 7.467 usec
DE                  6.50 usec
TE                 298.0 K
D1            1.00000000 sec
TD0                    1
SFO1         282.3761148 MHz
NUC1                 19F
P1                  8.60 usec
PLW1         19.99900055 W

F2 - Processing parameters
SI                 65536
SF           282.4043550 MHz
WDW                   EM
SSB      0
LB                  0.30 Hz
GB       0
PC                  1.00
```

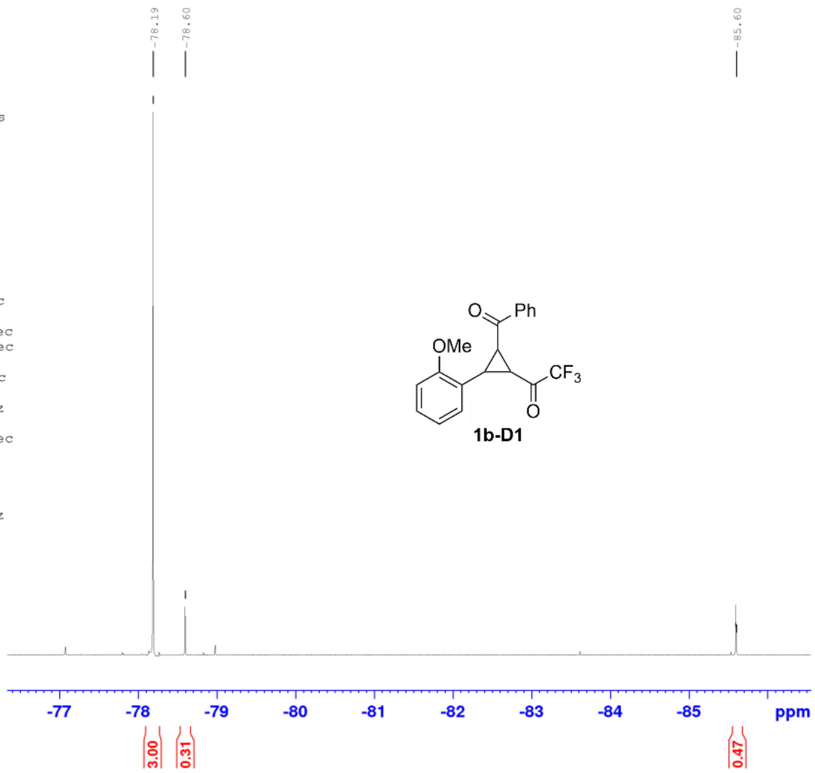

```
Current Data Parameters
NAME      WIN-133-03_700er
EXPNO                    1
PROCNO                   1

F2 - Acquisition Parameters
Date_             20170906
Time                 12.23 h
INSTRUM              spect
PROBHD    Z126715_0001 (
PULPROG               zg30
TD                   65536
SOLVENT              CDCl3
NS                       8
DS                       2
SWH               9803.922 Hz
FIDRES            0.299192 Hz
AQ               3.3423359 sec
RG                      16
DW                  51.000 usec
DE                   18.00 usec
TE                   298.0 K
D1              2.00000000 sec
TD0                      1
SFO1           700.3335017 MHz
NUC1                    1H
P1                    8.90 usec
PLW1            8.69999981 W

F2 - Processing parameters
SI                   65536
SF             700.3300170 MHz
WDW                     EM
SSB      0
LB                    0.20 Hz
GB       0
PC                    1.00
```

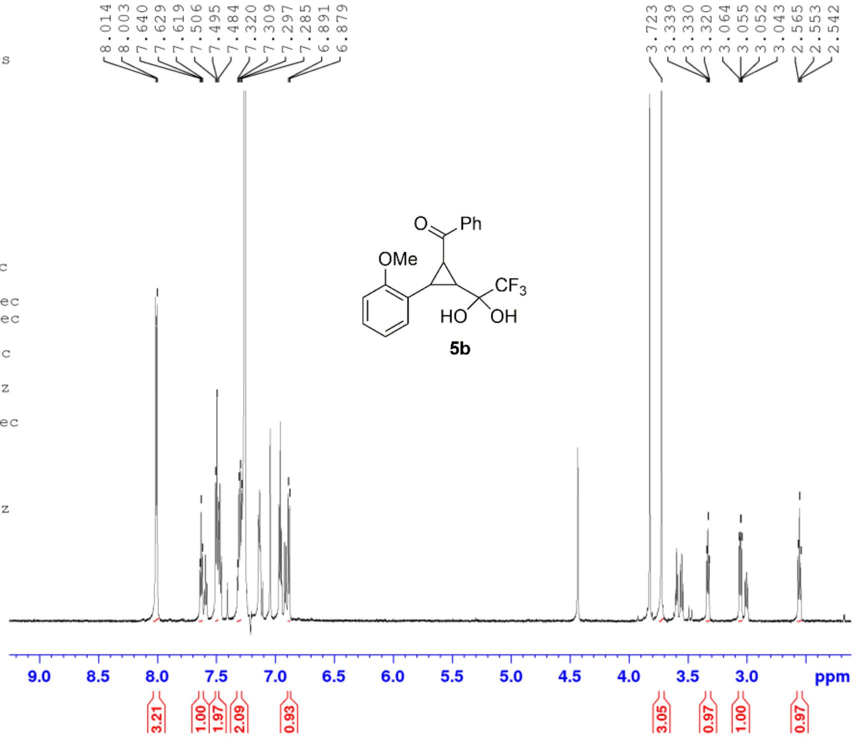


```
Current Data Parameters
NAME           WIN-134-03
EXPNO                   10
PROCNO                   1

F2 - Acquisition Parameters
Date_             20170621
Time                 14.14 h
INSTRUM              spect
PROBHD    Z862701_0064 (
PULPROG               zg30
TD                   65536
SOLVENT              CDCl3
NS                      16
DS                       2
SWH               6009.615 Hz
FIDRES            0.183399 Hz
AQ               5.4525952 sec
RG                     456
DW                  83.200 usec
DE                    6.50 usec
TE                   298.0 K
D1              1.00000000 sec
TD0                      1
SFO1           300.1318533 MHz
NUC1                    1H
P1                    8.25 usec
PLW1           20.00000000 W

F2 - Processing parameters
SI                   65536
SF             300.1300082 MHz
WDW                     EM
SSB      0
LB                    0.20 Hz
GB       0
PC                    1.00
```

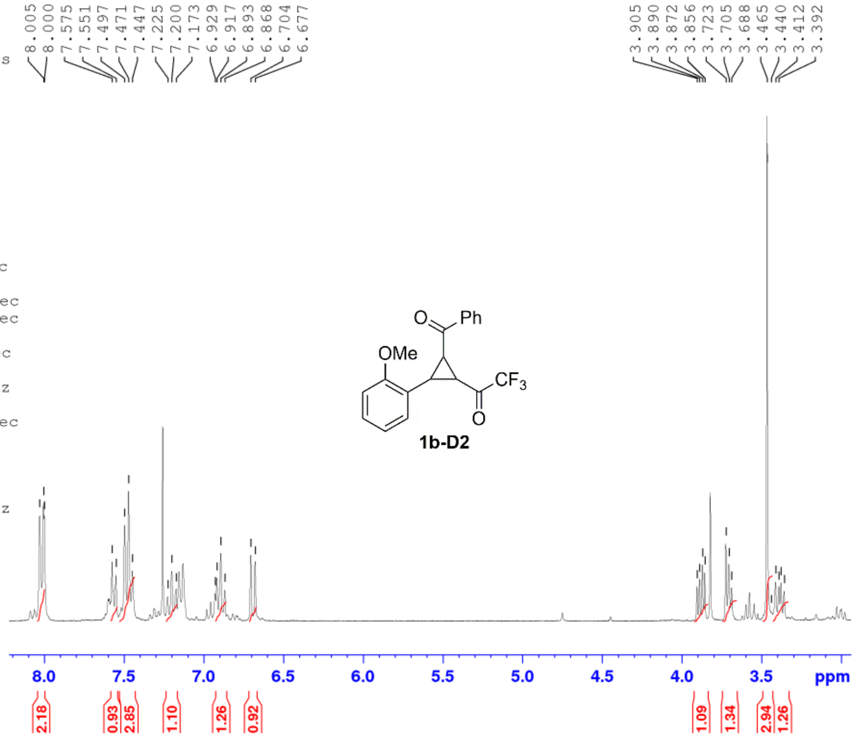

Current Data Parameters
NAME WIN-134-03
EXPNO 20
PROCNO 1

F2 - Acquisition Parameters
Date_ 20170621
Time 21.16 h
INSTRUM spect
PROBHD Z862701_0064 (
PULPROG zgpg30
TD 65536
SOLVENT CDCl3
NS 2048
DS 4
SWH 18028.846 Hz
FIDRES 0.550197 Hz
AQ 1.8175317 sec
RG 2050
DW 27.733 usec
DE 27.73 usec
TE 298.0 K
D1 2.00000000 sec
D11 0.03000000 sec
TD0 1
SFO1 75.4752949 MHz
NUC1 13C
P1 7.75 usec
PLW1 50.00000000 W
SFO2 300.1312005 MHz
NUC2 1H
CPDPRG[2 waltz16
PCPD2 90.00 usec
PLW2 20.00000000 W
PLW12 0.16806000 W
PLW13 0.08453100 W

F2 - Processing parameters

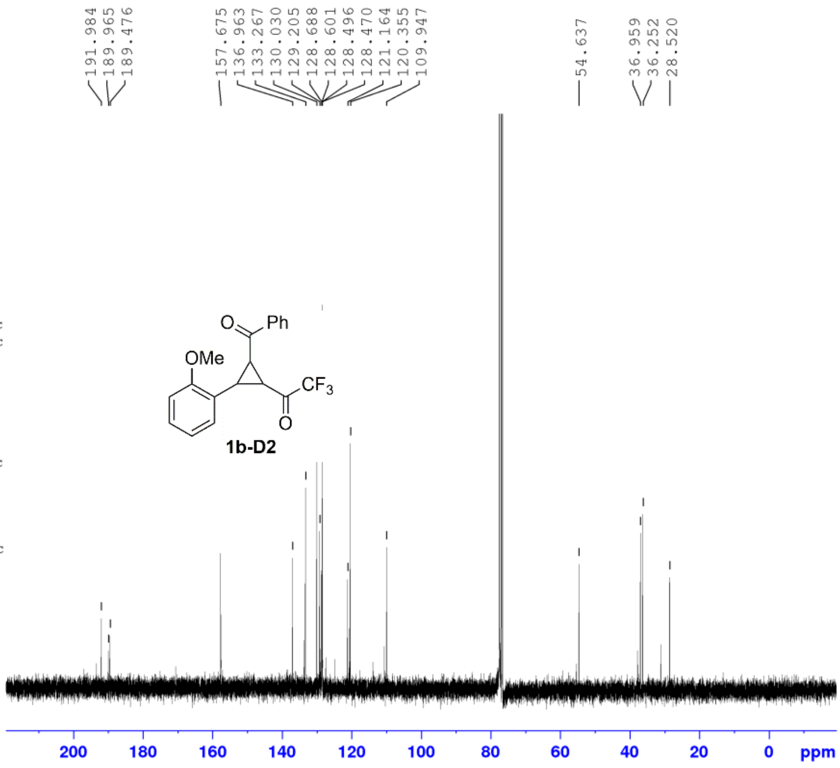

Current Data Parameters
NAME WIN-134-03
EXPNO 11
PROCNO 1

F2 - Acquisition Parameters
Date_ 20170621
Time 14.15 h
INSTRUM spect
PROBHD Z862701_0064 (
PULPROG zgflqn
TD 131072
SOLVENT CDCl3
NS 16
DS 4
SWH 66964.289 Hz
FIDRES 1.021794 Hz
AQ 0.9786710 sec
RG 645
DW 7.467 usec
DE 6.50 usec
TE 298.0 K
D1 1.00000000 sec
TD0 1
SFO1 282.3761148 MHz
NUC1 19F
P1 8.60 usec
PLW1 19.99900055 W

F2 - Processing parameters
SI 65536
SF 282.4043550 MHz
WDW EM
SSB 0
LB 0.30 Hz
GB 0
PC 1.00

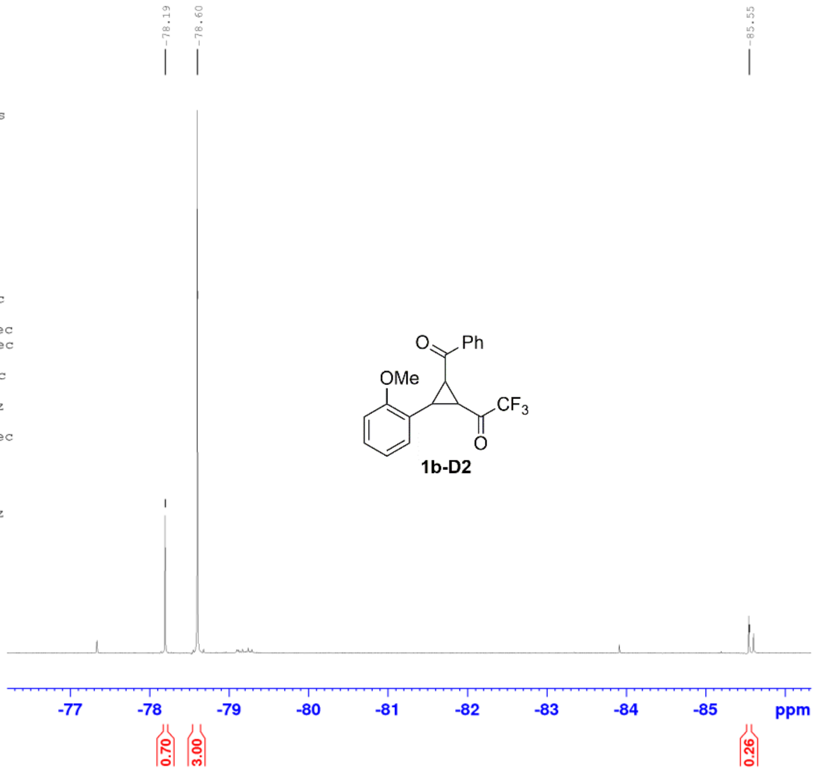

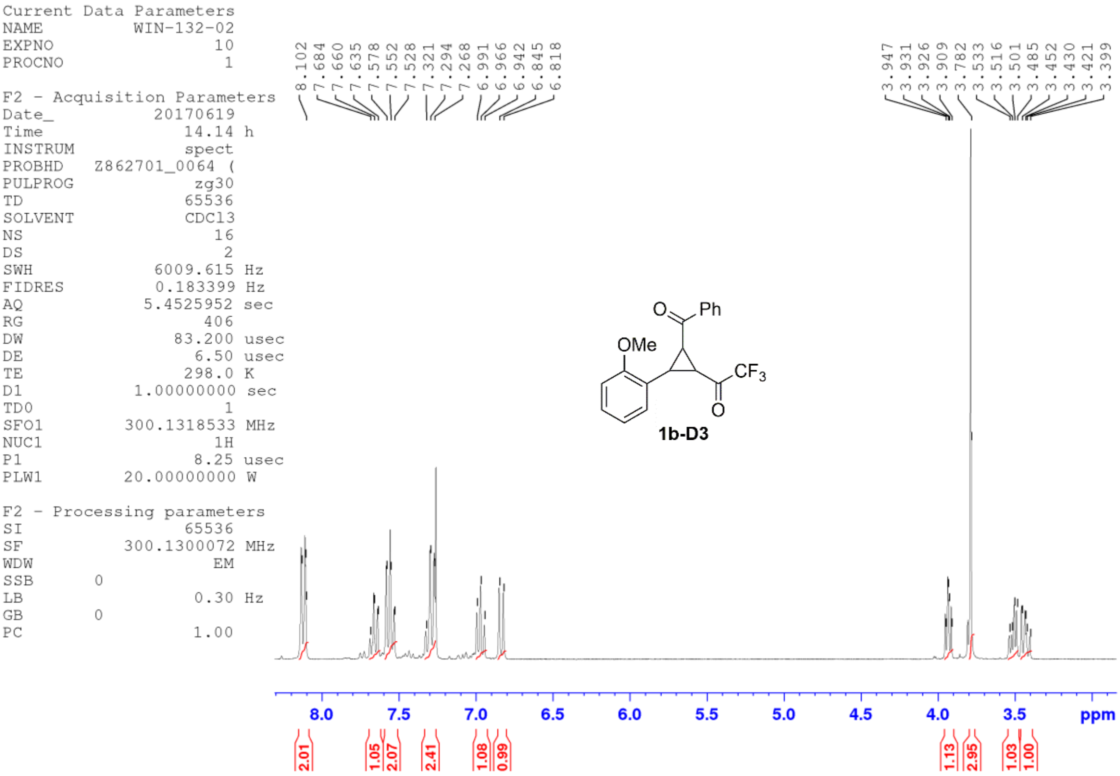
Current Data Parameters
NAME WIN-132-02
EXPNO 10
PROCNO 1
F2 - Acquisition Parameters
Date_ 20170619
Time 14.14 h
INSTRUM spect
PROBHD Z862701_0064 (
PULPROG zg30
TD 65536
SOLVENT CDCl3
NS 16
DS 2
SWH 6009.615 Hz
FIDRES 0.183399 Hz
AQ 5.4525952 sec
RG 406
DW 83.200 usec
DE 6.50 usec
TE 298.0 K
D1 1.00000000 sec
TD0 1
SFO1 300.1318533 MHz
NUC1 1H
P1 8.25 usec
PLW1 20.00000000 W
F2 - Processing parameters
SI 65536
SF 300.1300072 MHz
WDW EM
SSB 0
LB 0.30 Hz
GB 0
PC 1.00
8.102 7.684 7.660 7.635 7.578 7.552 7.528 7.321 7.294 7.268 6.991 6.966 6.942 6.845 6.818
3.947 3.931 3.926 3.909 3.782 3.533 3.516 3.501 3.485 3.452 3.430 3.421 3.399
O
Ph
OMe
CF3
O
1b-D3
8.0 7.5 7.0 6.5 6.0 5.5 5.0 4.5 4.0 3.5 ppm
2.01 1.05 2.07 2.41 1.08 0.99 1.13 2.95 1.03 1.00

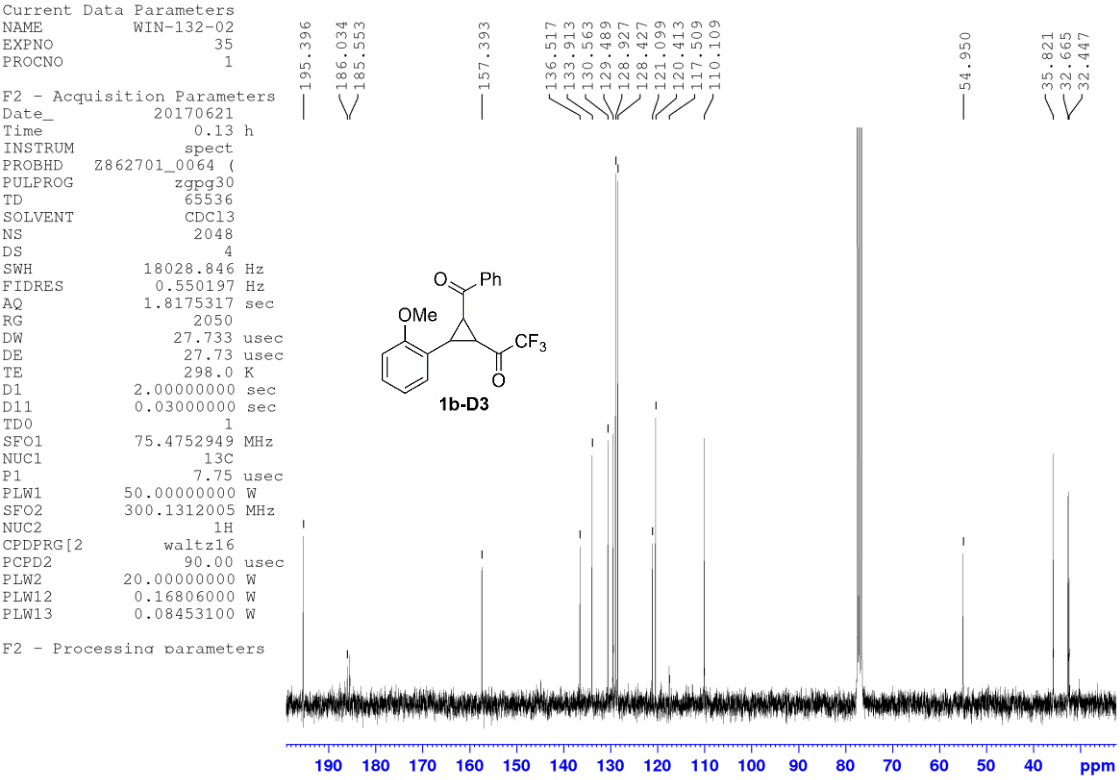
Current Data Parameters
NAME WIN-132-02
EXPNO 35
PROCNO 1
F2 - Acquisition Parameters
Date_ 20170621
Time 0.13 h
INSTRUM spect
PROBHD Z862701_0064 (
PULPROG zgpg30
TD 65536
SOLVENT CDCl3
NS 2048
DS 4
SWH 18028.846 Hz
FIDRES 0.550197 Hz
AQ 1.8175317 sec
RG 2050
DW 27.733 usec
DE 27.73 usec
TE 298.0 K
D1 2.00000000 sec
D11 0.03000000 sec
TD0 1
SFO1 75.4752949 MHz
NUC1 13C
P1 7.75 usec
PLW1 50.00000000 W
SFO2 300.1312005 MHz
NUC2 1H
CPDPRG[2 waltz16
PCPD2 90.00 usec
PLW2 20.00000000 W
PLW12 0.16806000 W
PLW13 0.08453100 W
F2 - Processing parameters
195.396 186.034 185.553 157.393 136.517 133.913 130.563 129.489 128.927 128.427 121.099 120.413 117.509 110.109 54.950 35.821 32.665 32.447
O
Ph
OMe
CF3
O
1b-D3
190 180 170 160 150 140 130 120 110 100 90 80 70 60 50 40 ppm

```
Current Data Parameters
NAME          WIN-132-02
EXPNO                 12
PROCNO                 1

F2 - Acquisition Parameters
Date_           20170619
Time               15.05 h
INSTRUM            spect
PROBHD   Z862701_0064 (
PULPROG           zgflqn
TD                131072
SOLVENT            CDCl3
NS                    16
DS                     4
SWH            66964.289 Hz
FIDRES          1.021794 Hz
AQ             0.9786710 sec
RG                   645
DW                 7.467 usec
DE                  6.50 usec
TE                 298.0 K
D1            1.00000000 sec
TD0                    1
SFO1         282.3761148 MHz
NUC1                 19F
P1                  8.60 usec
PLW1         19.99900055 W

F2 - Processing parameters
SI                 65536
SF           282.4043550 MHz
WDW                   EM
SSB      0
LB                  0.30 Hz
GB       0
PC                  1.00
```

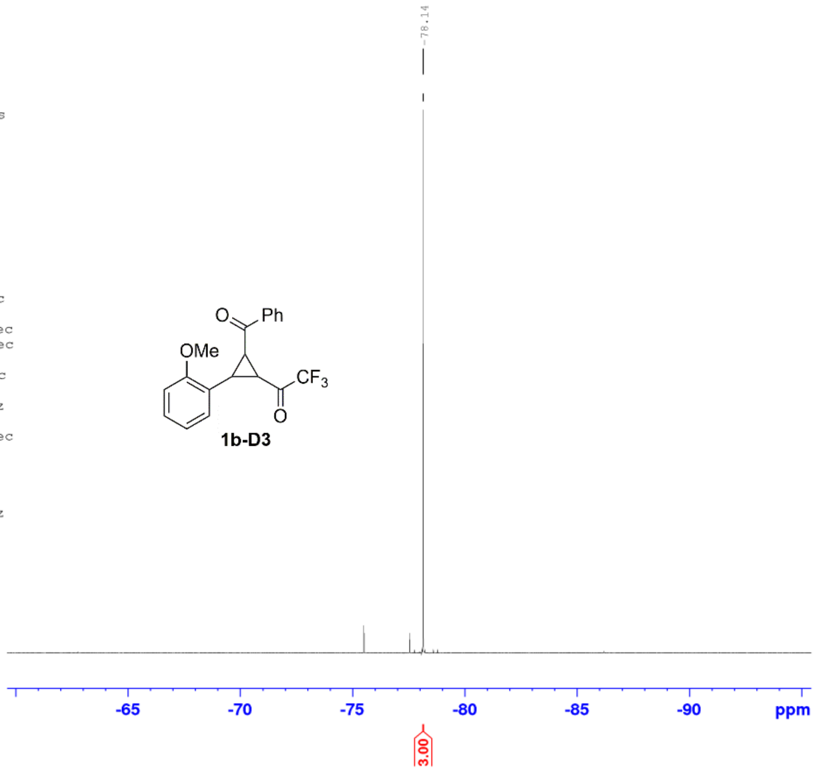


```
Current Data Parameters
NAME          WIN-137-03
EXPNO                 10
PROCNO                 1

F2 - Acquisition Parameters
Date_           20170626
Time               15.33 h
INSTRUM            spect
PROBHD   Z862701_0064 (
PULPROG             zg30
TD                 65536
SOLVENT            CDCl3
NS                    16
DS                     2
SWH             6009.615 Hz
FIDRES          0.183399 Hz
AQ             5.4525952 sec
RG                   362
DW                83.200 usec
DE                  6.50 usec
TE                 298.0 K
D1            1.00000000 sec
TD0                    1
SFO1         300.1318533 MHz
NUC1                  1H
P1                  8.25 usec
PLW1         20.00000000 W

F2 - Processing parameters
SI                 65536
SF           300.1300093 MHz
WDW                   EM
SSB      0
LB                  0.30 Hz
GB       0
PC                  1.00
```

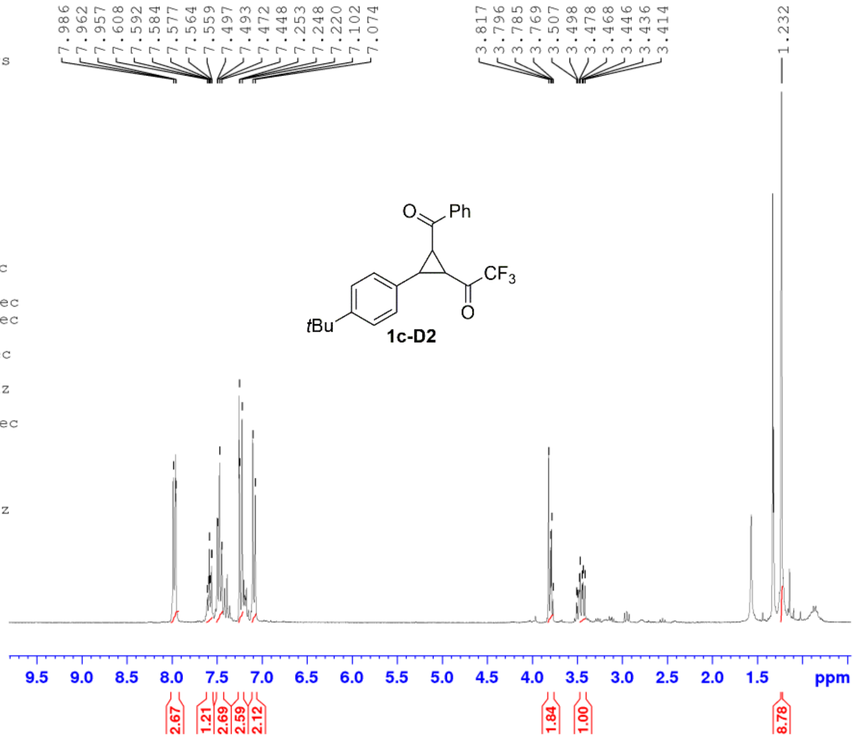

```
Current Data Parameters
NAME          WIN-137-03
EXPNO                 20
PROCNO                 1

F2 - Acquisition Parameters
Date_          20170628
Time               2.29 h
INSTRUM           spect
PROBHD   Z862701_0064 (
PULPROG         zgpg30
TD                65536
SOLVENT           CDCl3
NS                 2048
DS                    4
SWH           18028.846 Hz
FIDRES         0.550197 Hz
AQ            1.8175317 sec
RG                 2050
DW               27.733 usec
DE                27.73 usec
TE                298.0 K
D1           2.00000000 sec
D11          0.03000000 sec
TD0                   1
SFO1         75.4752949 MHz
NUC1                13C
P1                 7.75 usec
PLW1        50.00000000 W
SFO2        300.1312005 MHz
NUC2                 1H
CPDPRG[2        waltz16
PCPD2             90.00 usec
PLW2        20.00000000 W
PLW12        0.16806000 W
PLW13        0.08453100 W

F2 - Processing parameters
```

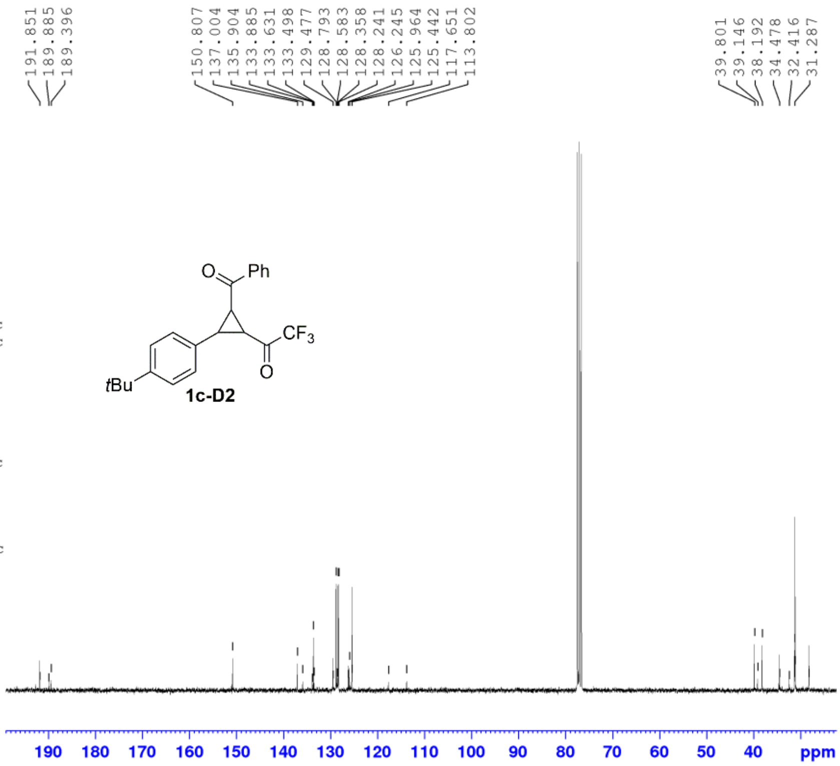


```
Current Data Parameters
NAME          WIN-137-03
EXPNO                 11
PROCNO                 1

F2 - Acquisition Parameters
Date_          20170626
Time              15.34 h
INSTRUM           spect
PROBHD   Z862701_0064 (
PULPROG          zgflqn
TD               131072
SOLVENT           CDCl3
NS                   16
DS                    4
SWH           66964.289 Hz
FIDRES         1.021794 Hz
AQ            0.9786710 sec
RG                  645
DW                7.467 usec
DE                 6.50 usec
TE                298.0 K
D1           1.00000000 sec
TD0                   1
SFO1        282.3761148 MHz
NUC1                19F
P1                 8.60 usec
PLW1        19.99900055 W

F2 - Processing parameters
SI                65536
SF          282.4043550 MHz
WDW                  EM
SSB       0
LB                 0.30 Hz
GB        0
PC                 1.00
```

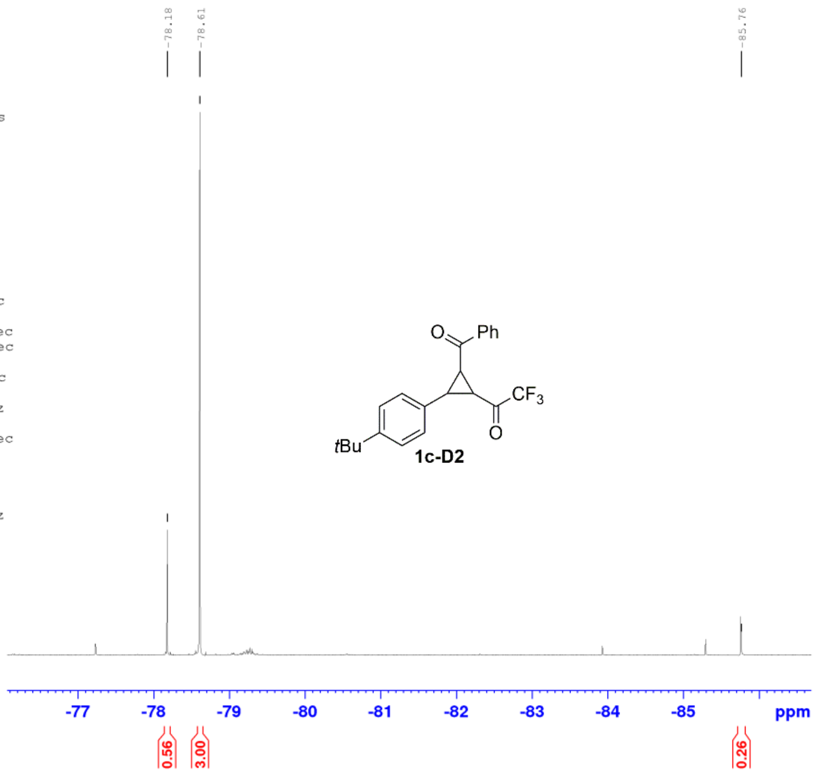

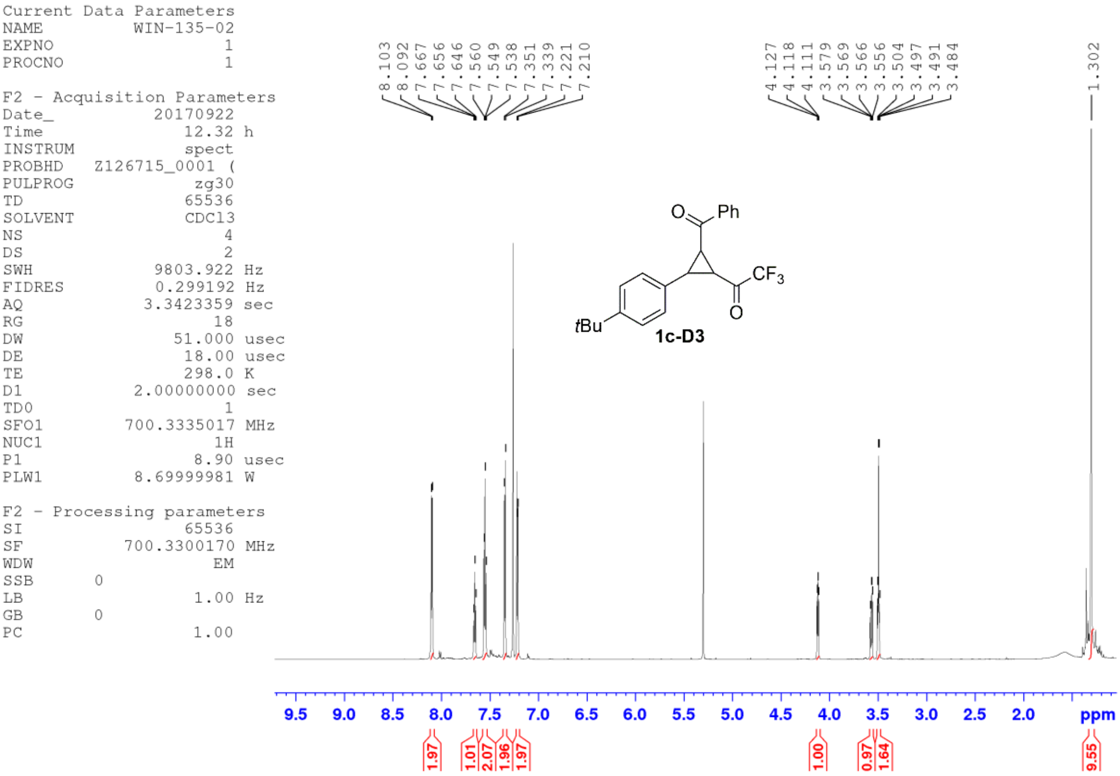
Current Data Parameters
NAME WIN-135-02
EXPNO 1
PROCNO 1
F2 - Acquisition Parameters
Date_ 20170922
Time 12.32 h
INSTRUM spect
PROBHD Z126715_0001 (
PULPROG zg30
TD 65536
SOLVENT CDCl3
NS 4
DS 2
SWH 9803.922 Hz
FIDRES 0.299192 Hz
AQ 3.3423359 sec
RG 18
DW 51.000 usec
DE 18.00 usec
TE 298.0 K
D1 2.00000000 sec
TD0 1
SFO1 700.3335017 MHz
NUC1 1H
P1 8.90 usec
PLW1 8.69999981 W
F2 - Processing parameters
SI 65536
SF 700.3300170 MHz
WDW EM
SSB 0
LB 1.00 Hz
GB 0
PC 1.00
8.103 8.092 7.667 7.656 7.646 7.560 7.549 7.538 7.351 7.339 7.221 7.210
4.127 4.118 4.111 3.579 3.569 3.566 3.556 3.504 3.497 3.491 3.484
1.302
O Ph
CF3
O
tBu
1c-D3
9.5 9.0 8.5 8.0 7.5 7.0 6.5 6.0 5.5 5.0 4.5 4.0 3.5 3.0 2.5 2.0 ppm
1.97 1.01 2.07 1.96 1.97 1.00 0.97 1.64 9.55

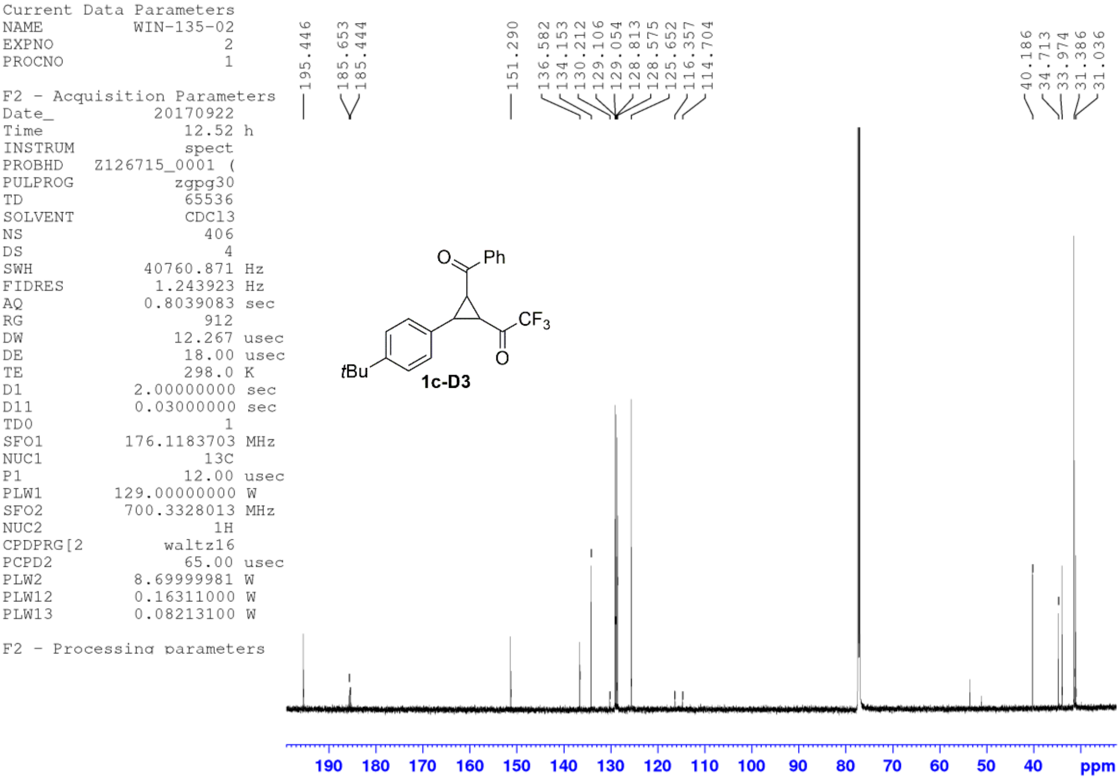
Current Data Parameters
NAME WIN-135-02
EXPNO 2
PROCNO 1
F2 - Acquisition Parameters
Date_ 20170922
Time 12.52 h
INSTRUM spect
PROBHD Z126715_0001 (
PULPROG zgpg30
TD 65536
SOLVENT CDCl3
NS 406
DS 4
SWH 40760.871 Hz
FIDRES 1.243923 Hz
AQ 0.8039083 sec
RG 912
DW 12.267 usec
DE 18.00 usec
TE 298.0 K
D1 2.00000000 sec
D11 0.03000000 sec
TD0 1
SFO1 176.1183703 MHz
NUC1 13C
P1 12.00 usec
PLW1 129.00000000 W
SFO2 700.3328013 MHz
NUC2 1H
CPDPRG[2 waltz16
PCPD2 65.00 usec
PLW2 8.69999981 W
PLW12 0.16311000 W
PLW13 0.08213100 W
F2 - Processing parameters
195.446
185.653 185.444
151.290
136.582 134.153 130.212 129.106 129.054 128.813 128.575 125.652 116.357 114.704
40.186 34.713 33.974 31.386 31.036
O Ph
CF3
O
tBu
1c-D3
190 180 170 160 150 140 130 120 110 100 90 80 70 60 50 40 ppm

```
Current Data Parameters
NAME          WIN-135-02
EXPNO                 11
PROCNO                 1

F2 - Acquisition Parameters
Date_           20170623
Time               15.58 h
INSTRUM            spect
PROBHD   Z862701_0064 (
PULPROG          zgflqn
TD               131072
SOLVENT            CDCl3
NS                    16
DS                     4
SWH            66964.289 Hz
FIDRES          1.021794 Hz
AQ             0.9786710 sec
RG                   645
DW                 7.467 usec
DE                  6.50 usec
TE                 298.0 K
D1            1.00000000 sec
TD0                    1
SFO1         282.3761148 MHz
NUC1                 19F
P1                  8.60 usec
PLW1         19.99900055 W

F2 - Processing parameters
SI                 65536
SF           282.4043550 MHz
WDW                   EM
SSB       0
LB                  0.30 Hz
GB        0
PC                  1.00
```

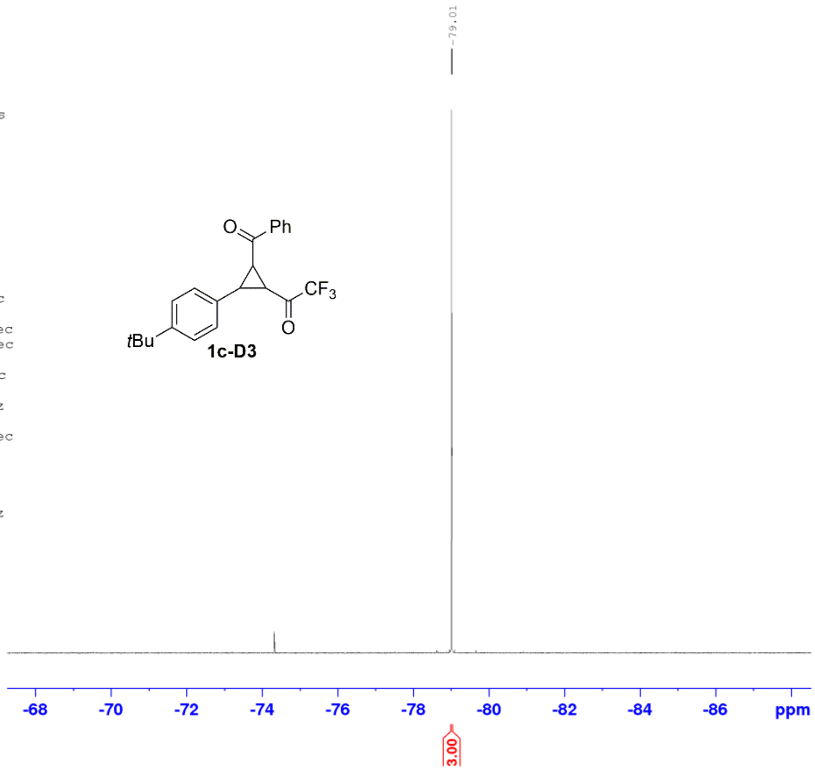


```
Current Data Parameters
NAME          WIN-138-03
EXPNO                 10
PROCNO                 1

F2 - Acquisition Parameters
Date_           20170627
Time               14.29 h
INSTRUM            spect
PROBHD   Z862701_0064 (
PULPROG             zg30
TD                 65536
SOLVENT            CDCl3
NS                    16
DS                     2
SWH             6009.615 Hz
FIDRES          0.183399 Hz
AQ             5.4525952 sec
RG                   575
DW                83.200 usec
DE                  6.50 usec
TE                 298.0 K
D1            1.00000000 sec
TD0                    1
SFO1         300.1318533 MHz
NUC1                  1H
P1                  8.25 usec
PLW1         20.00000000 W

F2 - Processing parameters
SI                 65536
SF           300.1300089 MHz
WDW                   EM
SSB       0
LB                  0.30 Hz
GB        0
PC                  1.00
```

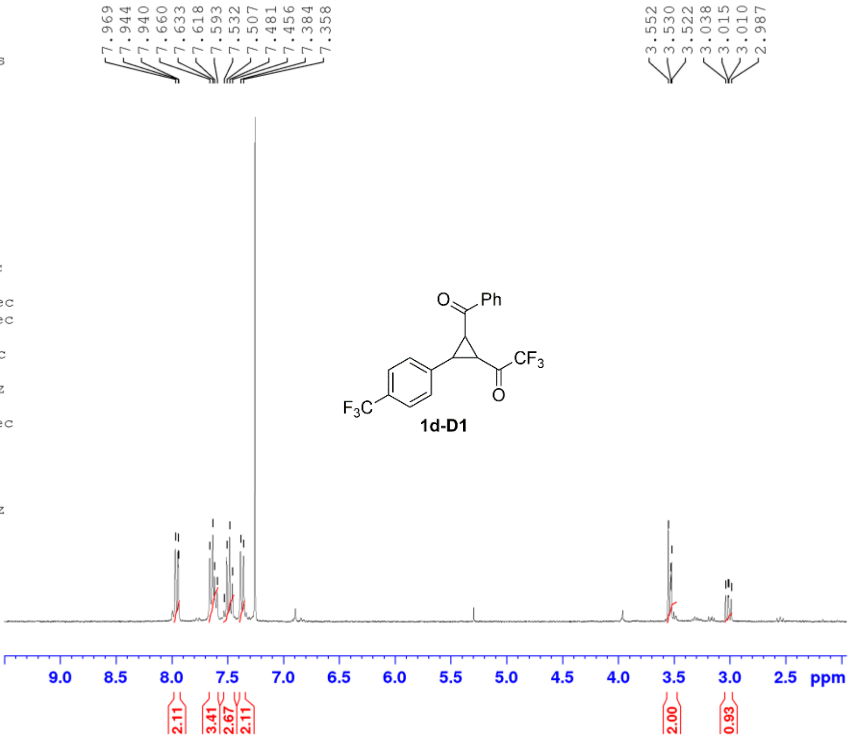

```
Current Data Parameters
NAME          WIN-138-03
EXPNO                 11
PROCNO                 1

F2 - Acquisition Parameters
Date_           20170627
Time               14.30 h
INSTRUM            spect
PROBHD   Z862701_0064 (
PULPROG           zgflqn
TD                131072
SOLVENT            CDCl3
NS                    16
DS                     4
SWH            66964.289 Hz
FIDRES          1.021794 Hz
AQ             0.9786710 sec
RG                   724
DW                 7.467 usec
DE                  6.50 usec
TE                 298.0 K
D1            1.00000000 sec
TD0                    1
SFO1         282.3761148 MHz
NUC1                 19F
P1                  8.60 usec
PLW1         19.99900055 W

F2 - Processing parameters
SI                 65536
SF           282.4043550 MHz
WDW                   EM
SSB      0
LB                  0.30 Hz
GB       0
PC                  1.00
```

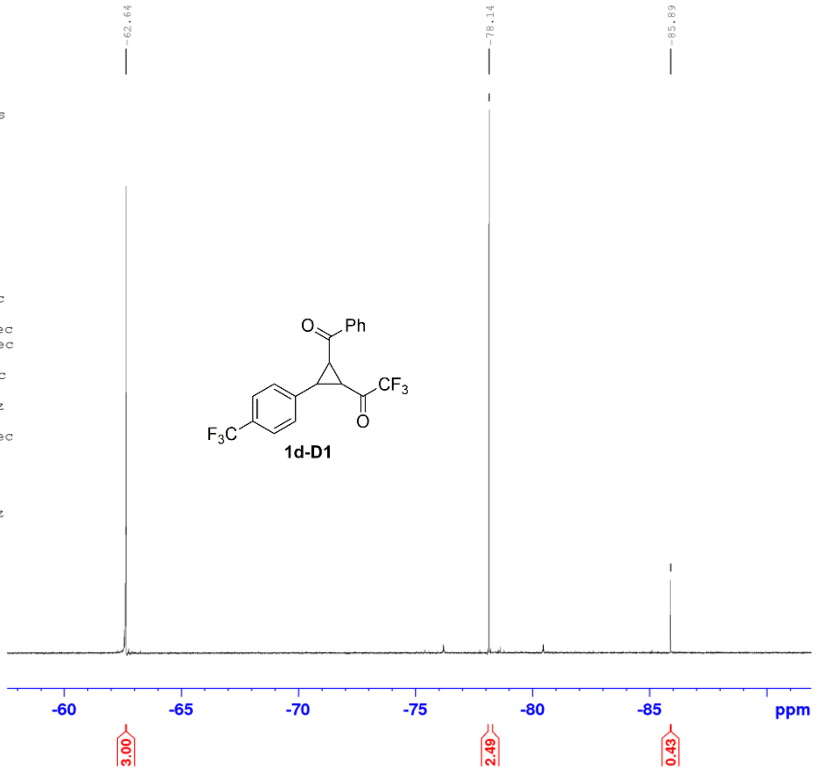


```
Current Data Parameters
NAME          WIN-138-05
EXPNO                  1
PROCNO                 1

F2 - Acquisition Parameters
Date_           20170921
Time                8.22 h
INSTRUM            spect
PROBHD   Z126715_0001 (
PULPROG             zg30
TD                 65536
SOLVENT            CDCl3
NS                     4
DS                     2
SWH             9803.922 Hz
FIDRES          0.299192 Hz
AQ             3.3423359 sec
RG                  11.3
DW                51.000 usec
DE                 18.00 usec
TE                 298.0 K
D1            2.00000000 sec
TD0                    1
SFO1         700.3335017 MHz
NUC1                  1H
P1                  8.90 usec
PLW1          8.69999981 W

F2 - Processing parameters
SI                 65536
SF           700.3300170 MHz
WDW                   EM
SSB      0
LB                  1.00 Hz
GB       0
PC                  1.00
```

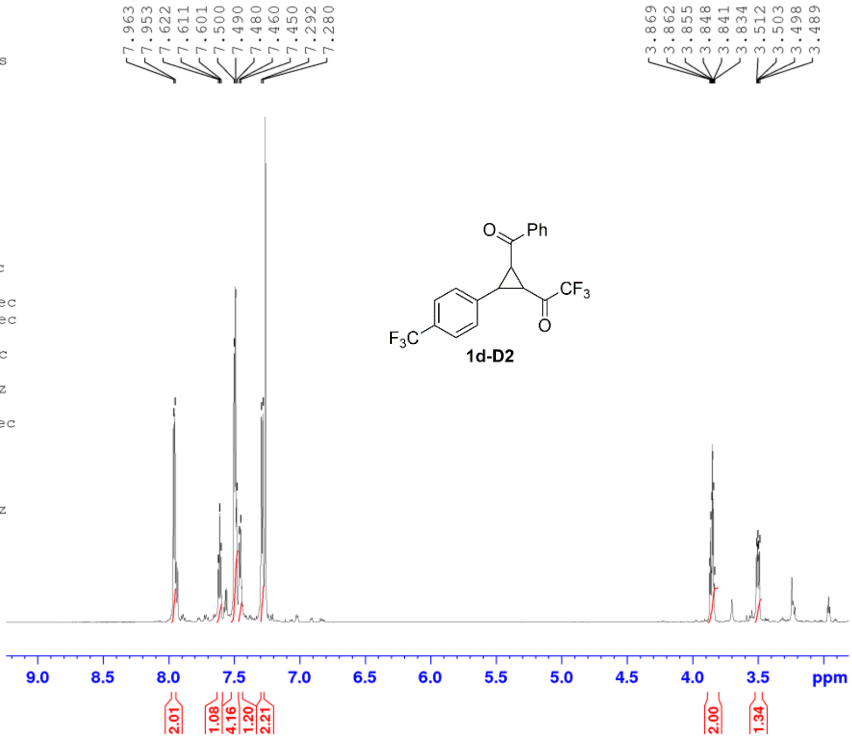

Current Data Parameters
NAME WIN-138-05
EXPNO 2
PROCNO 1

F2 - Acquisition Parameters
Date_ 20170921
Time 9.27 h
INSTRUM spect
PROBHD Z126715_0001 (
PULPROG zgpg30
TD 65536
SOLVENT CDCl3
NS 1209
DS 4
SWH 40760.871 Hz
FIDRES 1.243923 Hz
AQ 0.8039083 sec
RG 2050
DW 12.267 usec
DE 18.00 usec
TE 298.0 K
D1 2.00000000 sec
D11 0.03000000 sec
TD0 1
SFO1 176.1183703 MHz
NUC1 13C
P1 12.00 usec
PLW1 129.00000000 W
SFO2 700.3328013 MHz
NUC2 1H
CPDPRG[2 waltz16
PCPD2 65.00 usec
PLW2 8.69999981 W
PLW12 0.16311000 W
PLW13 0.08213100 W

F2 - Processing parameters

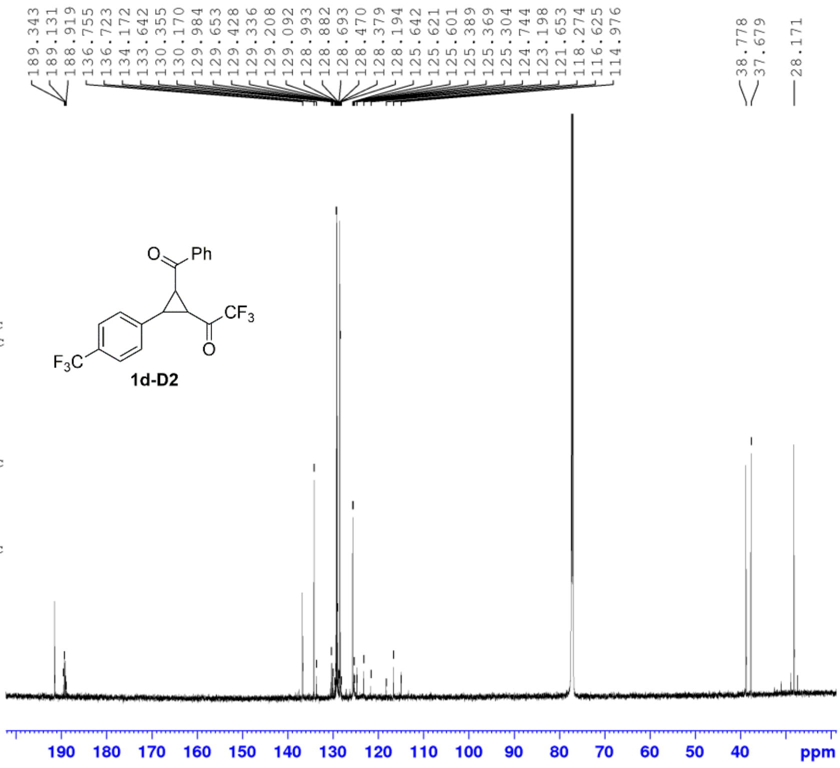

Current Data Parameters
NAME WIN-138-05
EXPNO 11
PROCNO 1

F2 - Acquisition Parameters
Date_ 20170627
Time 14.42 h
INSTRUM spect
PROBHD Z862701_0064 (
PULPROG zgflqn
TD 131072
SOLVENT CDCl3
NS 16
DS 4
SWH 66964.289 Hz
FIDRES 1.021794 Hz
AQ 0.9786710 sec
RG 724
DW 7.467 usec
DE 6.50 usec
TE 298.0 K
D1 1.00000000 sec
TD0 1
SFO1 282.3761148 MHz
NUC1 19F
P1 8.60 usec
PLW1 19.99900055 W

F2 - Processing parameters
SI 65536
SF 282.4043550 MHz
WDW EM
SSB 0
LB 0.30 Hz
GB 0
PC 1.00

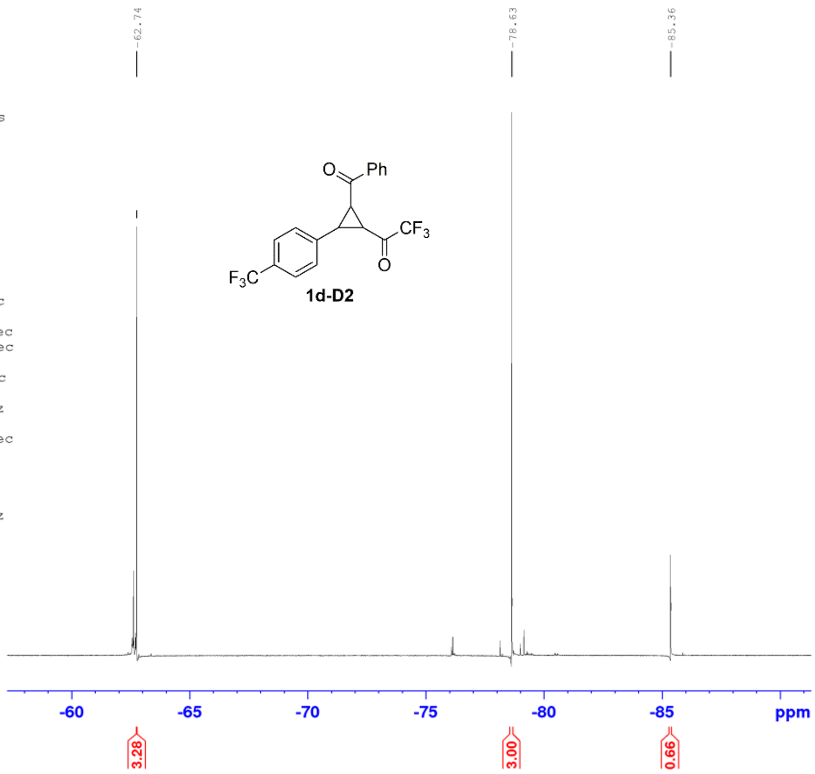

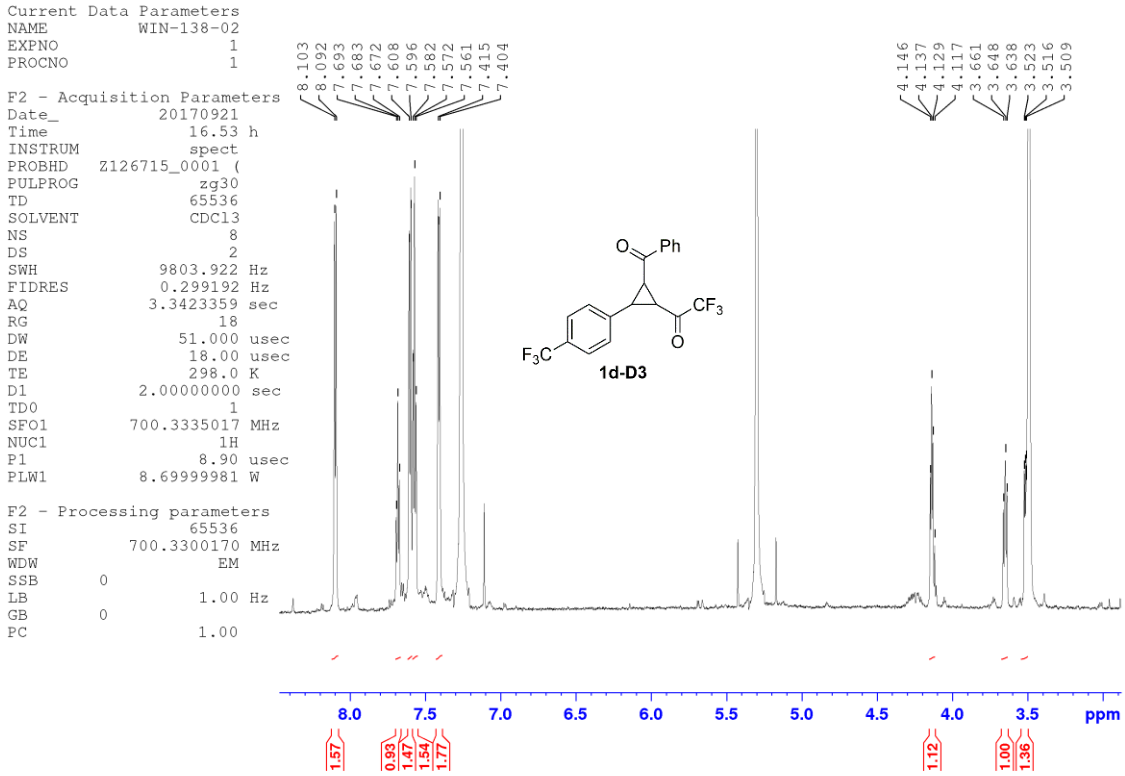
Current Data Parameters
NAME WIN-138-02
EXPNO 1
PROCNO 1
F2 - Acquisition Parameters
Date_ 20170921
Time 16.53 h
INSTRUM spect
PROBHD Z126715_0001 (
PULPROG zg30
TD 65536
SOLVENT CDCl3
NS 8
DS 2
SWH 9803.922 Hz
FIDRES 0.299192 Hz
AQ 3.3423359 sec
RG 18
DW 51.000 usec
DE 18.00 usec
TE 298.0 K
D1 2.00000000 sec
TD0 1
SFO1 700.3335017 MHz
NUC1 1H
P1 8.90 usec
PLW1 8.69999981 W
F2 - Processing parameters
SI 65536
SF 700.3300170 MHz
WDW EM
SSB 0
LB 1.00 Hz
GB 0
PC 1.00
8.103
8.092
7.693
7.683
7.672
7.608
7.596
7.582
7.572
7.561
7.415
7.404
4.146
4.137
4.129
4.117
3.661
3.648
3.638
3.523
3.516
3.509
O
Ph
CF3
O
F3C
1d-D3
8.0
7.5
7.0
6.5
6.0
5.5
5.0
4.5
4.0
3.5
ppm
1.57
0.93
1.47
1.54
1.77
1.12
1.00
1.36

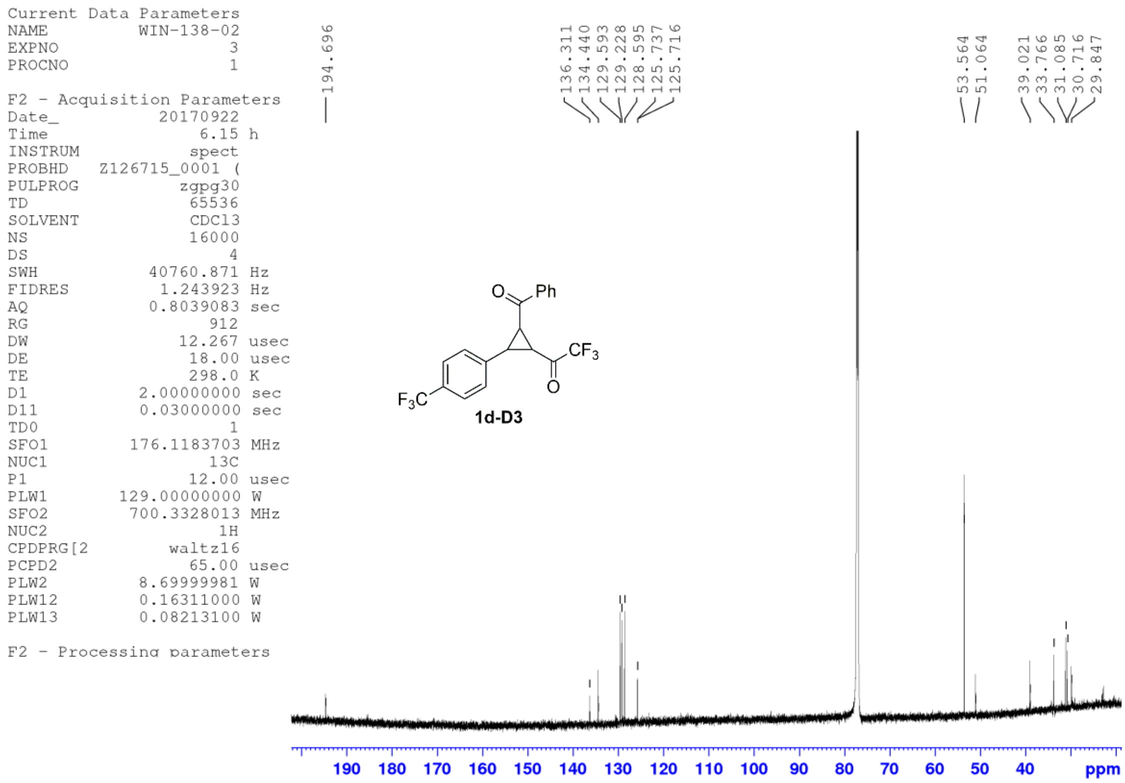
Current Data Parameters
NAME WIN-138-02
EXPNO 3
PROCNO 1
F2 - Acquisition Parameters
Date_ 20170922
Time 6.15 h
INSTRUM spect
PROBHD Z126715_0001 (
PULPROG zgpg30
TD 65536
SOLVENT CDCl3
NS 16000
DS 4
SWH 40760.871 Hz
FIDRES 1.243923 Hz
AQ 0.8039083 sec
RG 912
DW 12.267 usec
DE 18.00 usec
TE 298.0 K
D1 2.00000000 sec
D11 0.03000000 sec
TD0 1
SFO1 176.1183703 MHz
NUC1 13C
P1 12.00 usec
PLW1 129.00000000 W
SFO2 700.3328013 MHz
NUC2 1H
CPDPRG[2 waltz16
PCPD2 65.00 usec
PLW2 8.69999981 W
PLW12 0.16311000 W
PLW13 0.08213100 W
F2 - Processing parameters
194.696
136.311
134.440
129.593
129.228
128.595
125.737
125.716
53.564
51.064
39.021
33.766
31.085
30.716
29.847
O
Ph
CF3
O
F3C
1d-D3
190
180
170
160
150
140
130
120
110
100
90
80
70
60
50
40
ppm

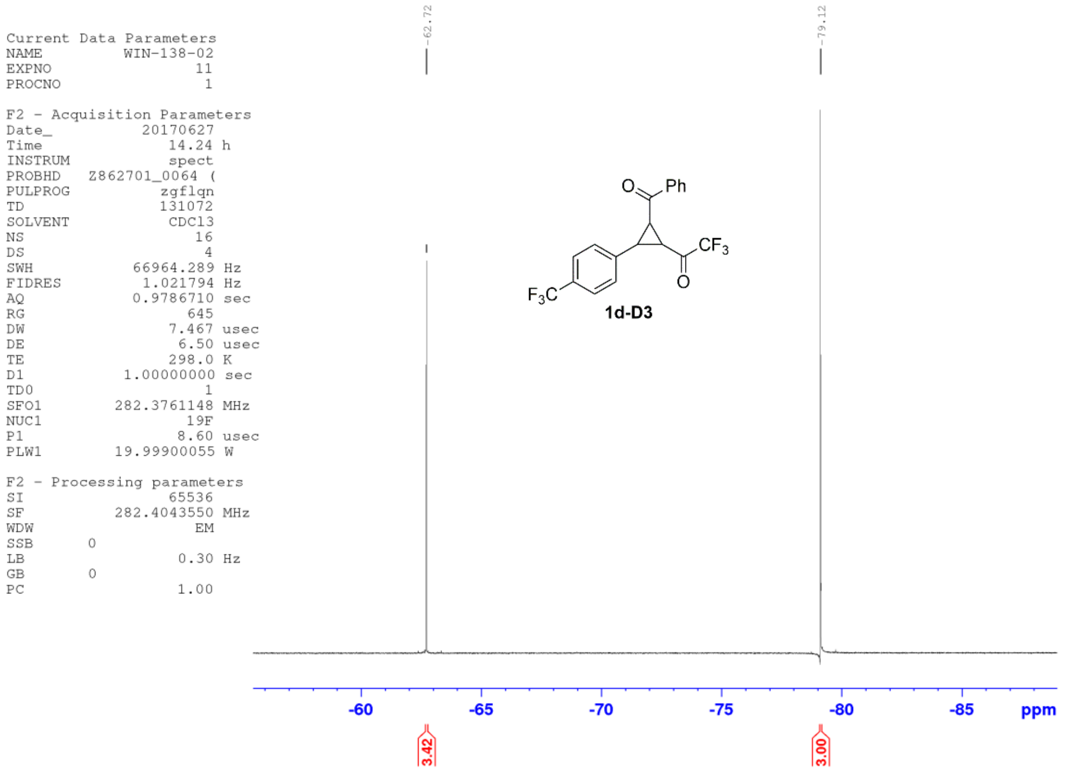
Current Data Parameters
NAME WIN-138-02
EXPNO 11
PROCNO 1
F2 - Acquisition Parameters
Date_ 20170627
Time 14.24 h
INSTRUM spect
PROBHD Z862701_0064 (
PULPROG zgflqn
TD 131072
SOLVENT CDCl3
NS 16
DS 4
SWH 66964.289 Hz
FIDRES 1.021794 Hz
AQ 0.9786710 sec
RG 645
DW 7.467 usec
DE 6.50 usec
TE 298.0 K
D1 1.00000000 sec
TD0 1
SFO1 282.3761148 MHz
NUC1 19F
P1 8.60 usec
PLW1 19.99900055 W
F2 - Processing parameters
SI 65536
SF 282.4043550 MHz
WDW EM
SSB 0
LB 0.30 Hz
GB 0
PC 1.00
-62.72
-79.12
O
Ph
CF3
O
F3C
1d-D3
-60
-65
-70
-75
-80
-85
ppm
3.42
3.00

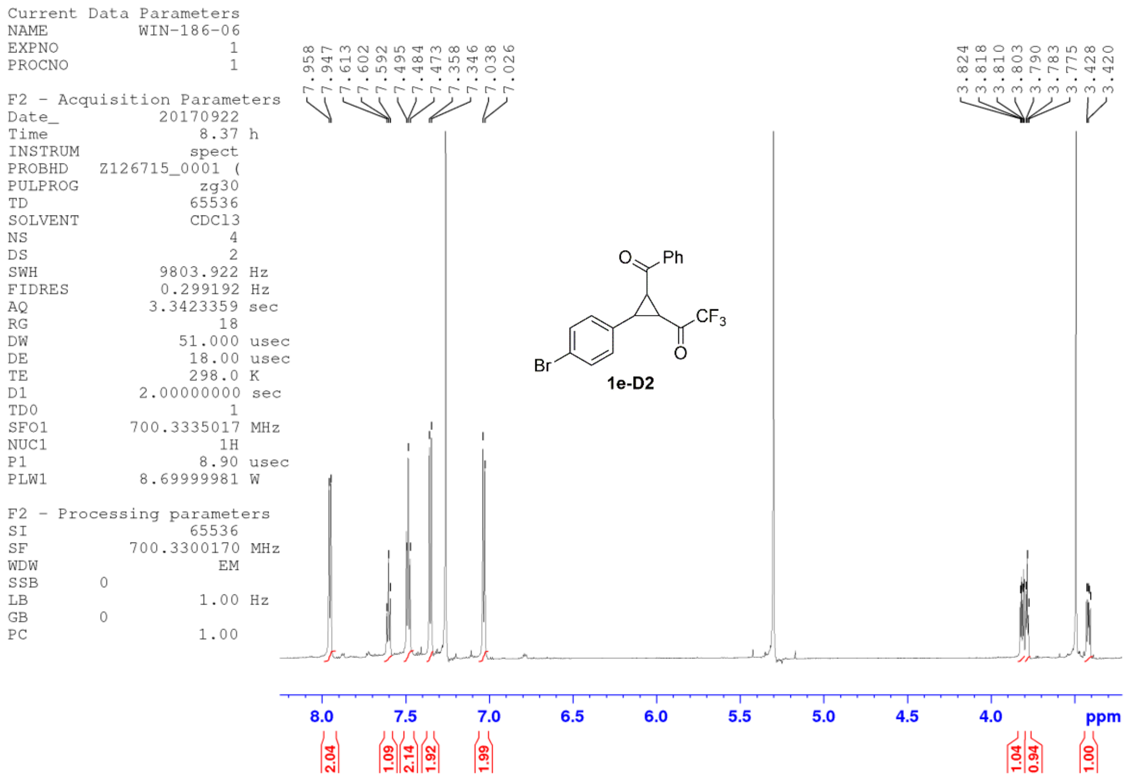
Current Data Parameters
NAME WIN-186-06
EXPNO 1
PROCNO 1
F2 - Acquisition Parameters
Date_ 20170922
Time 8.37 h
INSTRUM spect
PROBHD Z126715_0001 (
PULPROG zg30
TD 65536
SOLVENT CDCl3
NS 4
DS 2
SWH 9803.922 Hz
FIDRES 0.299192 Hz
AQ 3.3423359 sec
RG 18
DW 51.000 usec
DE 18.00 usec
TE 298.0 K
D1 2.00000000 sec
TD0 1
SFO1 700.3335017 MHz
NUC1 1H
P1 8.90 usec
PLW1 8.69999981 W
F2 - Processing parameters
SI 65536
SF 700.3300170 MHz
WDW EM
SSB 0
LB 1.00 Hz
GB 0
PC 1.00
7.958
7.947
7.613
7.602
7.592
7.495
7.484
7.473
7.358
7.346
7.038
7.026
3.824
3.818
3.810
3.803
3.790
3.783
3.775
3.428
3.420
O
Ph
CF3
O
Br
1e-D2
8.0
7.5
7.0
6.5
6.0
5.5
5.0
4.5
4.0
ppm
2.04
1.09
2.14
1.92
1.99
1.04
0.94
1.00

```
Current Data Parameters
NAME            WIN-186-06
EXPNO                    2
PROCNO                   1

F2 - Acquisition Parameters
Date_             20170922
Time                9.23 h
INSTRUM              spect
PROBHD    Z126715_0001 (
PULPROG            zgpg30
TD                  65536
SOLVENT             CDCl3
NS                    934
DS                      4
SWH             40760.871 Hz
FIDRES           1.243923 Hz
AQ              0.8039083 sec
RG                    912
DW                 12.267 usec
DE                  18.00 usec
TE                  298.0 K
D1             2.00000000 sec
D11            0.03000000 sec
TD0                     1
SFO1          176.1183703 MHz
NUC1                  13C
P1                  12.00 usec
PLW1         129.00000000 W
SFO2          700.3328013 MHz
NUC2                   1H
CPDPRG[2          waltz16
PCPD2               65.00 usec
PLW2           8.69999981 W
PLW12          0.16311000 W
PLW13          0.08213100 W

F2 - Processing parameters
```

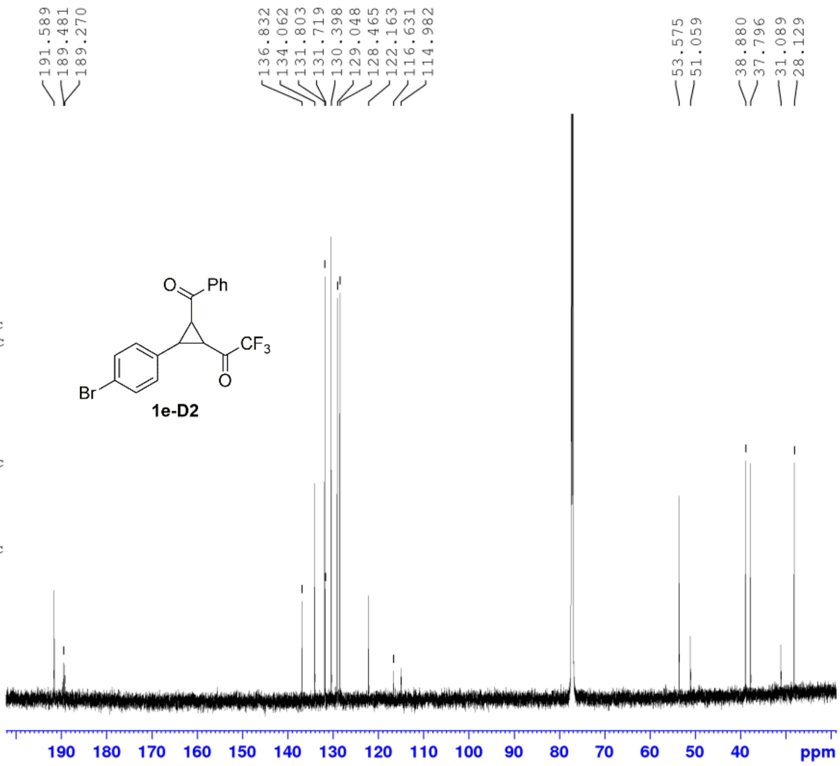


```
Current Data Parameters
NAME            WIN-186-06
EXPNO                   11
PROCNO                   1

F2 - Acquisition Parameters
Date_             20170801
Time               16.59 h
INSTRUM              spect
PROBHD    Z862701_0064 (
PULPROG            zgflqn
TD                 131072
SOLVENT             CDCl3
NS                     16
DS                      4
SWH             66964.289 Hz
FIDRES           1.021794 Hz
AQ              0.9786710 sec
RG                    724
DW                  7.467 usec
DE                   6.50 usec
TE                  298.0 K
D1             1.00000000 sec
TD0                     1
SFO1          282.3761148 MHz
NUC1                  19F
P1                   8.60 usec
PLW1          19.99900055 W

F2 - Processing parameters
SI                  65536
SF            282.4043550 MHz
WDW                    EM
SSB      0
LB                   0.30 Hz
GB       0
PC                   1.00
```

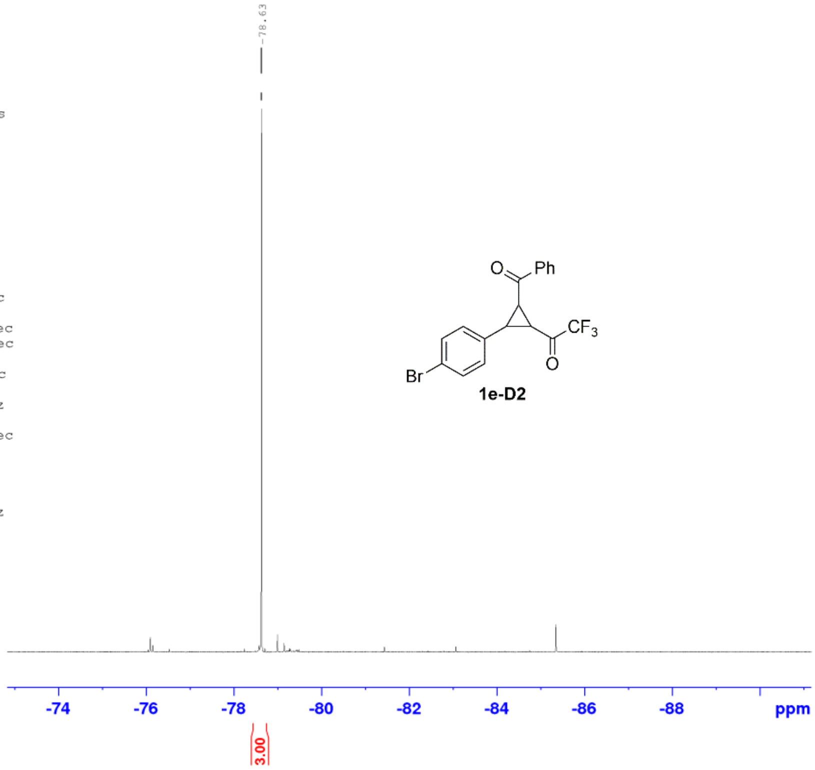

```
Current Data Parameters
NAME          WIN-186-02
EXPNO                  1
PROCNO                 1

F2 - Acquisition Parameters
Date_           20170921
Time               15.29 h
INSTRUM            spect
PROBHD   Z126715_0001 (
PULPROG             zg30
TD                 65536
SOLVENT            CDCl3
NS                     4
DS                     2
SWH             9803.922 Hz
FIDRES          0.299192 Hz
AQ             3.3423359 sec
RG                    18
DW                51.000 usec
DE                 18.00 usec
TE                 298.0 K
D1            2.00000000 sec
TD0                    1
SFO1         700.3335017 MHz
NUC1                  1H
P1                  8.90 usec
PLW1          8.69999981 W

F2 - Processing parameters
SI                 65536
SF           700.3300170 MHz
WDW                   EM
SSB      0
LB                  1.00 Hz
GB       0
PC                  1.00
```

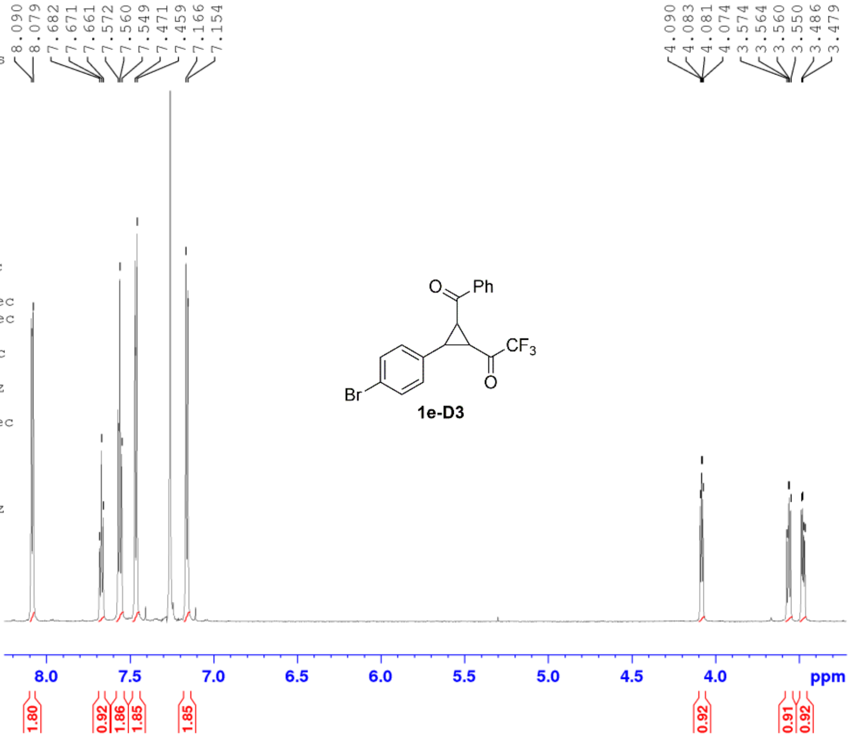


```
Current Data Parameters
NAME          WIN-186-02
EXPNO                  2
PROCNO                 1

F2 - Acquisition Parameters
Date_           20170921
Time               16.44 h
INSTRUM            spect
PROBHD   Z126715_0001 (
PULPROG          zgpg30
TD                 65536
SOLVENT            CDCl3
NS                  1544
DS                     4
SWH            40760.871 Hz
FIDRES          1.243923 Hz
AQ             0.8039083 sec
RG                   912
DW                12.267 usec
DE                 18.00 usec
TE                 298.0 K
D1            2.00000000 sec
D11           0.03000000 sec
TD0                    1
SFO1         176.1183703 MHz
NUC1                 13C
P1                 12.00 usec
PLW1        129.00000000 W
SFO2         700.3328013 MHz
NUC2                  1H
CPDPRG[2         waltz16
PCPD2              65.00 usec
PLW2          8.69999981 W
PLW12         0.16311000 W
PLW13         0.08213100 W

F2 - Processing parameters
```

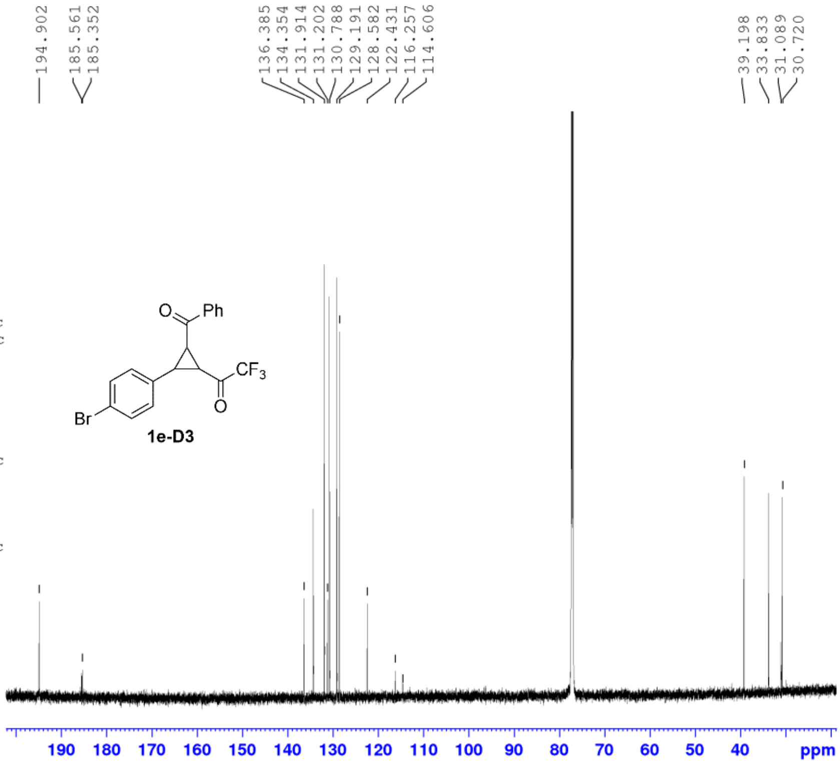

```
Current Data Parameters
NAME            WIN-186-02
EXPNO                   11
PROCNO                   1

F2 - Acquisition Parameters
Date_             20170801
Time               16.41 h
INSTRUM              spect
PROBHD   Z862701_0064 (
PULPROG             zgflqn
TD                  131072
SOLVENT              CDCl3
NS                      16
DS                       4
SWH            66964.289 Hz
FIDRES          1.021794 Hz
AQ             0.9786710 sec
RG                     724
DW                 7.467 usec
DE                  6.50 usec
TE                 298.0 K
D1            1.00000000 sec
TD0                      1
SFO1         282.3761148 MHz
NUC1                   19F
P1                  8.60 usec
PLW1         19.99900055 W

F2 - Processing parameters
SI                   65536
SF           282.4043550 MHz
WDW                     EM
SSB       0
LB                  0.30 Hz
GB        0
PC                  1.00
```

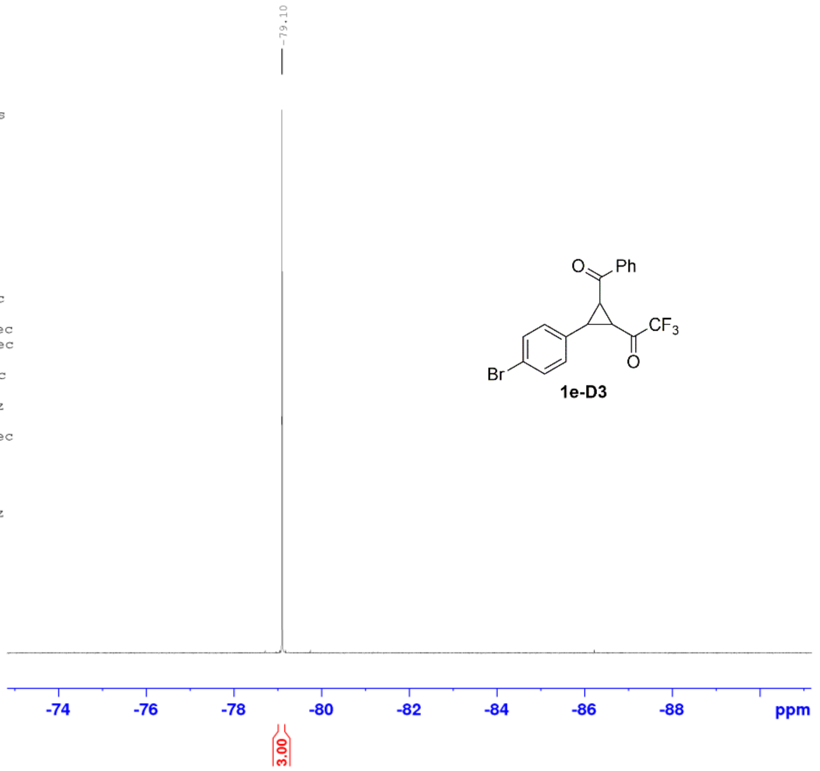


```
Current Data Parameters
NAME            WIN-187-03
EXPNO                    1
PROCNO                   1

F2 - Acquisition Parameters
Date_             20170922
Time               10.25 h
INSTRUM              spect
PROBHD   Z126715_0001 (
PULPROG               zg30
TD                   65536
SOLVENT              CDCl3
NS                       4
DS                       2
SWH             9803.922 Hz
FIDRES          0.299192 Hz
AQ             3.3423359 sec
RG                      18
DW                51.000 usec
DE                 18.00 usec
TE                 298.0 K
D1            2.00000000 sec
TD0                      1
SFO1         700.3335017 MHz
NUC1                    1H
P1                  8.90 usec
PLW1          8.69999981 W

F2 - Processing parameters
SI                   65536
SF           700.3300170 MHz
WDW                     EM
SSB       0
LB                  1.00 Hz
GB        0
PC                  1.00
```

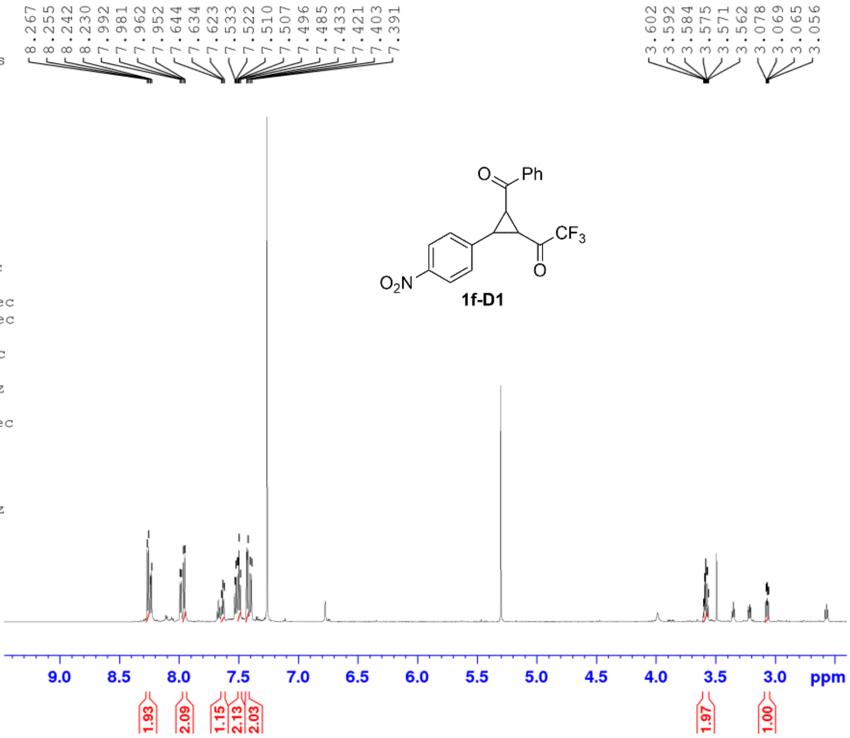

```
Current Data Parameters
NAME          WIN-187-03
EXPNO                 11
PROCNO                 1

F2 - Acquisition Parameters
Date_           20170802
Time               12.11 h
INSTRUM            spect
PROBHD   Z862701_0064 (
PULPROG          zgflqn
TD                131072
SOLVENT            CDCl3
NS                    16
DS                     4
SWH            66964.289 Hz
FIDRES          1.021794 Hz
AQ             0.9786710 sec
RG                   724
DW                 7.467 usec
DE                  6.50 usec
TE                 298.0 K
D1            1.00000000 sec
TD0                    1
SFO1         282.3761148 MHz
NUC1                 19F
P1                  8.60 usec
PLW1         19.99900055 W

F2 - Processing parameters
SI                 65536
SF           282.4043550 MHz
WDW                   EM
SSB      0
LB                  0.30 Hz
GB       0
PC                  1.00
```

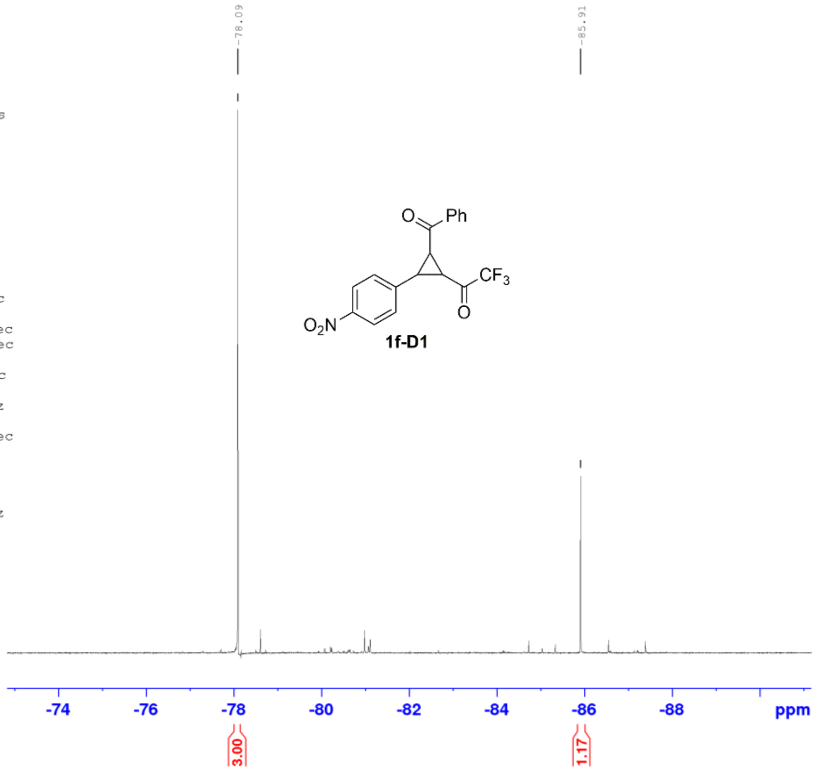


```
Current Data Parameters
NAME          WIN-187-04
EXPNO                  1
PROCNO                 1

F2 - Acquisition Parameters
Date_           20170922
Time               11.20 h
INSTRUM            spect
PROBHD   Z126715_0001 (
PULPROG             zg30
TD                 65536
SOLVENT            CDCl3
NS                     4
DS                     2
SWH             9803.922 Hz
FIDRES          0.299192 Hz
AQ             3.3423359 sec
RG                    18
DW                51.000 usec
DE                 18.00 usec
TE                 298.0 K
D1            2.00000000 sec
TD0                    1
SFO1         700.3335017 MHz
NUC1                  1H
P1                  8.90 usec
PLW1          8.69999981 W

F2 - Processing parameters
SI                 65536
SF           700.3300170 MHz
WDW                   EM
SSB      0
LB                  1.00 Hz
GB       0
PC                  1.00
```

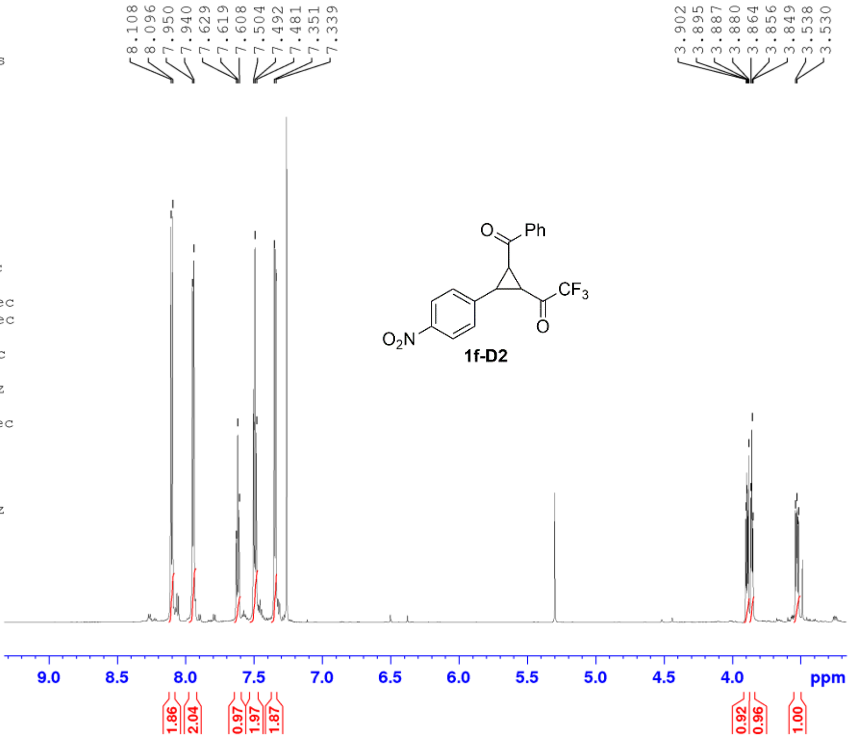

```
Current Data Parameters
NAME          WIN-187-04
EXPNO                  2
PROCNO                 1

F2 - Acquisition Parameters
Date_           20170922
Time               12.30 h
INSTRUM            spect
PROBHD    Z126715_0001 (
PULPROG           zgpg30
TD                 65536
SOLVENT            CDCl3
NS                  1447
DS                     4
SWH            40760.871 Hz
FIDRES          1.243923 Hz
AQ             0.8039083 sec
RG                   912
DW                12.267 usec
DE                 18.00 usec
TE                 298.0 K
D1            2.00000000 sec
D11           0.03000000 sec
TD0                    1
SFO1         176.1183703 MHz
NUC1                 13C
P1                 12.00 usec
PLW1        129.00000000 W
SFO2         700.3328013 MHz
NUC2                  1H
CPDPRG[2         waltz16
PCPD2              65.00 usec
PLW2          8.69999981 W
PLW12         0.16311000 W
PLW13         0.08213100 W

F2 - Processing parameters
```

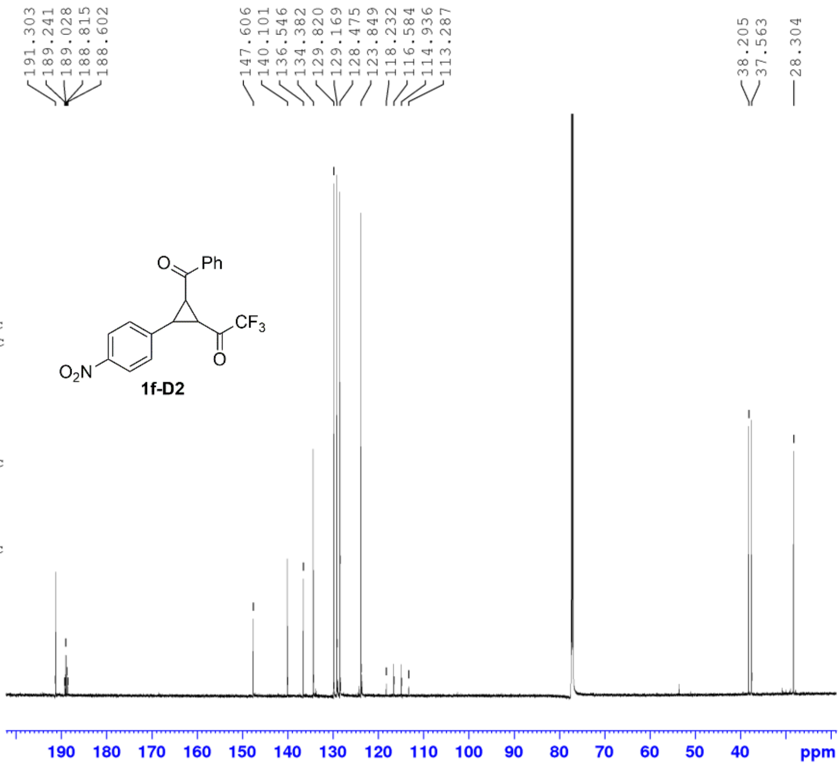


```
Current Data Parameters
NAME          WIN-187-04
EXPNO                 11
PROCNO                 1

F2 - Acquisition Parameters
Date_           20170802
Time               12.17 h
INSTRUM            spect
PROBHD    Z862701_0064 (
PULPROG           zgflqn
TD                131072
SOLVENT            CDCl3
NS                    16
DS                     4
SWH            66964.289 Hz
FIDRES          1.021794 Hz
AQ             0.9786710 sec
RG                   724
DW                 7.467 usec
DE                  6.50 usec
TE                 298.0 K
D1            1.00000000 sec
TD0                    1
SFO1         282.3761148 MHz
NUC1                 19F
P1                  8.60 usec
PLW1         19.99900055 W

F2 - Processing parameters
SI                 65536
SF           282.4043550 MHz
WDW                   EM
SSB      0
LB                  0.30 Hz
GB       0
PC                  1.00
```

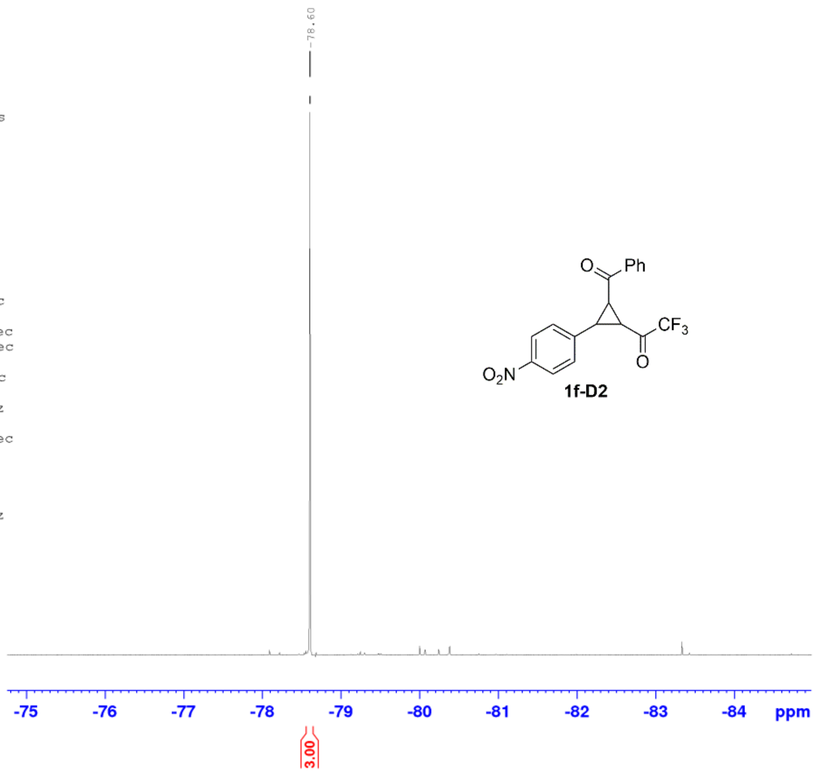

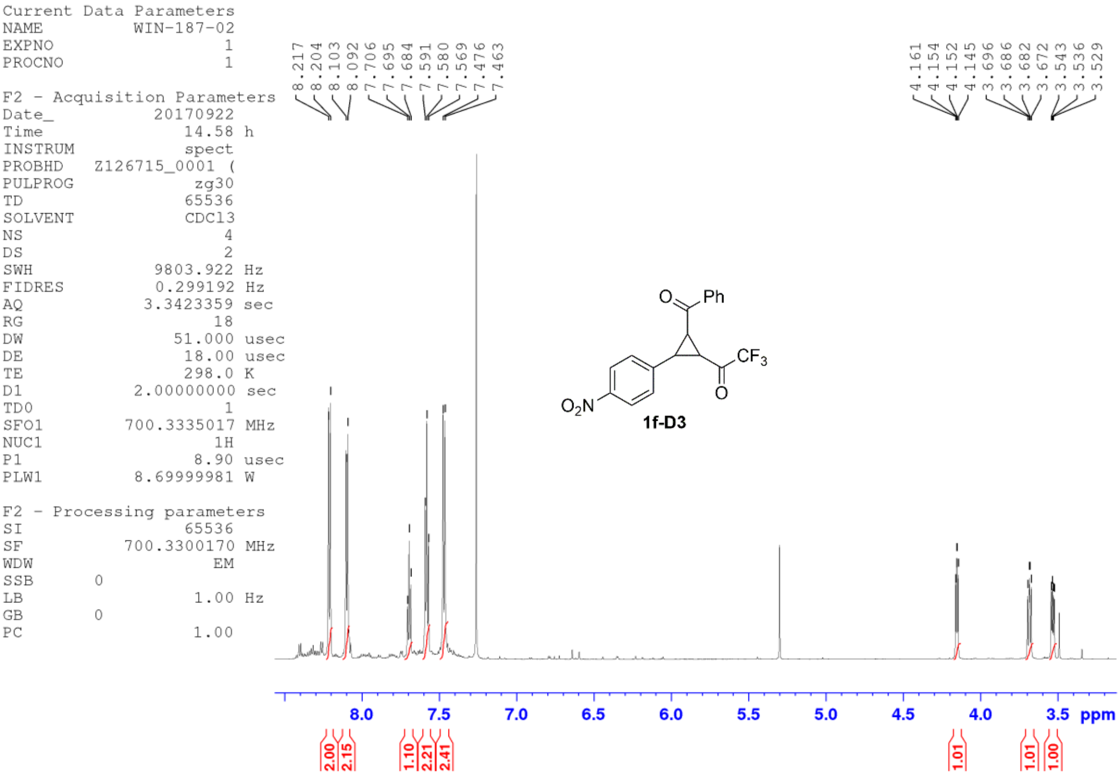
Current Data Parameters
NAME WIN-187-02
EXPNO 1
PROCNO 1
F2 - Acquisition Parameters
Date_ 20170922
Time 14.58 h
INSTRUM spect
PROBHD Z126715_0001 (
PULPROG zg30
TD 65536
SOLVENT CDCl3
NS 4
DS 2
SWH 9803.922 Hz
FIDRES 0.299192 Hz
AQ 3.3423359 sec
RG 18
DW 51.000 usec
DE 18.00 usec
TE 298.0 K
D1 2.00000000 sec
TD0 1
SFO1 700.3335017 MHz
NUC1 1H
P1 8.90 usec
PLW1 8.69999981 W
F2 - Processing parameters
SI 65536
SF 700.3300170 MHz
WDW EM
SSB 0
LB 1.00 Hz
GB 0
PC 1.00
8.217
8.204
8.103
8.092
7.706
7.695
7.684
7.591
7.580
7.569
7.476
7.463
4.161
4.154
4.152
4.145
3.696
3.686
3.682
3.672
3.543
3.536
3.529
O
Ph
CF3
O
O2N
1f-D3
8.0
7.5
7.0
6.5
6.0
5.5
5.0
4.5
4.0
3.5
ppm
2.00
2.15
1.10
2.21
2.41
1.01
1.01
1.00

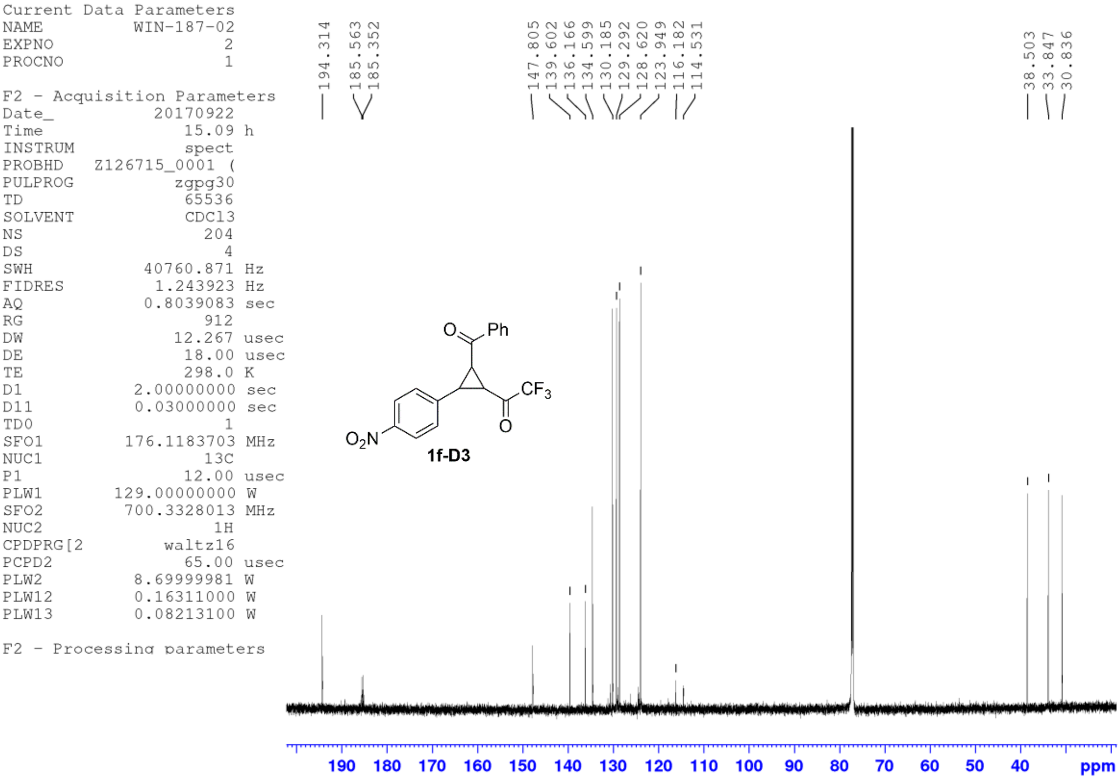
Current Data Parameters
NAME WIN-187-02
EXPNO 2
PROCNO 1
F2 - Acquisition Parameters
Date_ 20170922
Time 15.09 h
INSTRUM spect
PROBHD Z126715_0001 (
PULPROG zgpg30
TD 65536
SOLVENT CDCl3
NS 204
DS 4
SWH 40760.871 Hz
FIDRES 1.243923 Hz
AQ 0.8039083 sec
RG 912
DW 12.267 usec
DE 18.00 usec
TE 298.0 K
D1 2.00000000 sec
D11 0.03000000 sec
TD0 1
SFO1 176.1183703 MHz
NUC1 13C
P1 12.00 usec
PLW1 129.00000000 W
SFO2 700.3328013 MHz
NUC2 1H
CPDPRG[2 waltz16
PCPD2 65.00 usec
PLW2 8.69999981 W
PLW12 0.16311000 W
PLW13 0.08213100 W
F2 - Processing parameters
194.314
185.563
185.352
147.805
139.602
136.166
134.599
130.185
129.292
128.620
123.949
116.182
114.531
38.503
33.847
30.836
O
Ph
CF3
O
O2N
1f-D3
190
180
170
160
150
140
130
120
110
100
90
80
70
60
50
40
ppm

```
Current Data Parameters
NAME         WIN-187-02
EXPNO                11
PROCNO                1

F2 - Acquisition Parameters
Date_          20170802
Time              12.05 h
INSTRUM           spect
PROBHD   Z862701_0064 (
PULPROG          zgflqn
TD               131072
SOLVENT           CDCl3
NS                   16
DS                    4
SWH           66964.289 Hz
FIDRES         1.021794 Hz
AQ            0.9786710 sec
RG                  645
DW                7.467 usec
DE                 6.50 usec
TE                298.0 K
D1           1.00000000 sec
TD0                   1
SFO1        282.3761148 MHz
NUC1                19F
P1                 8.60 usec
PLW1        19.99900055 W

F2 - Processing parameters
SI                65536
SF          282.4043550 MHz
WDW                  EM
SSB      0
LB                 0.30 Hz
GB       0
PC                 1.00
```

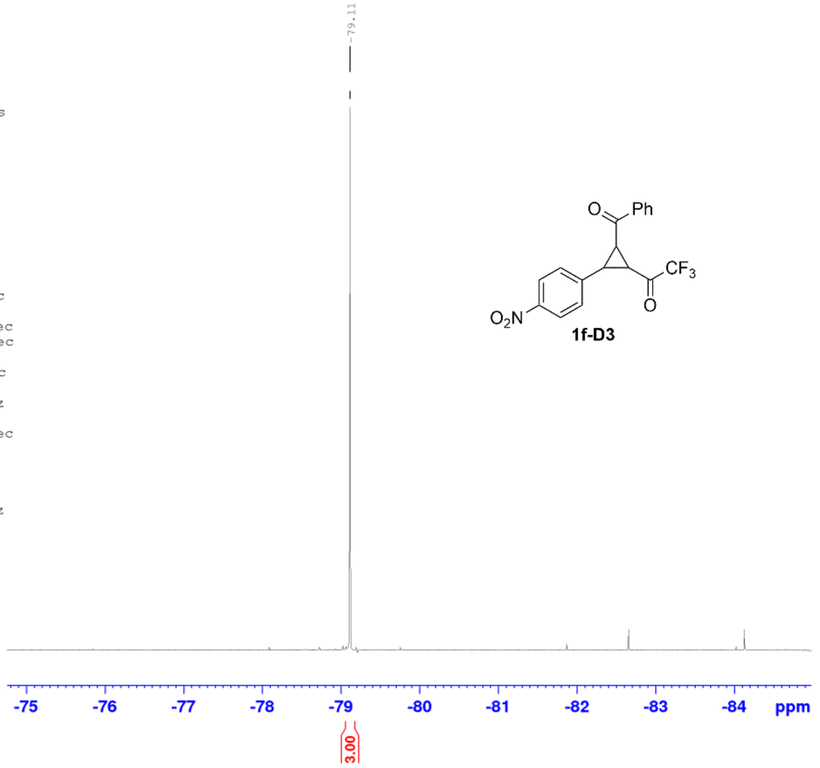


```
Current Data Parameters
NAME         WIN-152-03
EXPNO                 1
PROCNO                1

F2 - Acquisition Parameters
Date_          20170922
Time               9.56 h
INSTRUM           spect
PROBHD   Z126715_0001 (
PULPROG            zg30
TD                65536
SOLVENT           CDCl3
NS                    4
DS                    2
SWH            9803.922 Hz
FIDRES         0.299192 Hz
AQ            3.3423359 sec
RG                   18
DW               51.000 usec
DE                18.00 usec
TE                298.0 K
D1           2.00000000 sec
TD0                   1
SFO1        700.3335017 MHz
NUC1                 1H
P1                 8.90 usec
PLW1         8.69999981 W

F2 - Processing parameters
SI                65536
SF          700.3300170 MHz
WDW                  EM
SSB      0
LB                 1.00 Hz
GB       0
PC                 1.00
```

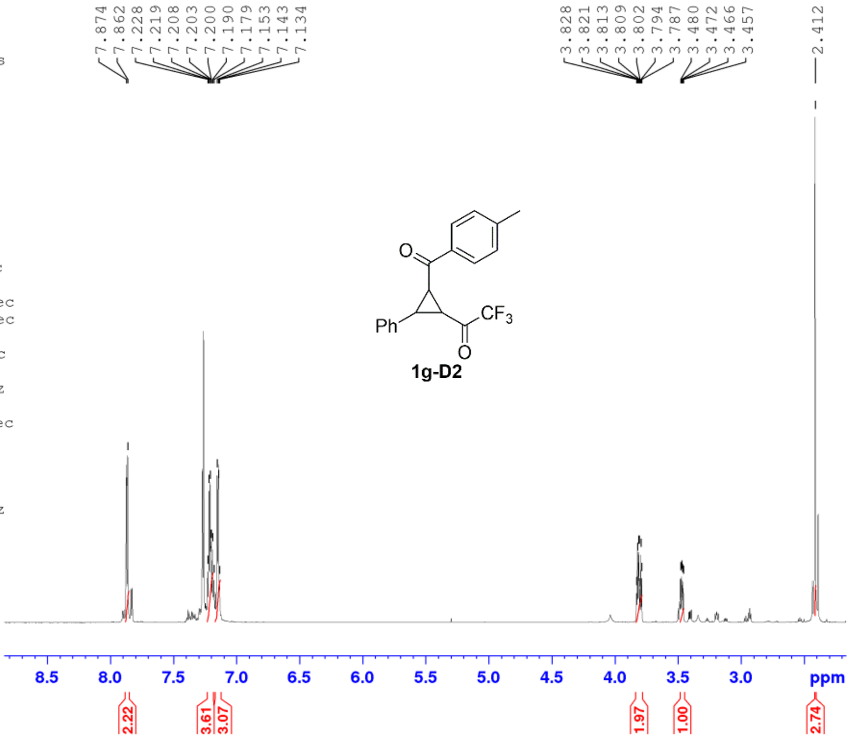

Current Data Parameters
NAME WIN-152-03
EXPNO 2
PROCNO 1

F2 - Acquisition Parameters
Date_ 20170922
Time 10.21 h
INSTRUM spect
PROBHD Z126715_0001 (
PULPROG zgpg30
TD 65536
SOLVENT CDCl3
NS 490
DS 4
SWH 40760.871 Hz
FIDRES 1.243923 Hz
AQ 0.8039083 sec
RG 912
DW 12.267 usec
DE 18.00 usec
TE 298.0 K
D1 2.00000000 sec
D11 0.03000000 sec
TD0 1
SFO1 176.1183703 MHz
NUC1 13C
P1 12.00 usec
PLW1 129.00000000 W
SFO2 700.3328013 MHz
NUC2 1H
CPDPRG[2 waltz16
PCPD2 65.00 usec
PLW2 8.69999981 W
PLW12 0.16311000 W
PLW13 0.08213100 W

F2 - Processing parameters

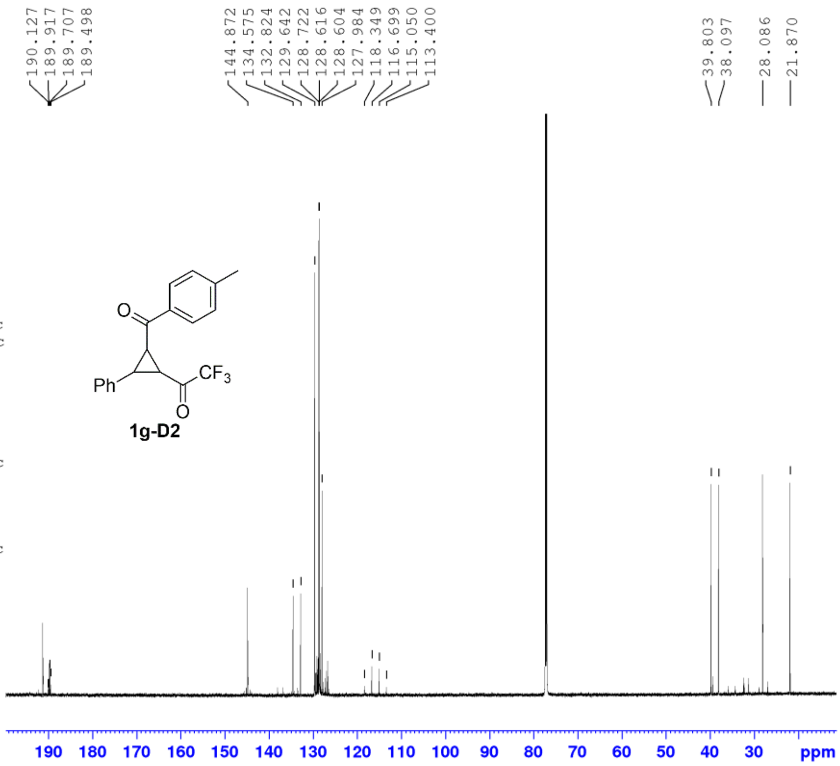

Current Data Parameters
NAME WIN-152-03
EXPNO 11
PROCNO 1

F2 - Acquisition Parameters
Date_ 20170710
Time 15.31 h
INSTRUM spect
PROBHD Z862701_0064 (
PULPROG zgflqn
TD 131072
SOLVENT CDCl3
NS 16
DS 4
SWH 66964.289 Hz
FIDRES 1.021794 Hz
AQ 0.9786710 sec
RG 724
DW 7.467 usec
DE 6.50 usec
TE 298.0 K
D1 1.00000000 sec
TD0 1
SFO1 282.3761148 MHz
NUC1 19F
P1 8.60 usec
PLW1 19.99900055 W

F2 - Processing parameters
SI 65536
SF 282.4043550 MHz
WDW EM
SSB 0
LB 0.30 Hz
GB 0
PC 1.00

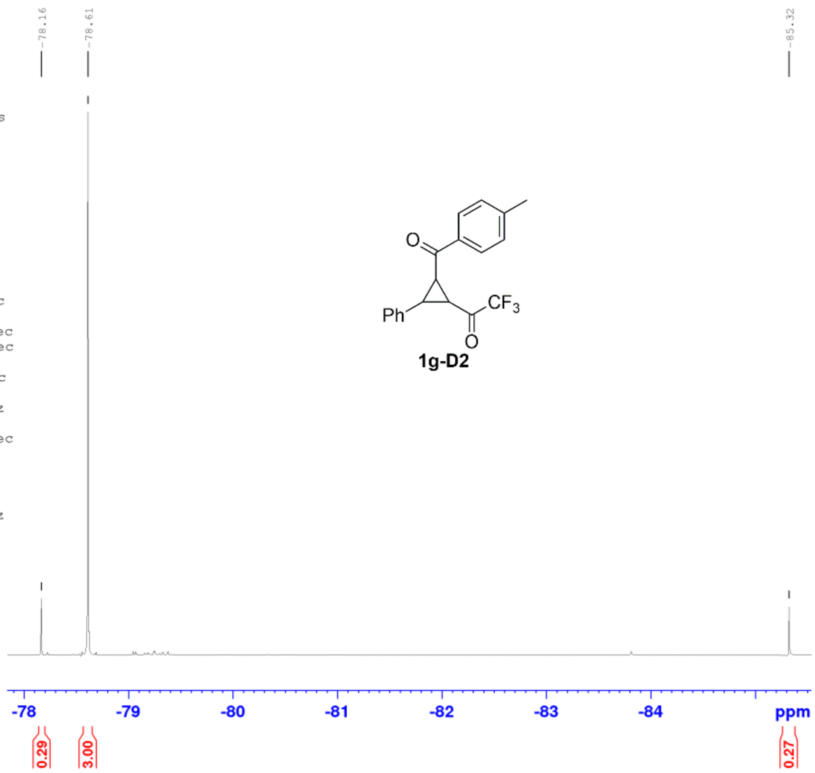

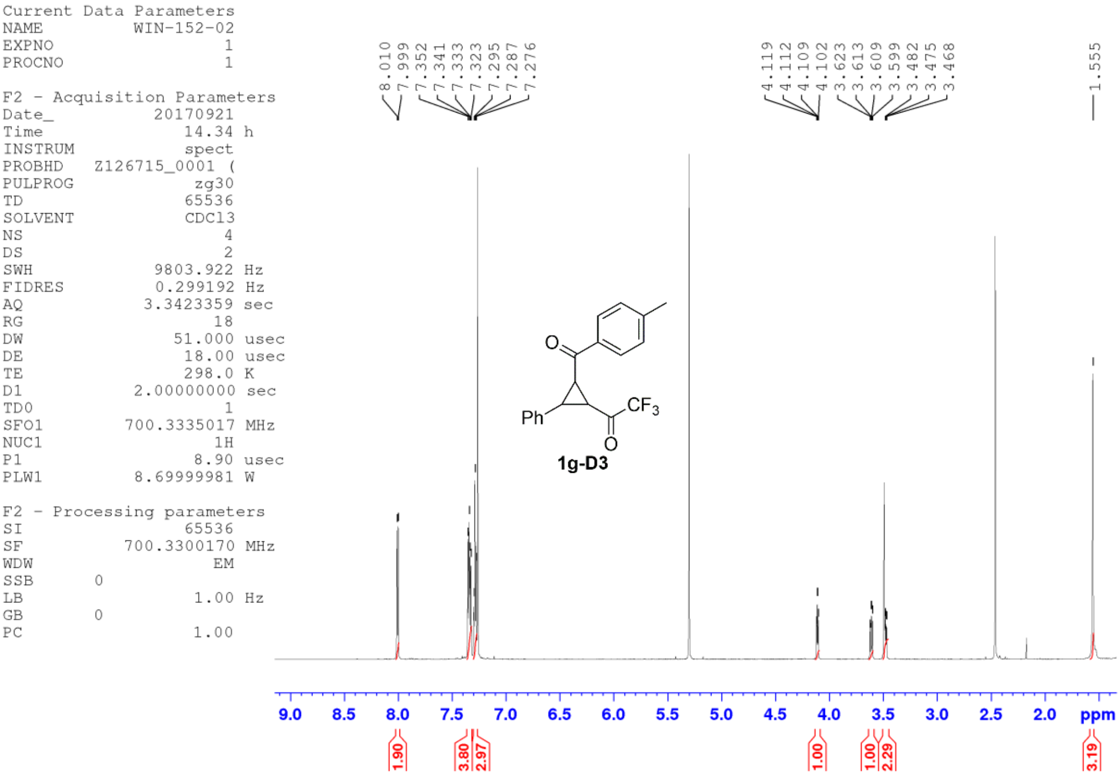
Current Data Parameters
NAME WIN-152-02
EXPNO 1
PROCNO 1
F2 - Acquisition Parameters
Date_ 20170921
Time 14.34 h
INSTRUM spect
PROBHD Z126715_0001 (
PULPROG zg30
TD 65536
SOLVENT CDCl3
NS 4
DS 2
SWH 9803.922 Hz
FIDRES 0.299192 Hz
AQ 3.3423359 sec
RG 18
DW 51.000 usec
DE 18.00 usec
TE 298.0 K
D1 2.00000000 sec
TD0 1
SFO1 700.3335017 MHz
NUC1 1H
P1 8.90 usec
PLW1 8.69999981 W
F2 - Processing parameters
SI 65536
SF 700.3300170 MHz
WDW EM
SSB 0
LB 1.00 Hz
GB 0
PC 1.00
8.010
7.999
7.352
7.341
7.333
7.323
7.295
7.287
7.276
4.119
4.112
4.109
4.102
3.623
3.613
3.609
3.599
3.482
3.475
3.468
1.555
O
Ph
CF3
O
1g-D3
9.0 8.5 8.0 7.5 7.0 6.5 6.0 5.5 5.0 4.5 4.0 3.5 3.0 2.5 2.0 ppm
1.90
3.80
2.97
1.00
1.00
2.29
3.19

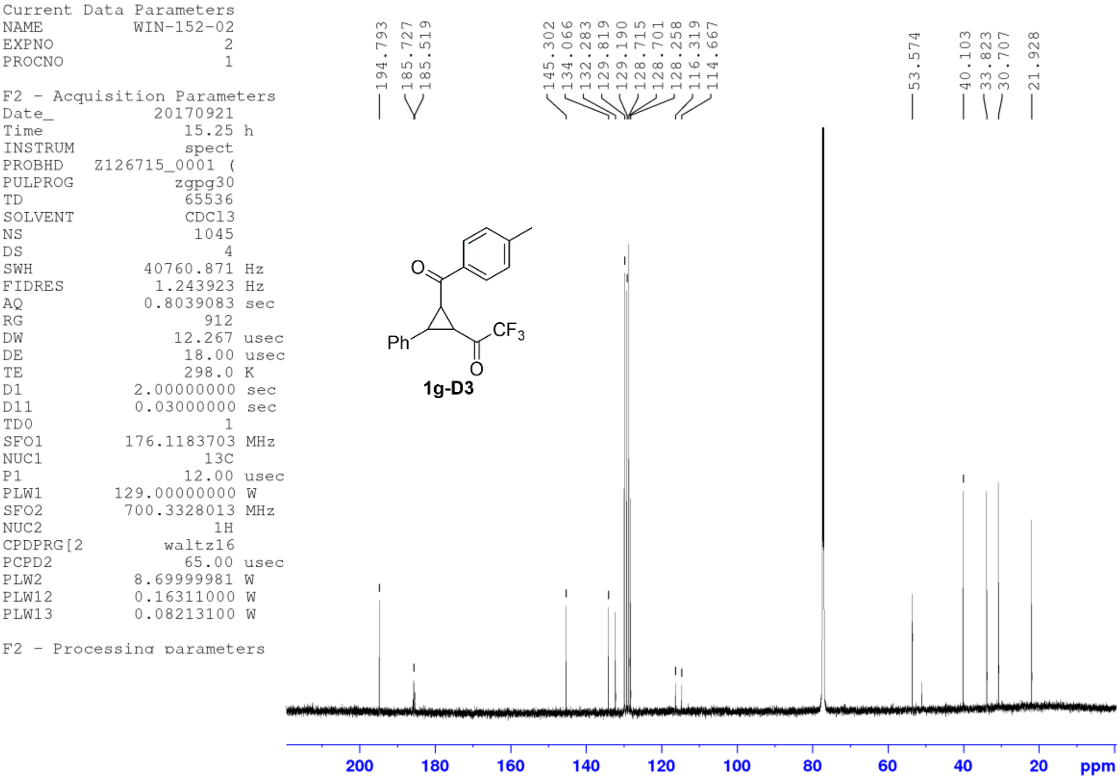
Current Data Parameters
NAME WIN-152-02
EXPNO 2
PROCNO 1
F2 - Acquisition Parameters
Date_ 20170921
Time 15.25 h
INSTRUM spect
PROBHD Z126715_0001 (
PULPROG zgpg30
TD 65536
SOLVENT CDCl3
NS 1045
DS 4
SWH 40760.871 Hz
FIDRES 1.243923 Hz
AQ 0.8039083 sec
RG 912
DW 12.267 usec
DE 18.00 usec
TE 298.0 K
D1 2.00000000 sec
D11 0.03000000 sec
TD0 1
SFO1 176.1183703 MHz
NUC1 13C
P1 12.00 usec
PLW1 129.00000000 W
SFO2 700.3328013 MHz
NUC2 1H
CPDPRG[2 waltz16
PCPD2 65.00 usec
PLW2 8.69999981 W
PLW12 0.16311000 W
PLW13 0.08213100 W
F2 - Processing parameters
194.793
185.727
185.519
145.302
134.066
132.283
129.819
129.190
128.715
128.701
128.258
116.319
114.667
53.574
40.103
33.823
30.707
21.928
O
Ph
CF3
O
1g-D3
200 180 160 140 120 100 80 60 40 20 ppm

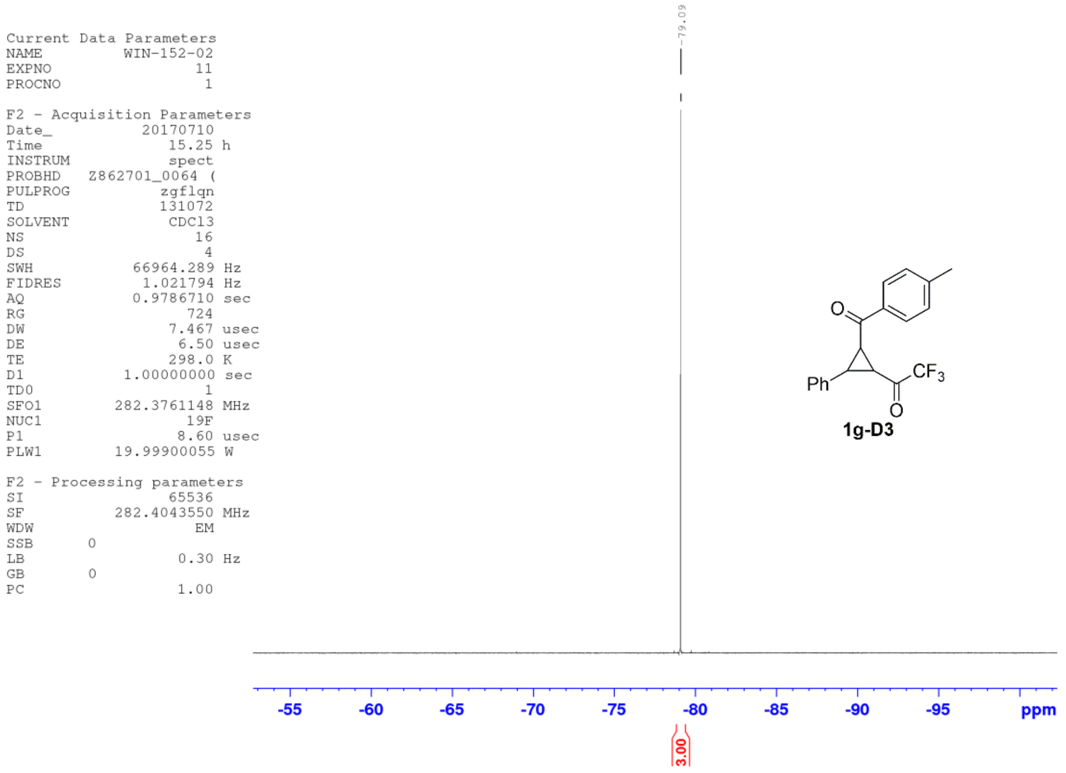
Current Data Parameters
NAME WIN-152-02
EXPNO 11
PROCNO 1
F2 - Acquisition Parameters
Date_ 20170710
Time 15.25 h
INSTRUM spect
PROBHD Z862701_0064 (
PULPROG zgflqn
TD 131072
SOLVENT CDCl3
NS 16
DS 4
SWH 66964.289 Hz
FIDRES 1.021794 Hz
AQ 0.9786710 sec
RG 724
DW 7.467 usec
DE 6.50 usec
TE 298.0 K
D1 1.00000000 sec
TD0 1
SFO1 282.3761148 MHz
NUC1 19F
P1 8.60 usec
PLW1 19.99900055 W
F2 - Processing parameters
SI 65536
SF 282.4043550 MHz
WDW EM
SSB 0
LB 0.30 Hz
GB 0
PC 1.00
-79.09
O
CF_3
Ph
O
1g-D3
-55
-60
-65
-70
-75
-80
-85
-90
-95
ppm
3.00

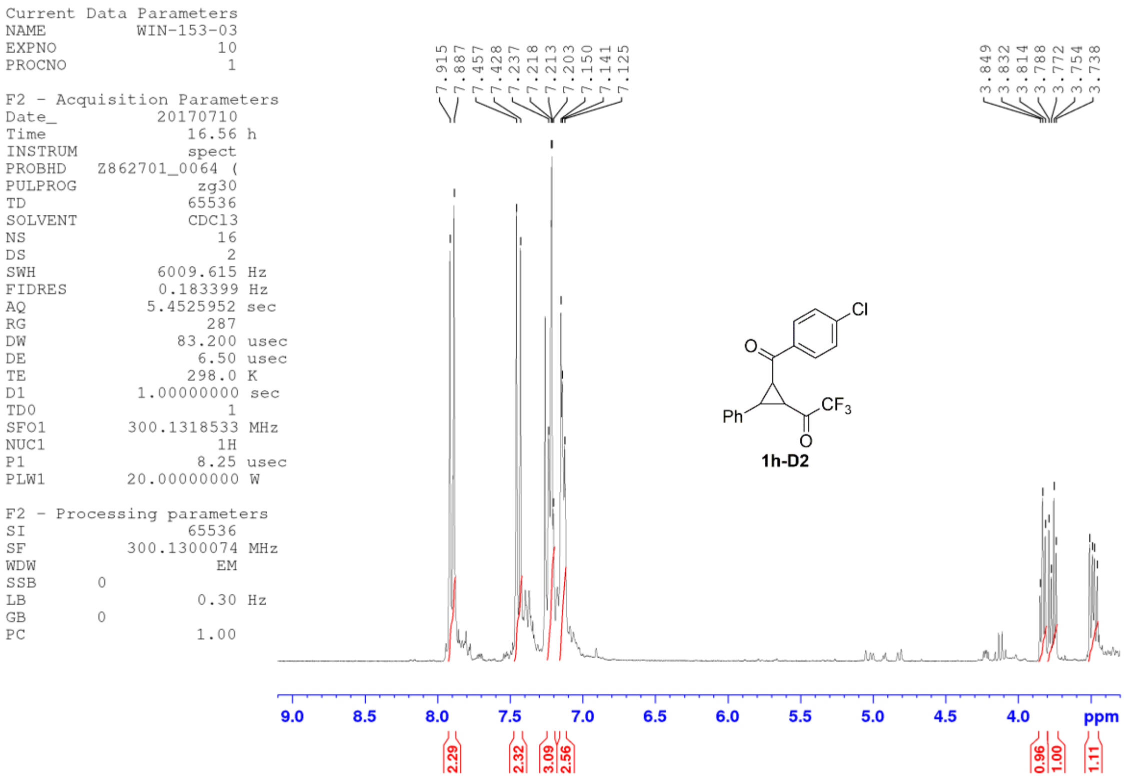
Current Data Parameters
NAME WIN-153-03
EXPNO 10
PROCNO 1
F2 - Acquisition Parameters
Date_ 20170710
Time 16.56 h
INSTRUM spect
PROBHD Z862701_0064 (
PULPROG zg30
TD 65536
SOLVENT CDCl3
NS 16
DS 2
SWH 6009.615 Hz
FIDRES 0.183399 Hz
AQ 5.4525952 sec
RG 287
DW 83.200 usec
DE 6.50 usec
TE 298.0 K
D1 1.00000000 sec
TD0 1
SFO1 300.1318533 MHz
NUC1 1H
P1 8.25 usec
PLW1 20.00000000 W
F2 - Processing parameters
SI 65536
SF 300.1300074 MHz
WDW EM
SSB 0
LB 0.30 Hz
GB 0
PC 1.00
7.915
7.887
7.457
7.428
7.237
7.218
7.213
7.203
7.150
7.141
7.125
3.849
3.832
3.814
3.788
3.772
3.754
3.738
Cl
O
CF_3
Ph
O
1h-D2
9.0
8.5
8.0
7.5
7.0
6.5
6.0
5.5
5.0
4.5
4.0
ppm
2.29
2.32
3.09
2.56
0.96
1.00
1.11

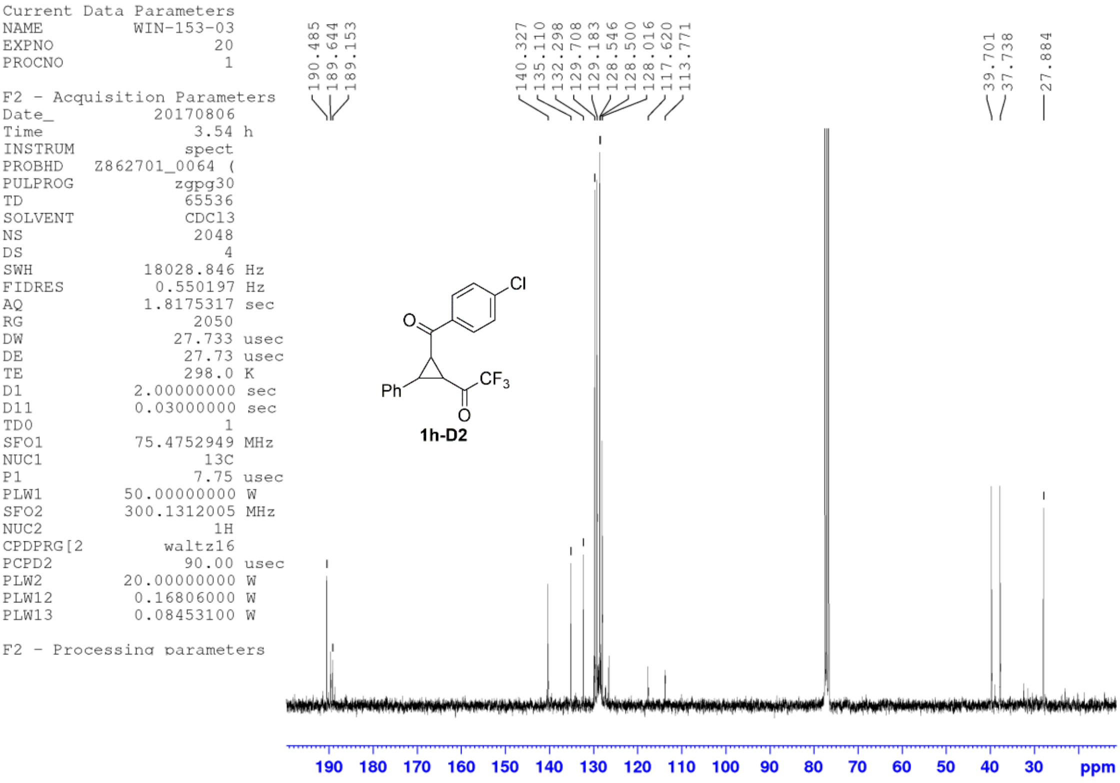
Current Data Parameters
NAME WIN-153-03
EXPNO 20
PROCNO 1
F2 - Acquisition Parameters
Date_ 20170806
Time 3.54 h
INSTRUM spect
PROBHD Z862701_0064 (
PULPROG zgpg30
TD 65536
SOLVENT CDCl3
NS 2048
DS 4
SWH 18028.846 Hz
FIDRES 0.550197 Hz
AQ 1.8175317 sec
RG 2050
DW 27.733 usec
DE 27.73 usec
TE 298.0 K
D1 2.00000000 sec
D11 0.03000000 sec
TD0 1
SFO1 75.4752949 MHz
NUC1 13C
P1 7.75 usec
PLW1 50.00000000 W
SFO2 300.1312005 MHz
NUC2 1H
CPDPRG[2 waltz16
PCPD2 90.00 usec
PLW2 20.00000000 W
PLW12 0.16806000 W
PLW13 0.08453100 W
F2 - Processing parameters
190.485
189.644
189.153
140.327
135.110
132.298
129.708
129.183
128.546
128.500
128.016
117.620
113.771
39.701
37.738
27.884
Cl
O
Ph
CF3
O
1h-D2
190 180 170 160 150 140 130 120 110 100 90 80 70 60 50 40 30 ppm

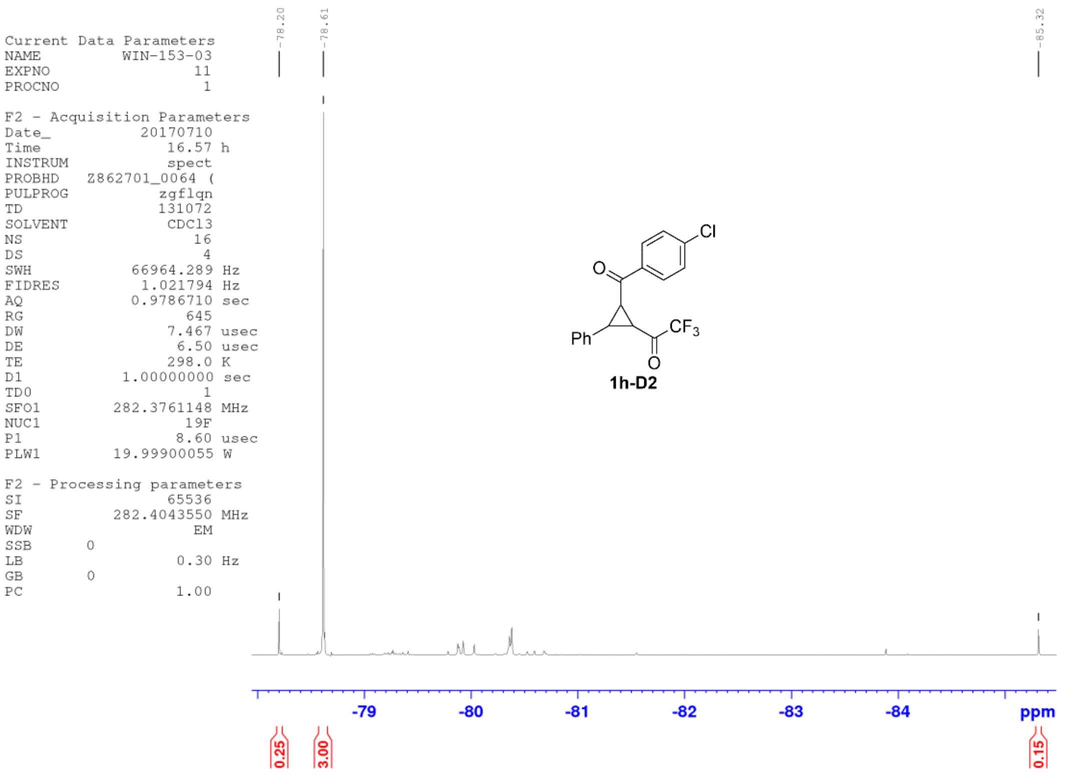
Current Data Parameters
NAME WIN-153-03
EXPNO 11
PROCNO 1
F2 - Acquisition Parameters
Date_ 20170710
Time 16.57 h
INSTRUM spect
PROBHD Z862701_0064 (
PULPROG zgflqn
TD 131072
SOLVENT CDCl3
NS 16
DS 4
SWH 66964.289 Hz
FIDRES 1.021794 Hz
AQ 0.9786710 sec
RG 645
DW 7.467 usec
DE 6.50 usec
TE 298.0 K
D1 1.00000000 sec
TD0 1
SFO1 282.3761148 MHz
NUC1 19F
P1 8.60 usec
PLW1 19.99900055 W
F2 - Processing parameters
SI 65536
SF 282.4043550 MHz
WDW EM
SSB 0
LB 0.30 Hz
GB 0
PC 1.00
-78.20
-78.61
-85.32
Cl
O
Ph
CF3
O
1h-D2
-79 -80 -81 -82 -83 -84 ppm
0.25
3.00
0.15

Current Data Parameters
NAME WIN-149-02
EXPNO 1
PROCNO 1

F2 - Acquisition Parameters
Date_ 20170921
Time 13.50 h
INSTRUM spect
PROBHD Z126715_0001 (
PULPROG zg30
TD 65536
SOLVENT CDCl3
NS 4
DS 2
SWH 9803.922 Hz
FIDRES 0.299192 Hz
AQ 3.3423359 sec
RG 18
DW 51.000 usec
DE 18.00 usec
TE 298.0 K
D1 2.00000000 sec
TD0 1
SFO1 700.3335017 MHz
NUC1 1H
P1 8.90 usec
PLW1 8.69999981 W

F2 - Processing parameters
SI 65536
SF 700.3300170 MHz
WDW EM
SSB 0
LB 1.00 Hz
GB 0
PC 1.00

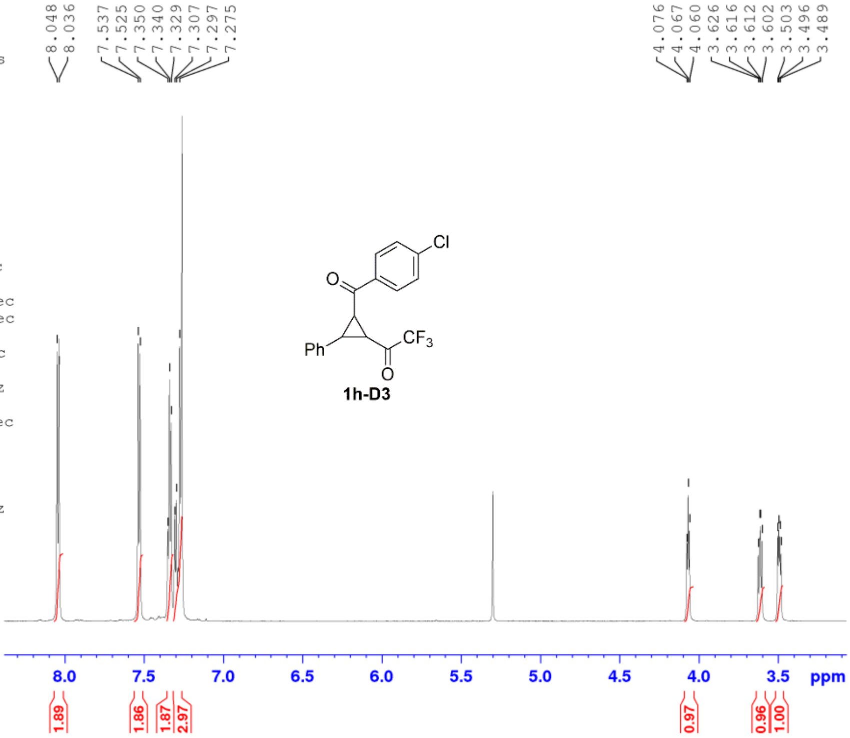

Current Data Parameters
NAME WIN-149-02
EXPNO 2
PROCNO 1

F2 - Acquisition Parameters
Date_ 20170921
Time 14.28 h
INSTRUM spect
PROBHD Z126715_0001 (
PULPROG zgpg30
TD 65536
SOLVENT CDCl3
NS 770
DS 4
SWH 40760.871 Hz
FIDRES 1.243923 Hz
AQ 0.8039083 sec
RG 2050
DW 12.267 usec
DE 18.00 usec
TE 298.0 K
D1 2.00000000 sec
D11 0.03000000 sec
TD0 1
SFO1 176.1183703 MHz
NUC1 13C
P1 12.00 usec
PLW1 129.00000000 W
SFO2 700.3328013 MHz
NUC2 1H
CPDPRG[2 waltz16
PCPD2 65.00 usec
PLW2 8.69999981 W
PLW12 0.16311000 W
PLW13 0.08213100 W

F2 - Processing parameters

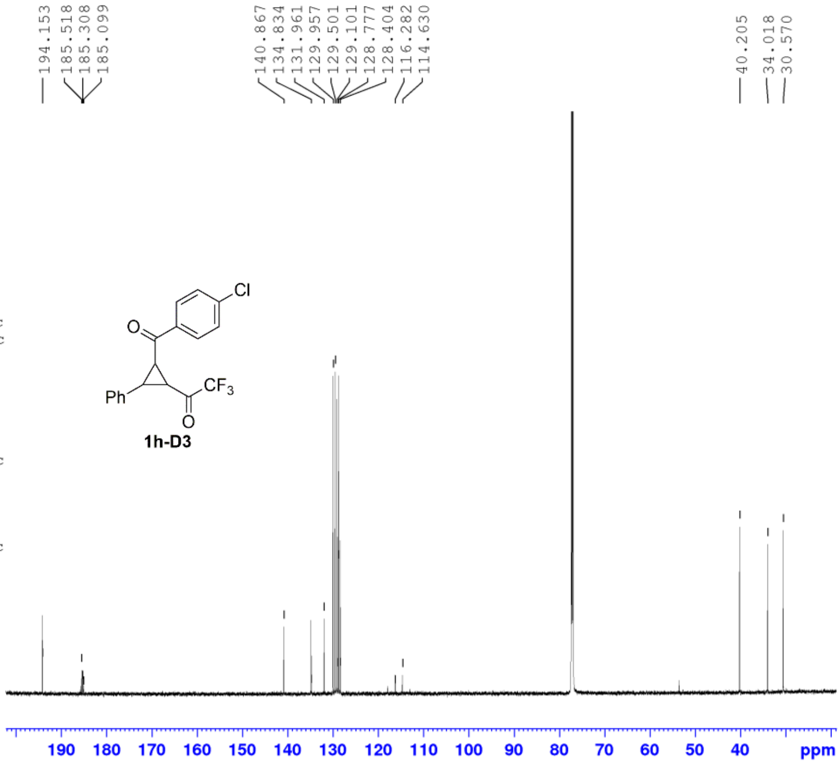

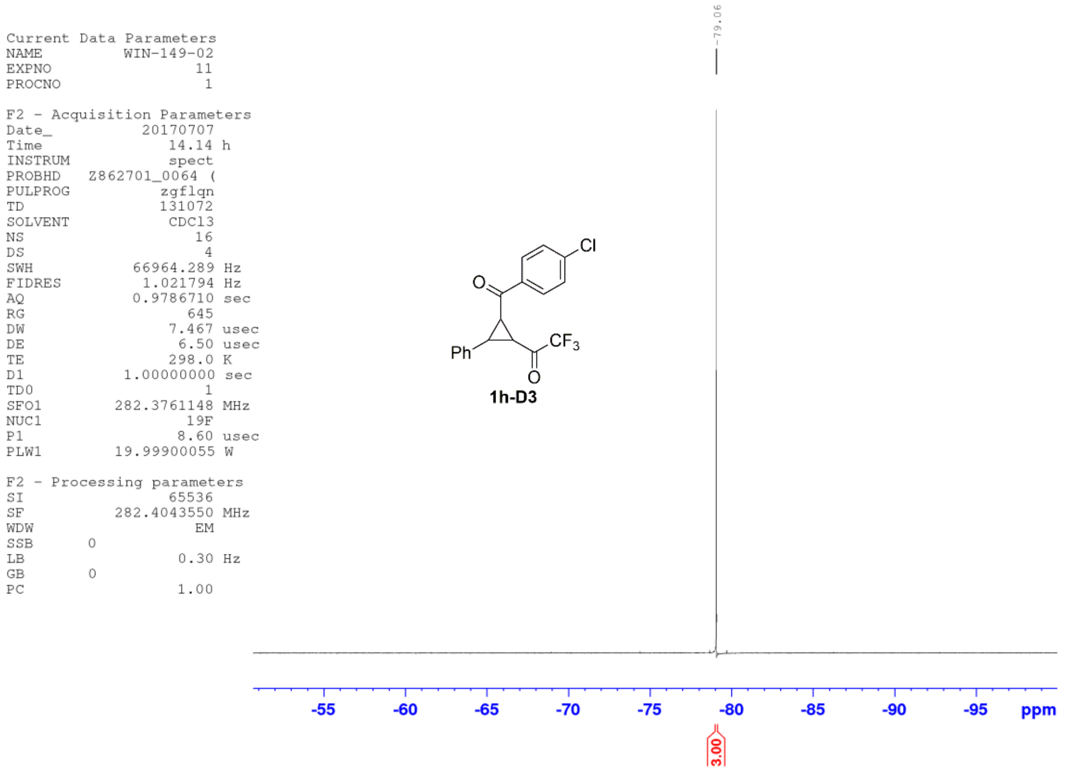

Current Data Parameters
NAME WIN-149-02
EXPNO 11
PROCNO 1
F2 - Acquisition Parameters
Date_ 20170707
Time 14.14 h
INSTRUM spect
PROBHD Z862701_0064 (
PULPROG zgflqn
TD 131072
SOLVENT CDCl3
NS 16
DS 4
SWH 66964.289 Hz
FIDRES 1.021794 Hz
AQ 0.9786710 sec
RG 645
DW 7.467 usec
DE 6.50 usec
TE 298.0 K
D1 1.00000000 sec
TD0 1
SFO1 282.3761148 MHz
NUC1 19F
P1 8.60 usec
PLW1 19.99900055 W
F2 - Processing parameters
SI 65536
SF 282.4043550 MHz
WDW EM
SSB 0
LB 0.30 Hz
GB 0
PC 1.00
-79.06
Cl
O
Ph
CF3
O
1h-D3
-55
-60
-65
-70
-75
-80
-85
-90
-95
ppm
3.00

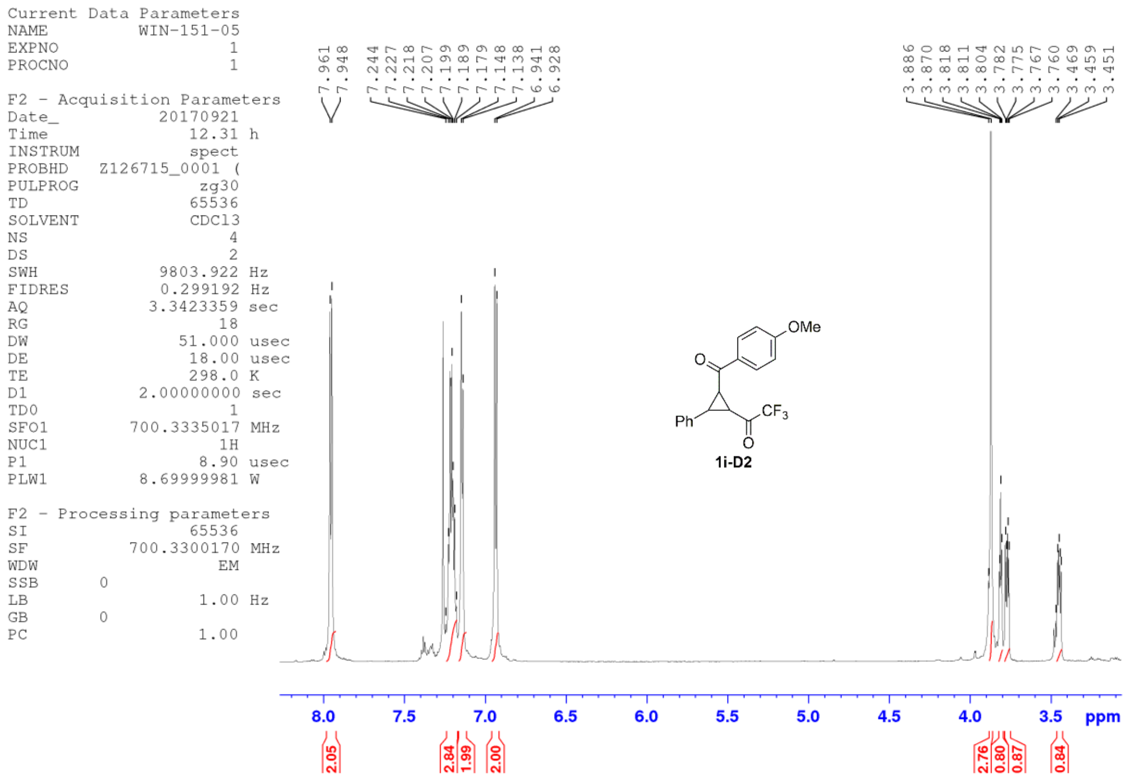

Current Data Parameters
NAME WIN-151-05
EXPNO 1
PROCNO 1
F2 - Acquisition Parameters
Date_ 20170921
Time 12.31 h
INSTRUM spect
PROBHD Z126715_0001 (
PULPROG zg30
TD 65536
SOLVENT CDCl3
NS 4
DS 2
SWH 9803.922 Hz
FIDRES 0.299192 Hz
AQ 3.3423359 sec
RG 18
DW 51.000 usec
DE 18.00 usec
TE 298.0 K
D1 2.00000000 sec
TD0 1
SFO1 700.3335017 MHz
NUC1 1H
P1 8.90 usec
PLW1 8.69999981 W
F2 - Processing parameters
SI 65536
SF 700.3300170 MHz
WDW EM
SSB 0
LB 1.00 Hz
GB 0
PC 1.00
7.961
7.948
7.244
7.227
7.218
7.207
7.199
7.189
7.179
7.148
7.138
6.941
6.928
3.886
3.870
3.818
3.811
3.804
3.782
3.775
3.767
3.760
3.469
3.459
3.451
OMe
O
Ph
CF3
O
1i-D2
8.0
7.5
7.0
6.5
6.0
5.5
5.0
4.5
4.0
3.5
ppm
2.05
2.84
1.99
2.00
2.76
0.80
0.87
0.84

```
Current Data Parameters
NAME          WIN-151-05
EXPNO                  2
PROCNO                 1

F2 - Acquisition Parameters
Date_           20170921
Time               12.58 h
INSTRUM            spect
PROBHD   Z126715_0001 (
PULPROG          zgpg30
TD                 65536
SOLVENT            CDCl3
NS                   522
DS                     4
SWH            40760.871 Hz
FIDRES          1.243923 Hz
AQ             0.8039083 sec
RG                   912
DW                12.267 usec
DE                 18.00 usec
TE                 298.0 K
D1            2.00000000 sec
D11           0.03000000 sec
TD0                    1
SFO1         176.1183703 MHz
NUC1                 13C
P1                 12.00 usec
PLW1        129.00000000 W
SFO2         700.3328013 MHz
NUC2                  1H
CPDPRG[2         waltz16
PCPD2              65.00 usec
PLW2          8.69999981 W
PLW12         0.16311000 W
PLW13         0.08213100 W

F2 - Processing parameters
```

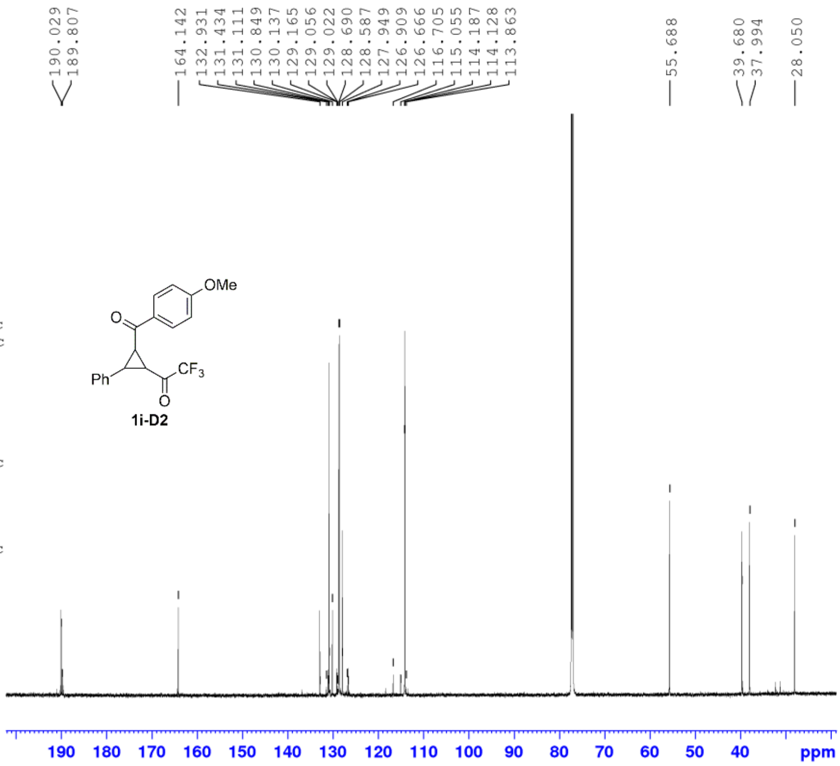


```
Current Data Parameters
NAME          WIN-151-05
EXPNO                 11
PROCNO                 1

F2 - Acquisition Parameters
Date_           20170710
Time               14.10 h
INSTRUM            spect
PROBHD   Z862701_0064 (
PULPROG           zgflqn
TD                131072
SOLVENT            CDCl3
NS                    16
DS                     4
SWH            66964.289 Hz
FIDRES          1.021794 Hz
AQ             0.9786710 sec
RG                   724
DW                 7.467 usec
DE                  6.50 usec
TE                 298.0 K
D1            1.00000000 sec
TD0                    1
SFO1         282.3761148 MHz
NUC1                 19F
P1                  8.60 usec
PLW1         19.99900055 W

F2 - Processing parameters
SI                 65536
SF           282.4043550 MHz
WDW                   EM
SSB      0
LB                  0.30 Hz
GB       0
PC                  1.00
```

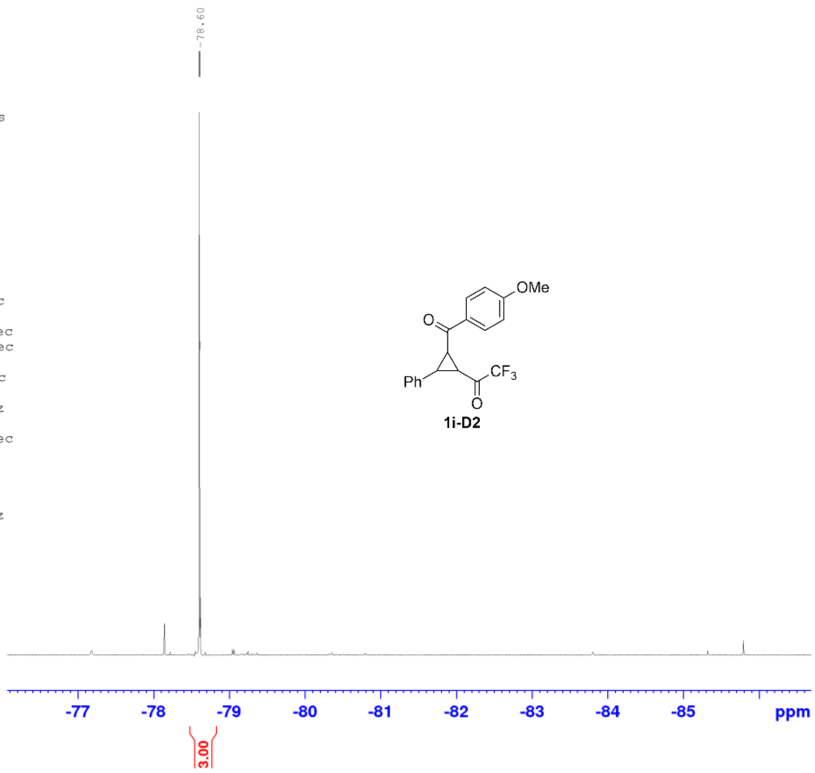

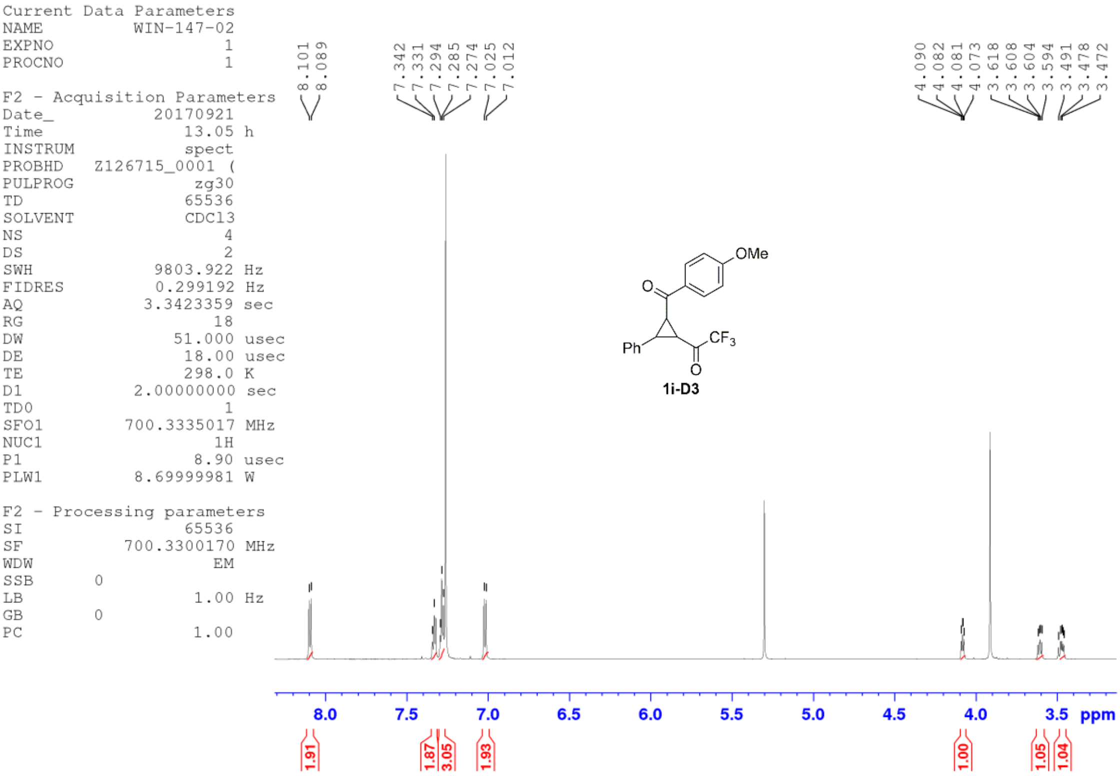
Current Data Parameters
NAME WIN-147-02
EXPNO 1
PROCNO 1
F2 - Acquisition Parameters
Date_ 20170921
Time 13.05 h
INSTRUM spect
PROBHD Z126715_0001 (
PULPROG zg30
TD 65536
SOLVENT CDCl3
NS 4
DS 2
SWH 9803.922 Hz
FIDRES 0.299192 Hz
AQ 3.3423359 sec
RG 18
DW 51.000 usec
DE 18.00 usec
TE 298.0 K
D1 2.00000000 sec
TD0 1
SFO1 700.3335017 MHz
NUC1 1H
P1 8.90 usec
PLW1 8.69999981 W
F2 - Processing parameters
SI 65536
SF 700.3300170 MHz
WDW EM
SSB 0
LB 1.00 Hz
GB 0
PC 1.00
8.101
8.089
7.342
7.331
7.294
7.285
7.274
7.025
7.012
4.090
4.082
4.081
4.073
3.618
3.608
3.604
3.594
3.491
3.478
3.472
OMe
O
Ph
CF3
O
1i-D3
8.0
7.5
7.0
6.5
6.0
5.5
5.0
4.5
4.0
3.5
ppm
1.91
1.87
3.05
1.93
1.00
1.05
1.04

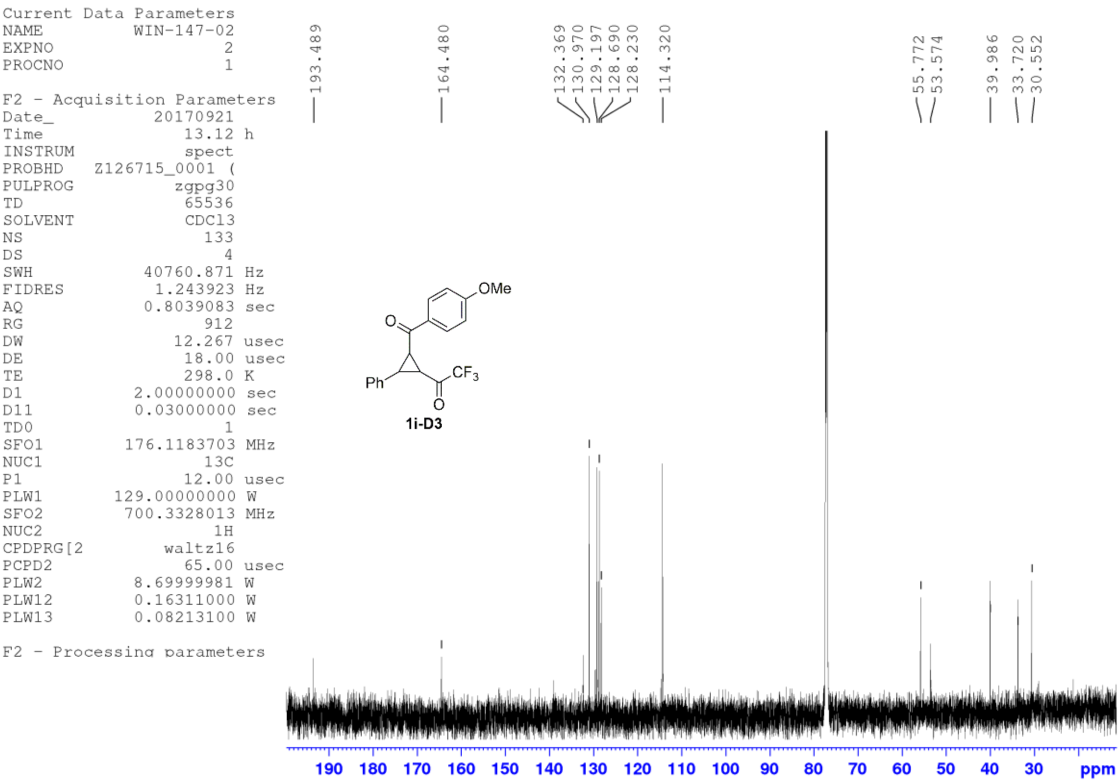
Current Data Parameters
NAME WIN-147-02
EXPNO 2
PROCNO 1
F2 - Acquisition Parameters
Date_ 20170921
Time 13.12 h
INSTRUM spect
PROBHD Z126715_0001 (
PULPROG zgpg30
TD 65536
SOLVENT CDCl3
NS 133
DS 4
SWH 40760.871 Hz
FIDRES 1.243923 Hz
AQ 0.8039083 sec
RG 912
DW 12.267 usec
DE 18.00 usec
TE 298.0 K
D1 2.00000000 sec
D11 0.03000000 sec
TD0 1
SFO1 176.1183703 MHz
NUC1 13C
P1 12.00 usec
PLW1 129.00000000 W
SFO2 700.3328013 MHz
NUC2 1H
CPDPRG[2 waltz16
PCPD2 65.00 usec
PLW2 8.69999981 W
PLW12 0.16311000 W
PLW13 0.08213100 W
F2 - Processing parameters
193.489
164.480
132.369
130.970
129.197
128.690
128.230
114.320
55.772
53.574
39.986
33.720
30.552
OMe
O
Ph
CF3
O
1i-D3
190
180
170
160
150
140
130
120
110
100
90
80
70
60
50
40
30
ppm

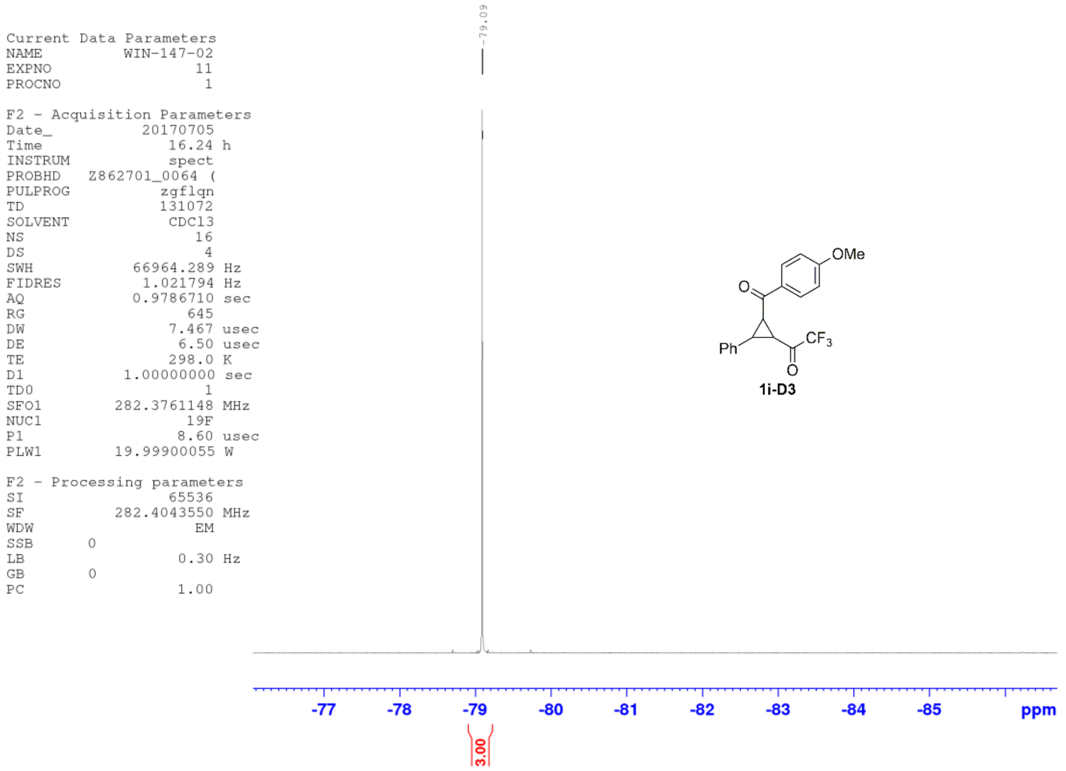
Current Data Parameters
NAME WIN-147-02
EXPNO 11
PROCNO 1
F2 - Acquisition Parameters
Date_ 20170705
Time 16.24 h
INSTRUM spect
PROBHD Z862701_0064 (
PULPROG zgflqn
TD 131072
SOLVENT CDCl3
NS 16
DS 4
SWH 66964.289 Hz
FIDRES 1.021794 Hz
AQ 0.9786710 sec
RG 645
DW 7.467 usec
DE 6.50 usec
TE 298.0 K
D1 1.00000000 sec
TD0 1
SFO1 282.3761148 MHz
NUC1 19F
P1 8.60 usec
PLW1 19.99900055 W
F2 - Processing parameters
SI 65536
SF 282.4043550 MHz
WDW EM
SSB 0
LB 0.30 Hz
GB 0
PC 1.00
-79.09
OMe
O
Ph
CF3
O
1i-D3
-77
-78
-79
-80
-81
-82
-83
-84
-85
ppm
3.00

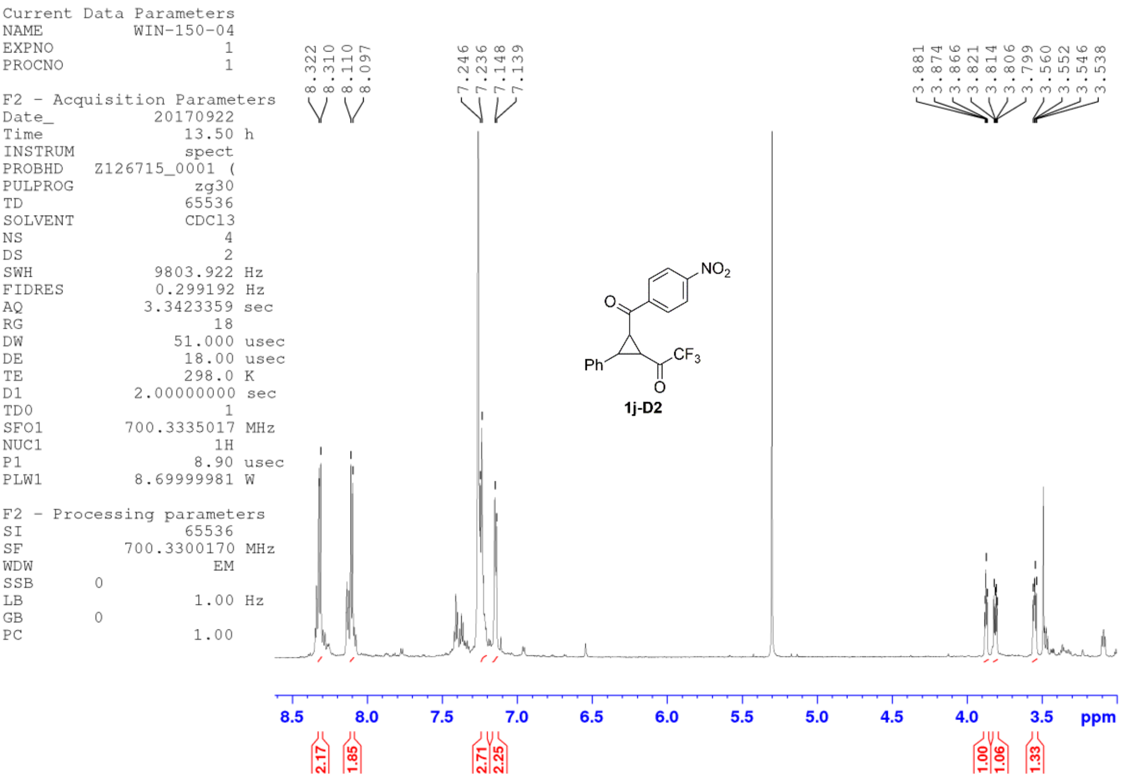
Current Data Parameters
NAME WIN-150-04
EXPNO 1
PROCNO 1
F2 - Acquisition Parameters
Date_ 20170922
Time 13.50 h
INSTRUM spect
PROBHD Z126715_0001 (
PULPROG zg30
TD 65536
SOLVENT CDCl3
NS 4
DS 2
SWH 9803.922 Hz
FIDRES 0.299192 Hz
AQ 3.3423359 sec
RG 18
DW 51.000 usec
DE 18.00 usec
TE 298.0 K
D1 2.00000000 sec
TD0 1
SFO1 700.3335017 MHz
NUC1 1H
P1 8.90 usec
PLW1 8.69999981 W
F2 - Processing parameters
SI 65536
SF 700.3300170 MHz
WDW EM
SSB 0
LB 1.00 Hz
GB 0
PC 1.00
8.322
8.310
8.110
8.097
7.246
7.236
7.148
7.139
3.881
3.874
3.866
3.821
3.814
3.806
3.799
3.560
3.552
3.546
3.538
NO2
O
Ph
CF3
O
1j-D2
8.5
8.0
7.5
7.0
6.5
6.0
5.5
5.0
4.5
4.0
3.5
ppm
2.17
1.85
2.71
2.25
1.00
1.06
1.33

```
Current Data Parameters
NAME          WIN-150-04
EXPNO                  2
PROCNO                 1

F2 - Acquisition Parameters
Date_           20170922
Time               14.53 h
INSTRUM            spect
PROBHD   Z126715_0001 (
PULPROG          zgpg30
TD                 65536
SOLVENT            CDCl3
NS                  1291
DS                     4
SWH            40760.871 Hz
FIDRES          1.243923 Hz
AQ             0.8039083 sec
RG                   912
DW                12.267 usec
DE                 18.00 usec
TE                 298.0 K
D1            2.00000000 sec
D11           0.03000000 sec
TD0                    1
SFO1         176.1183703 MHz
NUC1                 13C
P1                 12.00 usec
PLW1        129.00000000 W
SFO2         700.3328013 MHz
NUC2                  1H
CPDPRG[2         waltz16
PCPD2              65.00 usec
PLW2          8.69999981 W
PLW12         0.16311000 W
PLW13         0.08213100 W

F2 - Processing parameters
```

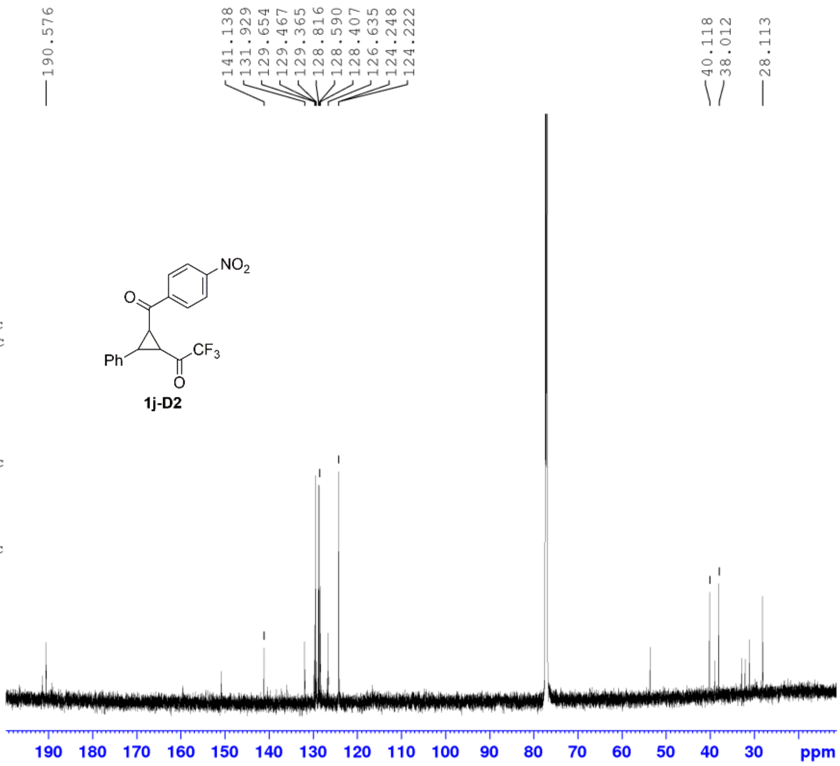


```
Current Data Parameters
NAME          WIN-150-04
EXPNO                 11
PROCNO                 1

F2 - Acquisition Parameters
Date_           20170710
Time               13.39 h
INSTRUM            spect
PROBHD   Z862701_0064 (
PULPROG           zgflqn
TD                131072
SOLVENT            CDCl3
NS                    16
DS                     4
SWH            66964.289 Hz
FIDRES          1.021794 Hz
AQ             0.9786710 sec
RG                   645
DW                 7.467 usec
DE                  6.50 usec
TE                 298.0 K
D1            1.00000000 sec
TD0                    1
SFO1         282.3761148 MHz
NUC1                 19F
P1                  8.60 usec
PLW1         19.99900055 W

F2 - Processing parameters
SI                 65536
SF           282.4043550 MHz
WDW                   EM
SSB      0
LB                  0.30 Hz
GB       0
PC                  1.00
```

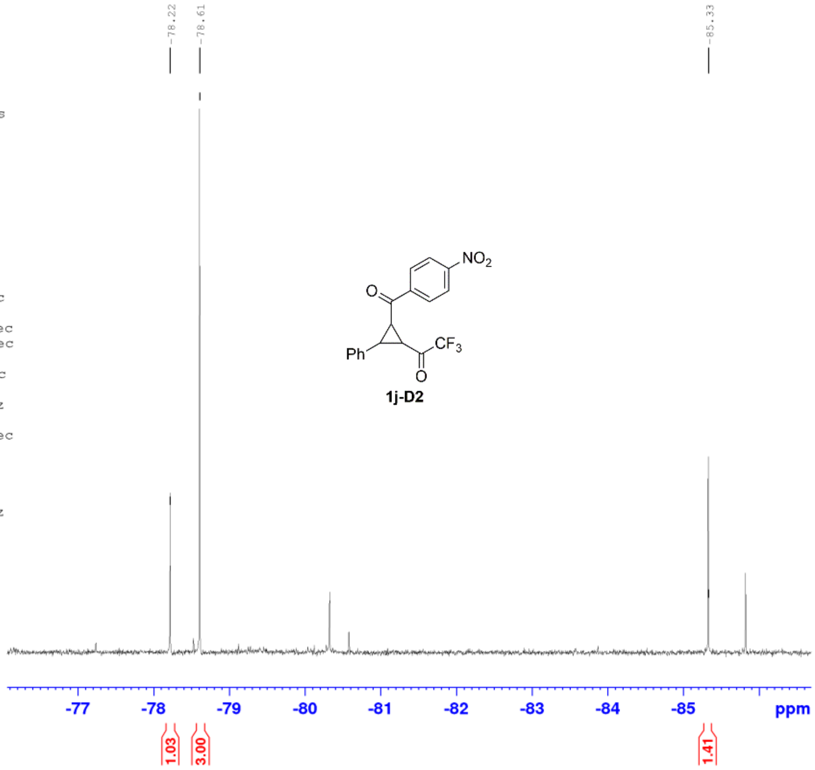

Current Data Parameters
NAME WIN-150-02
EXPNO 1
PROCNO 1

F2 - Acquisition Parameters
Date_ 20170922
Time 12.57 h
INSTRUM spect
PROBHD Z126715_0001 (
PULPROG zg30
TD 65536
SOLVENT CDCl3
NS 4
DS 2
SWH 9803.922 Hz
FIDRES 0.299192 Hz
AQ 3.3423359 sec
RG 18
DW 51.000 usec
DE 18.00 usec
TE 298.0 K
D1 2.00000000 sec
TD0 1
SFO1 700.3335017 MHz
NUC1 1H
P1 8.90 usec
PLW1 8.69999981 W

F2 - Processing parameters
SI 65536
SF 700.3300170 MHz
WDW EM
SSB 0
LB 1.00 Hz
GB 0
PC 1.00

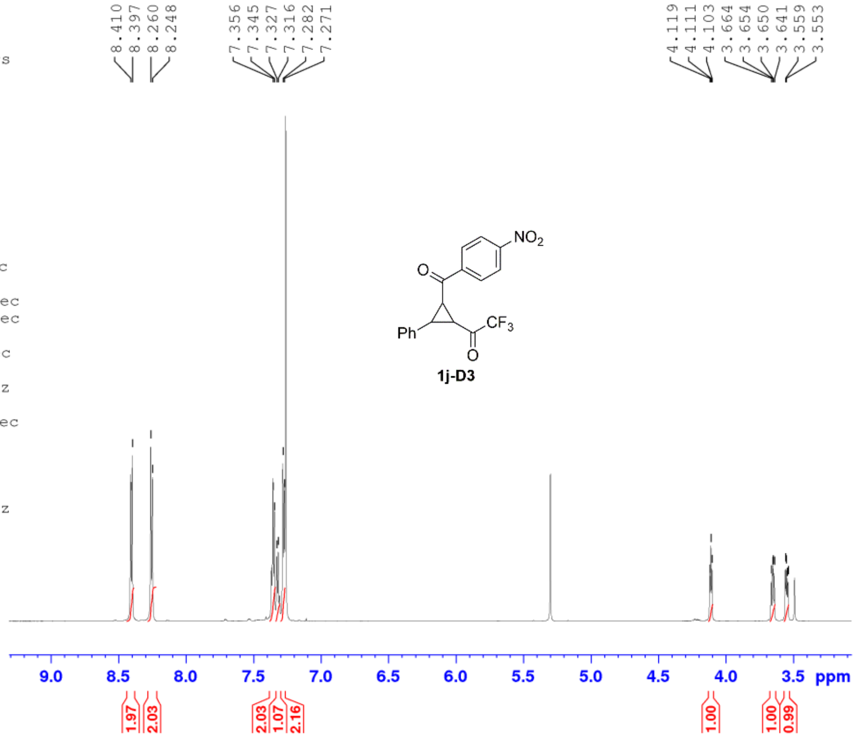

Current Data Parameters
NAME WIN-150-02
EXPNO 2
PROCNO 1

F2 - Acquisition Parameters
Date_ 20170922
Time 13.46 h
INSTRUM spect
PROBHD Z126715_0001 (
PULPROG zgpg30
TD 65536
SOLVENT CDCl3
NS 997
DS 4
SWH 40760.871 Hz
FIDRES 1.243923 Hz
AQ 0.8039083 sec
RG 912
DW 12.267 usec
DE 18.00 usec
TE 298.0 K
D1 2.00000000 sec
D11 0.03000000 sec
TD0 1
SFO1 176.1183703 MHz
NUC1 13C
P1 12.00 usec
PLW1 129.00000000 W
SFO2 700.3328013 MHz
NUC2 1H
CPDPRG[2 waltz16
PCPD2 65.00 usec
PLW2 8.69999981 W
PLW12 0.16311000 W
PLW13 0.08213100 W

F2 - Processing parameters

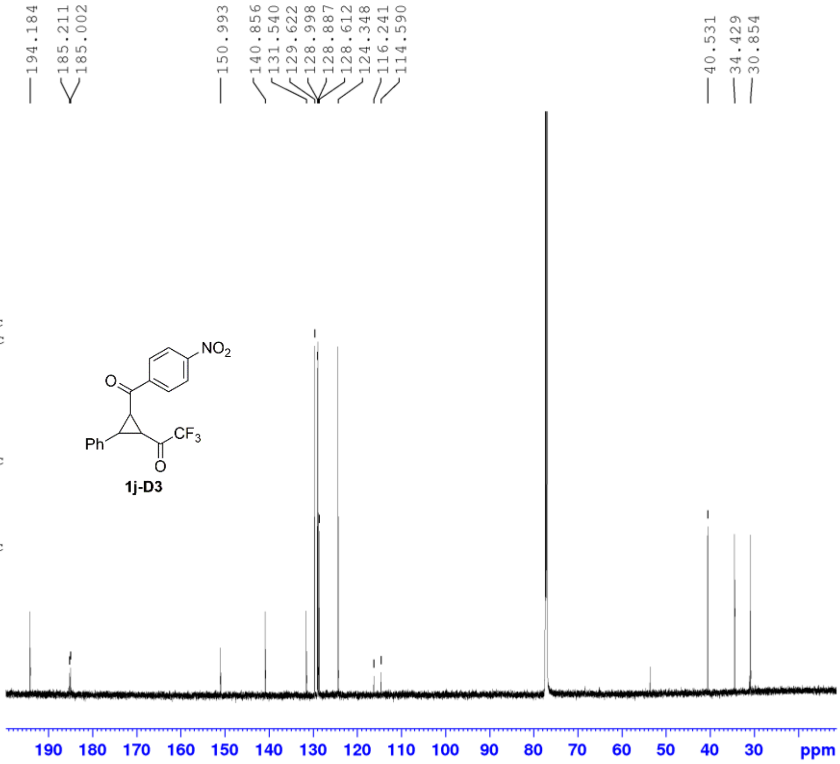

```
Current Data Parameters
NAME          WIN-150-02
EXPNO                 11
PROCNO                 1

F2 - Acquisition Parameters
Date_           20170710
Time               13.28 h
INSTRUM            spect
PROBHD   Z862701_0064 (
PULPROG          zgflqn
TD               131072
SOLVENT            CDCl3
NS                    16
DS                     4
SWH            66964.289 Hz
FIDRES          1.021794 Hz
AQ             0.9786710 sec
RG                   645
DW                 7.467 usec
DE                  6.50 usec
TE                 298.0 K
D1            1.00000000 sec
TD0                    1
SFO1         282.3761148 MHz
NUC1                 19F
P1                  8.60 usec
PLW1         19.99900055 W

F2 - Processing parameters
SI                 65536
SF           282.4043550 MHz
WDW                   EM
SSB      0
LB                  0.30 Hz
GB       0
PC                  1.00
```

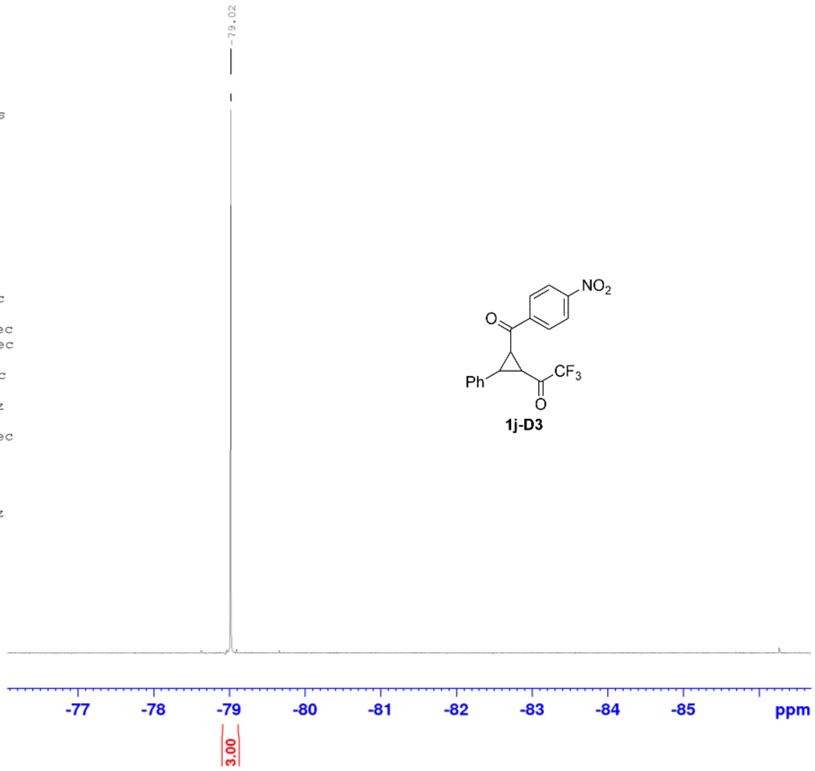


```
Current Data Parameters
NAME          WIN-183-01
EXPNO                  1
PROCNO                 1

F2 - Acquisition Parameters
Date_           20170922
Time                9.33 h
INSTRUM            spect
PROBHD   Z126715_0001 (
PULPROG             zg30
TD                 65536
SOLVENT            CDCl3
NS                     4
DS                     2
SWH             9803.922 Hz
FIDRES          0.299192 Hz
AQ             3.3423359 sec
RG                    18
DW                51.000 usec
DE                 18.00 usec
TE                 298.0 K
D1            2.00000000 sec
TD0                    1
SFO1         700.3335017 MHz
NUC1                  1H
P1                  8.90 usec
PLW1          8.69999981 W

F2 - Processing parameters
SI                 65536
SF           700.3300170 MHz
WDW                   EM
SSB      0
LB                  0.10 Hz
GB       0
PC                  1.00
```

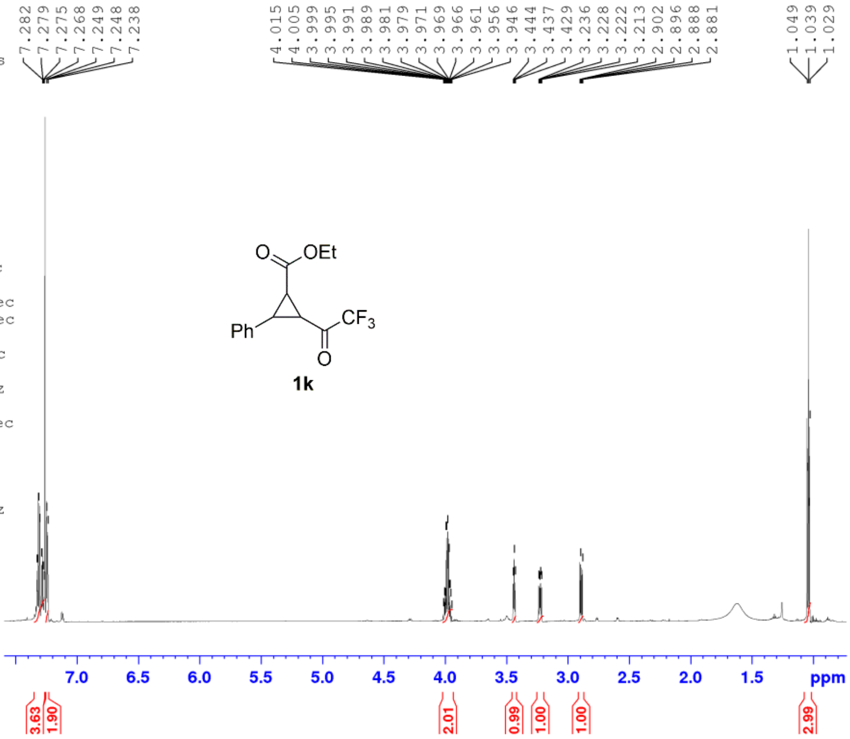

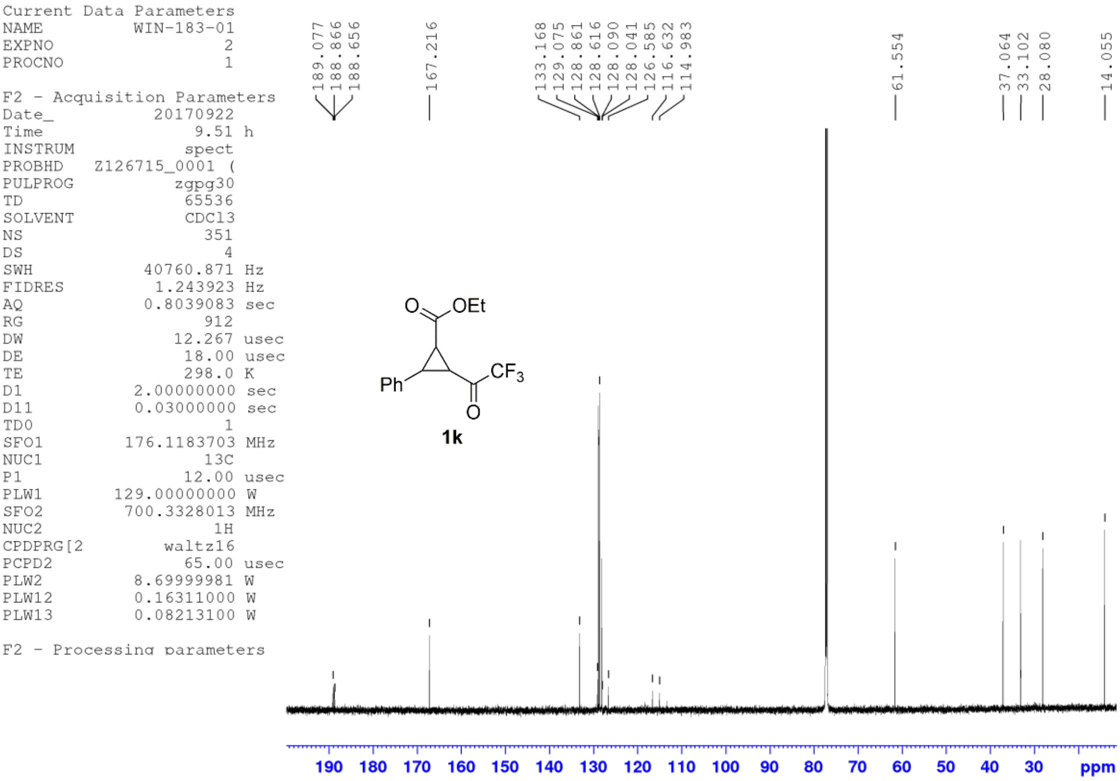
Current Data Parameters
NAME WIN-183-01
EXPNO 2
PROCNO 1
F2 - Acquisition Parameters
Date_ 20170922
Time 9.51 h
INSTRUM spect
PROBHD Z126715_0001 (
PULPROG zgpg30
TD 65536
SOLVENT CDCl3
NS 351
DS 4
SWH 40760.871 Hz
FIDRES 1.243923 Hz
AQ 0.8039083 sec
RG 912
DW 12.267 usec
DE 18.00 usec
TE 298.0 K
D1 2.00000000 sec
D11 0.03000000 sec
TD0 1
SFO1 176.1183703 MHz
NUC1 13C
P1 12.00 usec
PLW1 129.00000000 W
SFO2 700.3328013 MHz
NUC2 1H
CPDPRG[2 waltz16
PCPD2 65.00 usec
PLW2 8.69999981 W
PLW12 0.16311000 W
PLW13 0.08213100 W
F2 - Processing parameters
189.077
188.866
188.656
167.216
133.168
129.075
128.861
128.616
128.090
128.041
126.585
116.632
114.983
61.554
37.064
33.102
28.080
14.055
O
OEt
Ph
CF3
O
1k
190 180 170 160 150 140 130 120 110 100 90 80 70 60 50 40 30 ppm

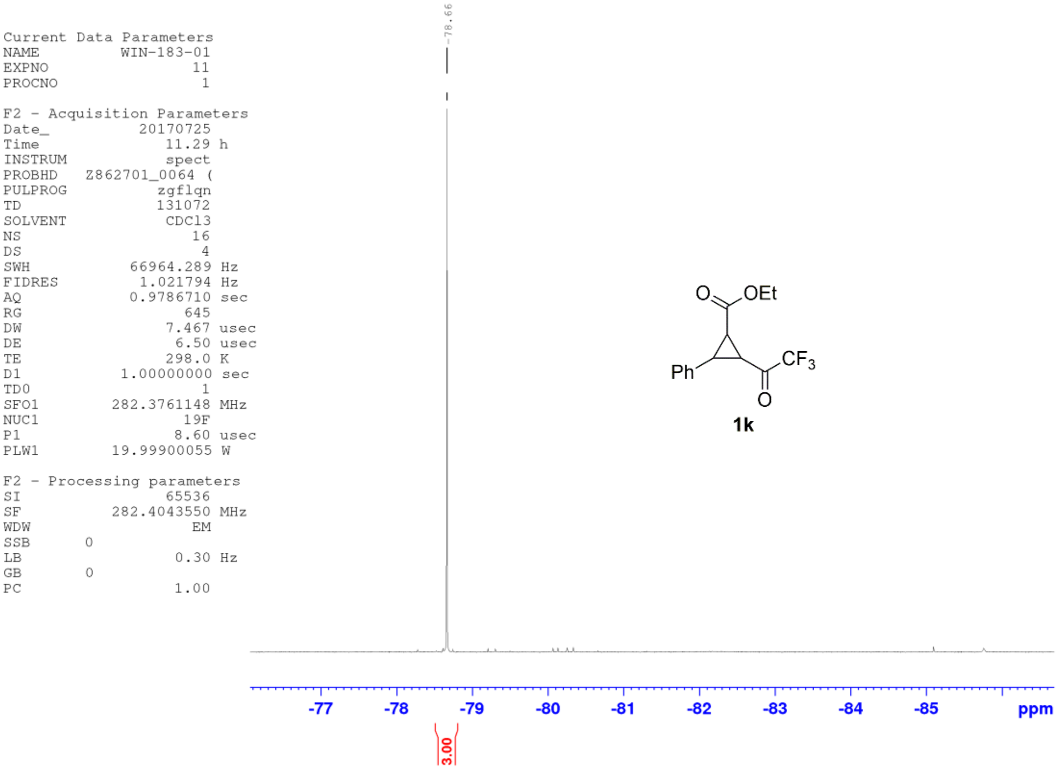
Current Data Parameters
NAME WIN-183-01
EXPNO 11
PROCNO 1
F2 - Acquisition Parameters
Date_ 20170725
Time 11.29 h
INSTRUM spect
PROBHD Z862701_0064 (
PULPROG zgflqn
TD 131072
SOLVENT CDCl3
NS 16
DS 4
SWH 66964.289 Hz
FIDRES 1.021794 Hz
AQ 0.9786710 sec
RG 645
DW 7.467 usec
DE 6.50 usec
TE 298.0 K
D1 1.00000000 sec
TD0 1
SFO1 282.3761148 MHz
NUC1 19F
P1 8.60 usec
PLW1 19.99900055 W
F2 - Processing parameters
SI 65536
SF 282.4043550 MHz
WDW EM
SSB 0
LB 0.30 Hz
GB 0
PC 1.00
-78.66
O
OEt
Ph
CF3
O
1k
-77 -78 -79 -80 -81 -82 -83 -84 -85 ppm
3.00
